# Supplementary material for: CD4+ T Cells Recognize Conserved Influenza A Epitopes through Shared Patterns of V-Gene Usage and Complementary Biochemical Features
Source: Cell Rep. 2020 Jul 14;32(2):107885. doi: 10.1016/j.celrep.2020.107885 (PMC7370177; doi:10.1016/j.celrep.2020.107885)
Supplement: Document S1. Figures S1–S7 and Data S1 [file mmc1.pdf]

Cell Reports, Volume *32*

Supplemental Information

CD4$^+$ T Cells Recognize Conserved Influenza A Epitopes through Shared Patterns of V-Gene Usage and Complementary Biochemical Features

Alexander Greenshields-Watson, Meriem Attaf, Bruce J. MacLachlan, Thomas Whalley, Cristina Rius, Aaron Wall, Angharad Lloyd, Hywel Hughes, Kathryn E. Strange, Georgina H. Mason, Andrea J. Schauenburg, Sarah L. Hulin-Curtis, James Geary, Yuan Chen, Sarah N. Lauder, Kathryn Smart, Dhanasekaran Vijaykrishna, Miguel L. Grau, Mikhail Shugay, Robert Andrews, Garry Dolton, Pierre J. Rizkallah, Awen M. Gallimore, Andrew K. Sewell, Andrew J. Godkin, and David K. Cole

A

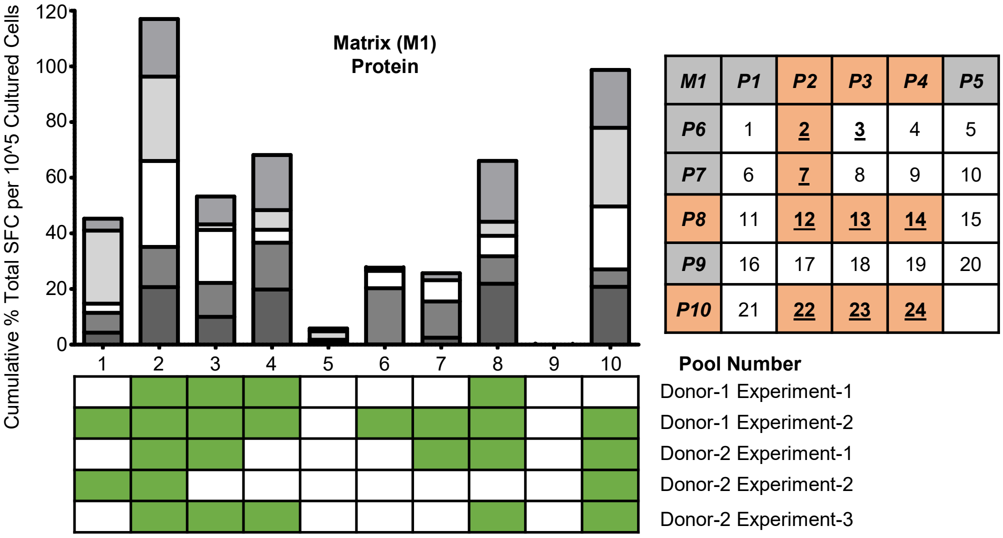

M1	P1	P2	P3	P4	P5
P6	1	**2**	**3**	4	5
P7	6	**7**	8	9	10
P8	11	**12**	**13**	**14**	15
P9	16	17	18	19	20
P10	21	**22**	**23**	**24**	

Identification of putatively immunogenic peptides within peptide pools. Peptide pools which elicited positive responses were cross referenced to reveal the common peptide.

B

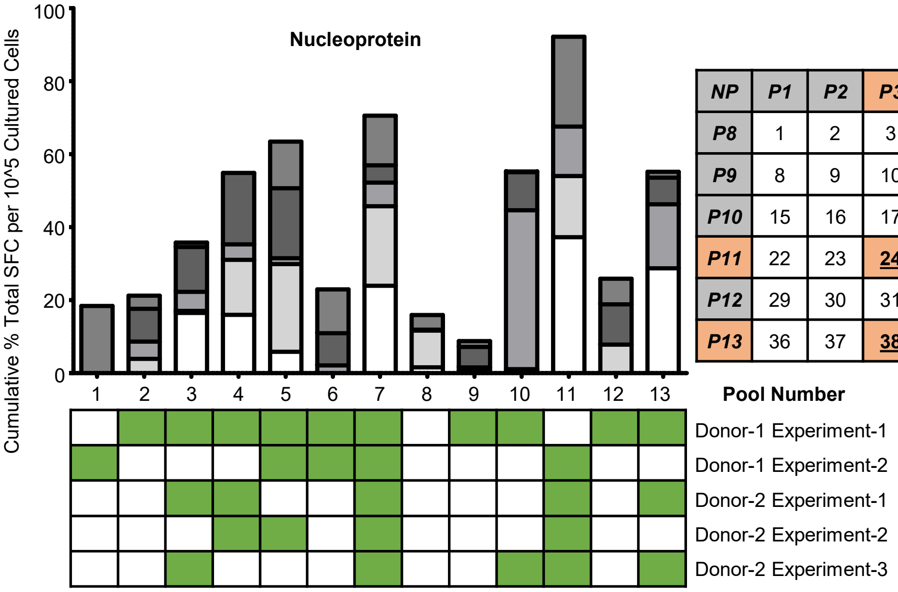

NP	P1	P2	P3	P4	P5	P6	P7
P8	1	2	3	4	5	6	7
P9	8	9	10	11	12	13	14
P10	15	16	17	18	19	20	21
P11	22	23	**24**	**25**	**26**	27	**28**
P12	29	30	31	32	33	34	35
P13	36	37	**38**	**39**			

C

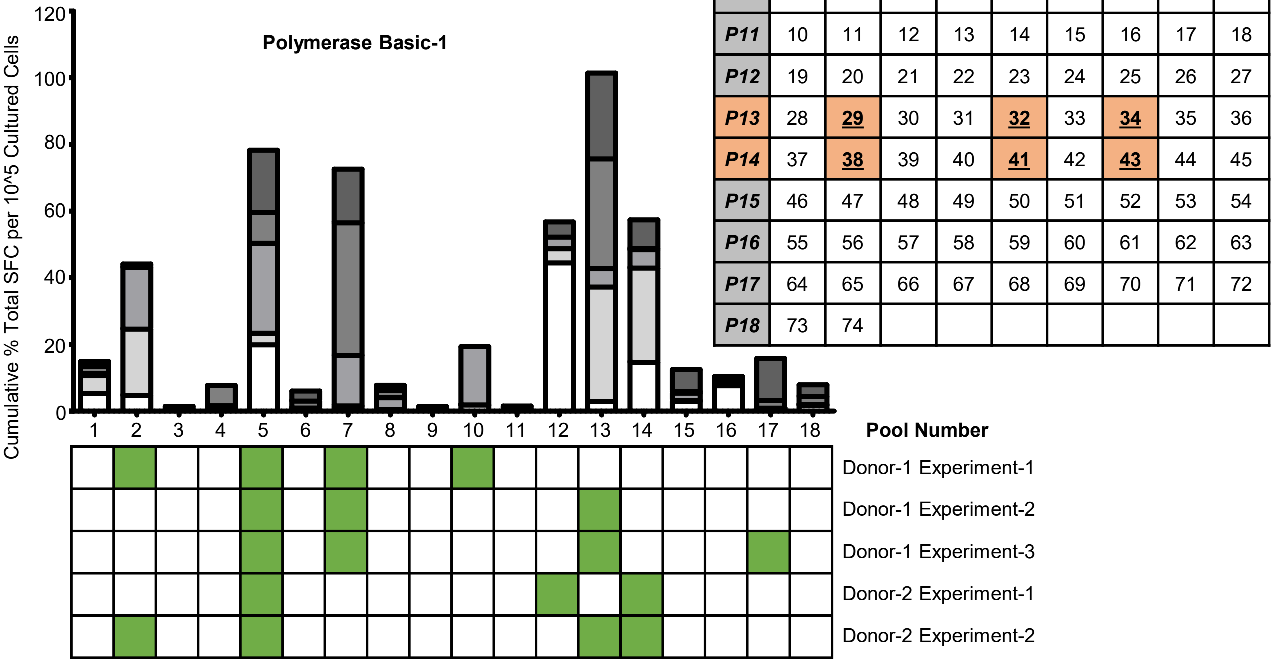

PB1	P1	P2	P3	P4	P5	P6	P7	P8	P9
P10	1	2	3	4	5	6	7	8	9
P11	10	11	12	13	14	15	16	17	18
P12	19	20	21	22	23	24	25	26	27
P13	28	**29**	30	31	**32**	33	**34**	35	36
P14	37	**38**	39	40	**41**	42	**43**	44	45
P15	46	47	48	49	50	51	52	53	54
P16	55	56	57	58	59	60	61	62	63
P17	64	65	66	67	68	69	70	71	72
P18	73	74							

Figure S1. Summary of epitope mapping assays using peptide pools, Related to Figure 1. Results are shown by pane for each internal protein: M1-matrix (**A**), NP-nucleoprotein (**B**) and PB1-polymerase basic-1 protein (**C**). Peptide 20-mers (numbered 1, 2, 3… in the array/matrix format) overlapping by 10 amino acids were arranged into pools, and designed such that each peptide was only found in a unique combination of two pools (arrays for each protein are detailed with pool number (P1, P2…) on the top row and down the left column in gray). Each pool specific SFC result was normalised by dividing by the total number of SFC across all peptide pools in that assay to give a percentage value. Percentage values are stacked for each pool to give a cumulative representation of the responses across multiple experiments (assay repeats on blood taken at different timepoints) in two donors. The results of each experiment are summarised in a grid below the bar chart, with the response to a pool filled in green if the SFC number was defined as a positive result (greater than 20 SFC per 100,000 PBMC). The pools which were defined as immunogenic (based on shared immunogenicity in each donor), are indicated by the colour orange on the array to the right-hand side of each bar chart. Cross-referencing the array of positive pools indicated the peptides to be investigated individually (numbers underlined in orange boxes). Additional peptides were also tested that were not explicitly highlighted in two cross referenced pools but were part of a highly immunogenic pool that warranted further investigation (also underlined in orange lined boxes).

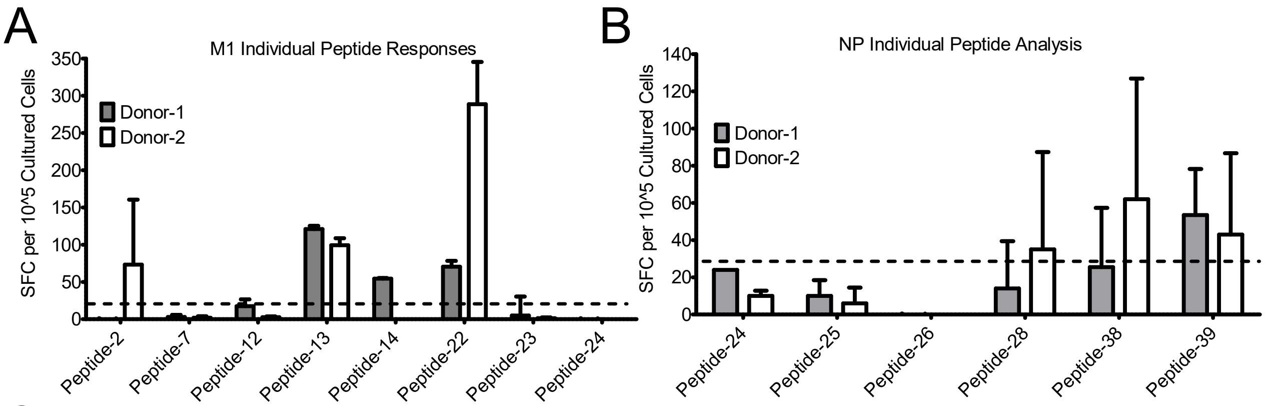

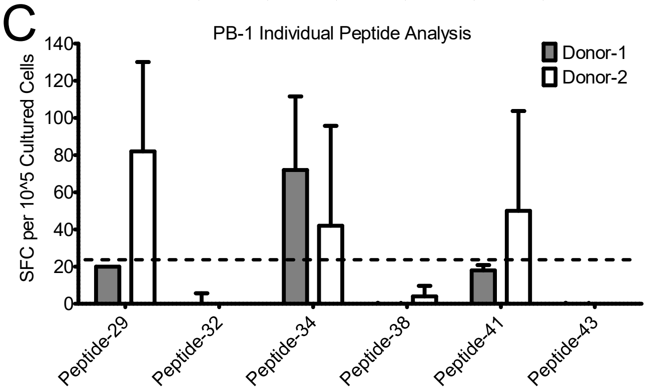

D

	Short Peptide Sequences
**M1$_{17-30}$**	SGPLKAEIAQRLED
**M1$_{129-142}$**	GLIYNRMGAVTTEV
**M1$_{208-222}$**	QARQMVQAMRTIGTHP
**NP$_{302-314}$**	DPFRLLQNSQVFS
**NP$_{417-430}$**	LPFERATIMAAFT
**NP$_{409-421}$**	PTFSVQRNLPFER
**PB-1$_{284-297}$**	NVVRKMMTNSQDT
**PB-1$_{342-354}$**	IMFSNKMARLGKG
**PB-1$_{410-422}$**	GMFNMLSTVLGVS

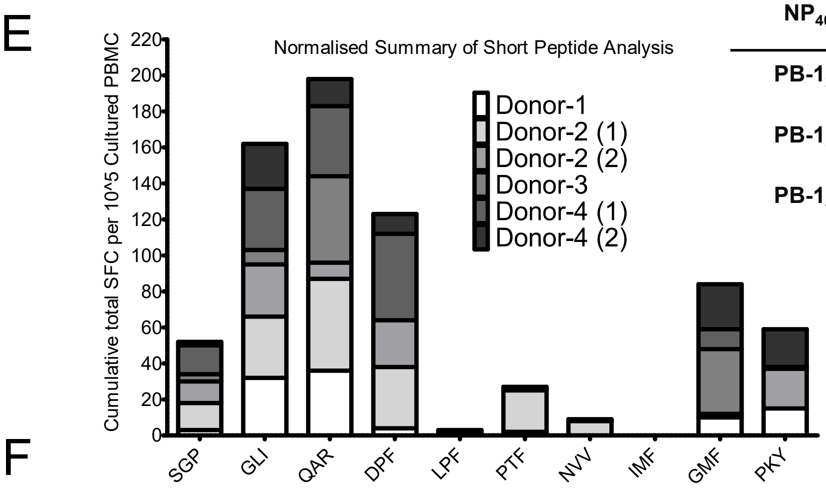

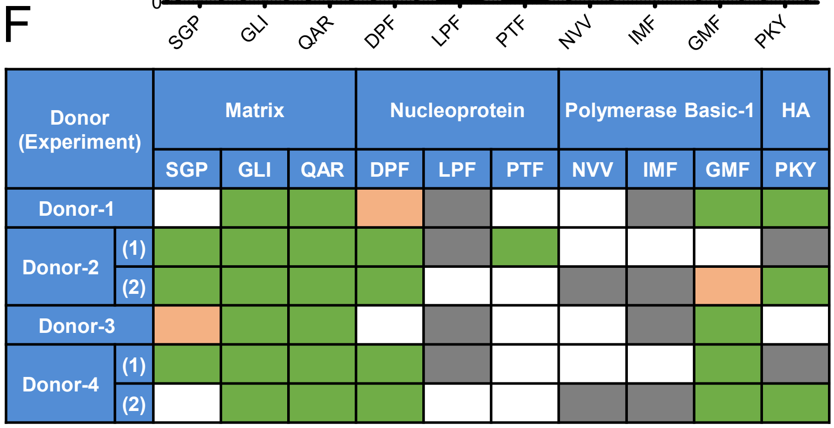

Figure S2. Individual peptide analyses of regions identified from pool assays on HLA-DR1 IFN-γ ELISpot, Related to Figure 1. (**A**) Matrix-1, (**B**) Nucleoprotein, (**C**) Polymerase Basic-1. For each assay, a line cultured against a parent pool, and shown to be reactive to that pool on IFN-γ ELISpot, was then retested with specific individual peptides from that pool. Due to limited numbers of PBMC and a broad range of testing that occurred, not all peptides were tested equal numbers of times in each donor (mean with SD error bars, donor 1: n = 2, donor 2: n = 3). Based on these results and predictions from NetMHCIIpan, short peptide sequences were designed (**D**), and used for further testing in the rest of the study. Lines were cultured with these short

peptides across 4 HLA-DR1+ donors and cumulative analysis on normalised SFC is shown (**E**), where each response was dividing by the total number of SFC across all peptide tested in that particular assay to give a percentage value. Percentage vales are stacked to give a cumulative representation of the responses across multiple assays in four donors. (**F**) Representation of each assay, with the response to a specific peptide filled in green if the SFC number was a positive result (greater than 20 SFC per 100,000 PBMC). Boxes are orange if a response was borderline (of two replicates one was just above the significance level and one was just below, but the mean was below 20 SFC). White indicates no response. Dark grey indicates not tested.

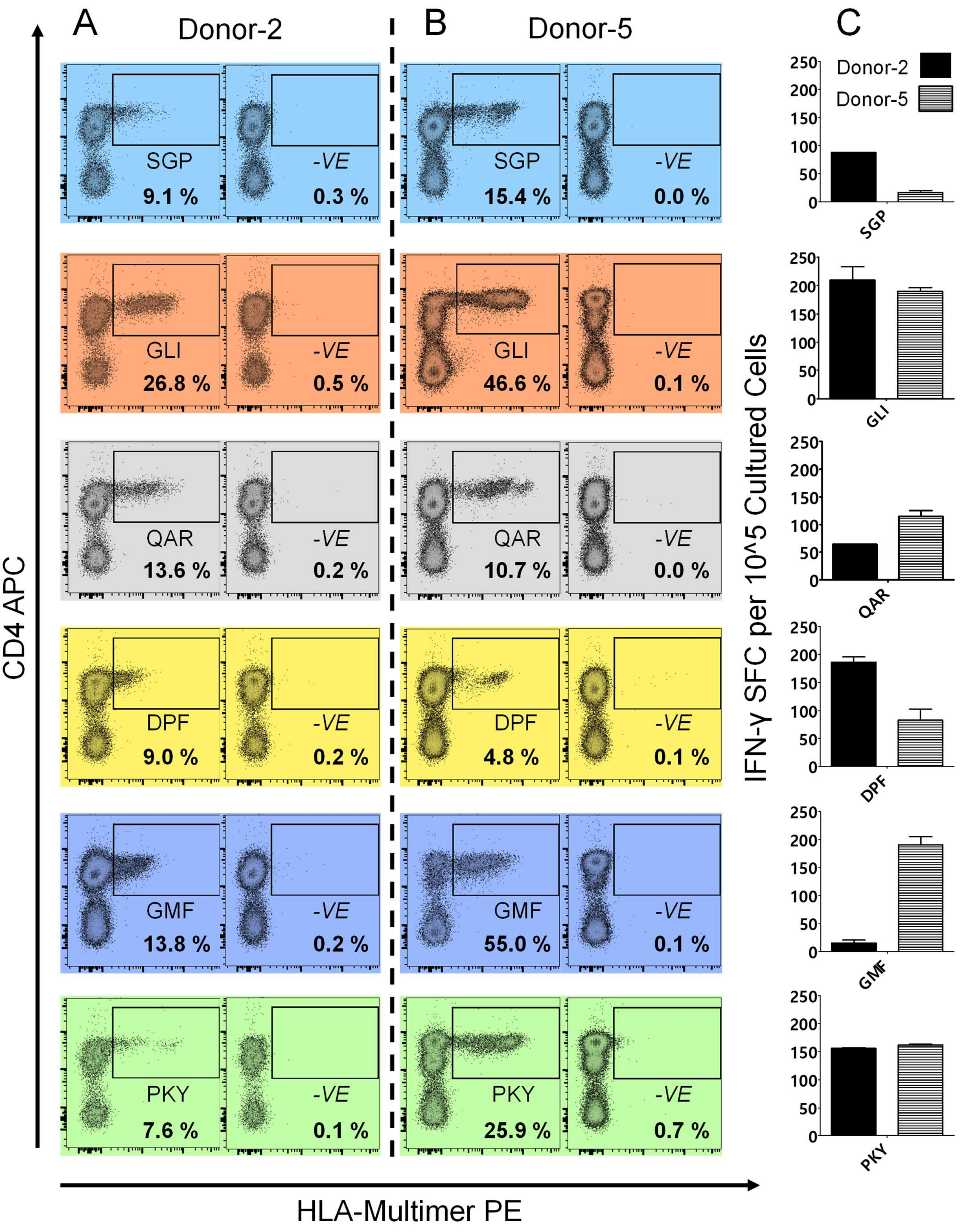

Figure S3. Comparison of HLA-Multimer staining and IFN-γ ELISpot responses in two HLA-DR1⁺ donors, Related to Figure 2. Donor numbers correspond to those shown in Fig. 7 of the manuscript. HLA-multimer stains are shown alongside irrelevant HLA Class-II multimer negative (-VE) controls for donor-2 (**A**) and donor-5 (**B**) with % of CD4+ T-cells shown for each gate. Data for each epitope is shown as a colour-coded row. (**C**) IFN-γ ELISpot data for each donor and epitope is displayed

as SFC per 100,000 PBMC with background (negative control) subtracted, donor-2 in black, donor-5 in hatched bars (mean with SD error bars, n = 2).

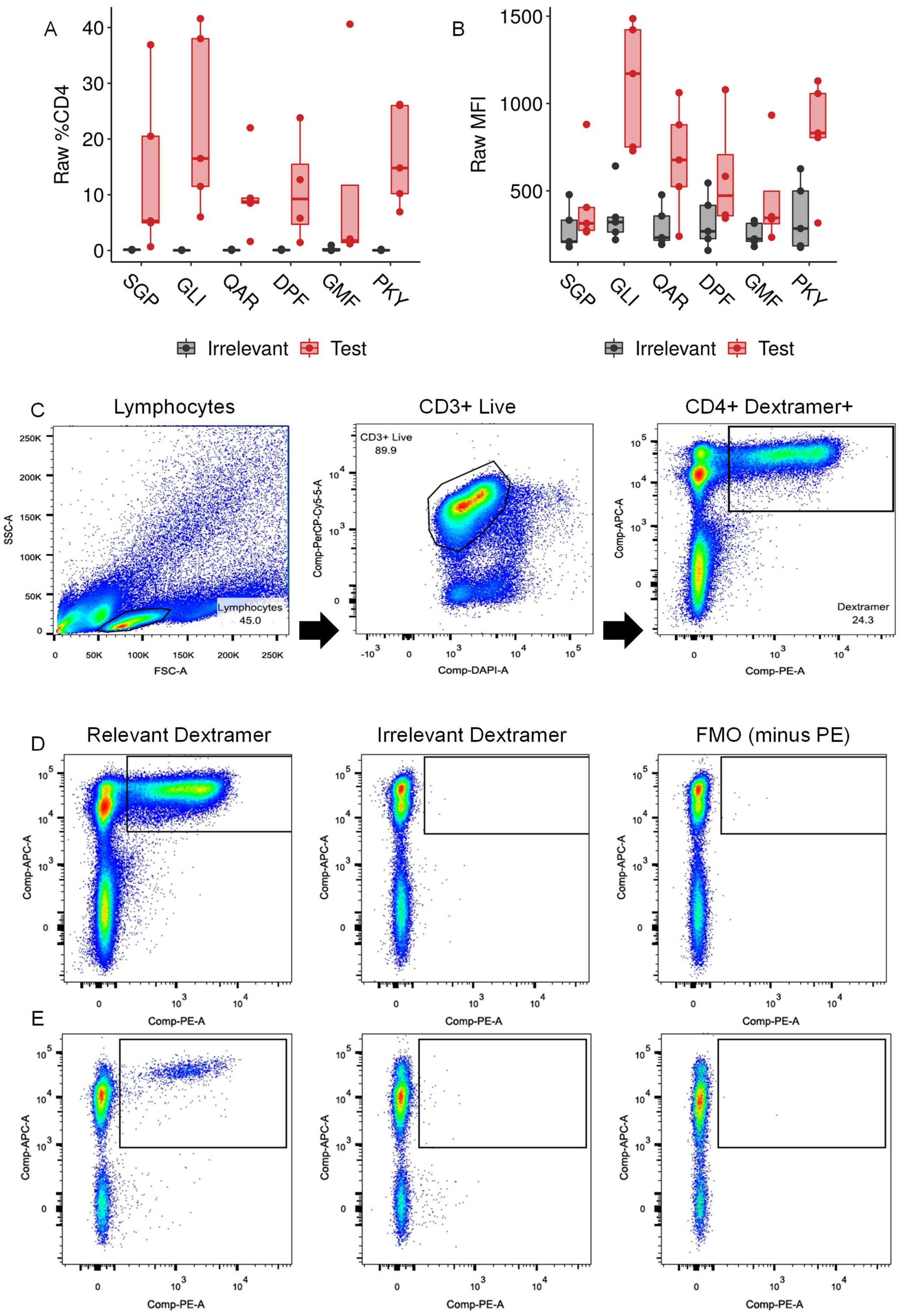

A
Raw %CD4
SGP GLI QAR DPF GMF PKY
Irrelevant Test
B
Raw MFI
SGP GLI QAR DPF GMF PKY
Irrelevant Test
C
Lymphocytes
Lymphocytes 45.0
SSC-A
FSC-A
CD3+ Live
CD3+ Live 89.9
Comp-PerCP-Cy5-5-A
Comp-DAPI-A
CD4+ Dextramer+
Dextramer 24.3
Comp-APC-A
Comp-PE-A
D
Relevant Dextramer
Irrelevant Dextramer
FMO (minus PE)
Comp-APC-A
Comp-PE-A
E
Comp-APC-A
Comp-PE-A

Figure S4. Additional flow cytometry data: human in vitro raw values, gating strategy and examples of irrelevant control and fluorescence minus one (FMO) stains, Related to Figure 2. Data corresponding to human in vitro staining shown in Fig. 2A-C. (**A**) Epitope-specific cells as a %$CD4^+$ T cells, box plots show median, and interquartile range. (**B**) Corresponding MFI (median fluorescence intensity), values for epitope-specific populations. (**C**) Gating strategy to identify antigen specific CD4+ T-cells. (**D-E**) Example staining showing two examples of the same population stained with relevant dextramer, control irrelevant class-II dextramer (used to define the dextramer gate) and the fluorescence minus one control.

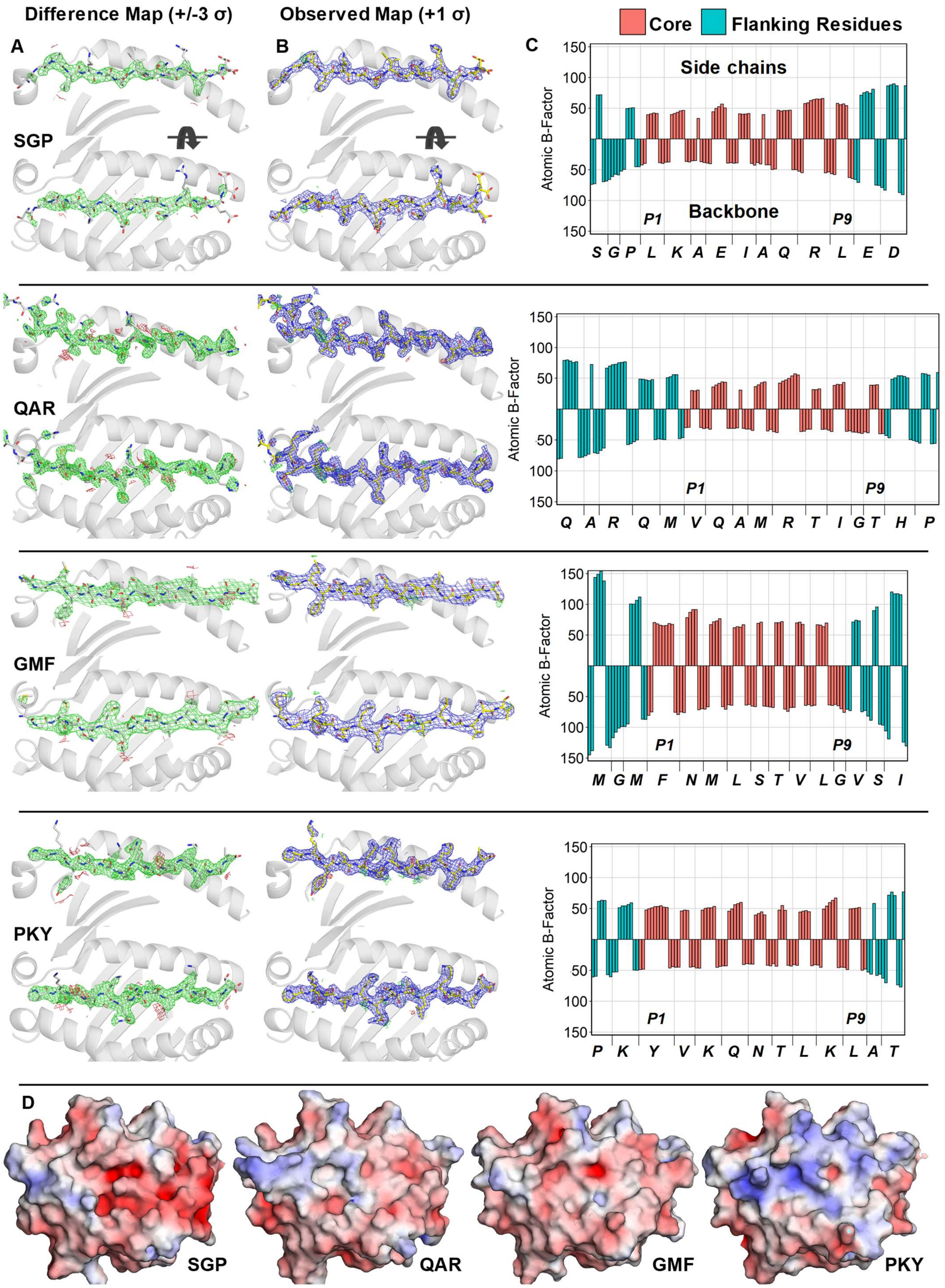

Figure S5. Electron Density of Conserved HLA-DR1 Flu Epitopes, Related to Figure 1. Column **(A)** shows the omit maps (FoFc) around each peptide in two orientations. Column **(B)** shows the final electron density (2FoFc) at the end of refinement. Observed density is displayed at 1 σ contour level, in blue. positive difference density at +3 σ is shown in green and negative difference density is shown in red at -3 σ. Column **(C)** shows a bar chart representing individual B-factors per non-H atom of the

peptide. Main chain atoms (N, Cα, C, O) bars are pointing downwards; side chains bars are pointing upwards. Panel **(D)** shows the electrostatic surface potential, calculated with PyMOL 2.0 plug-in APBS (Baker et al., 2001). Red areas are overall negatively charged, blue areas are positively charged, and grey/white areas are neutral.

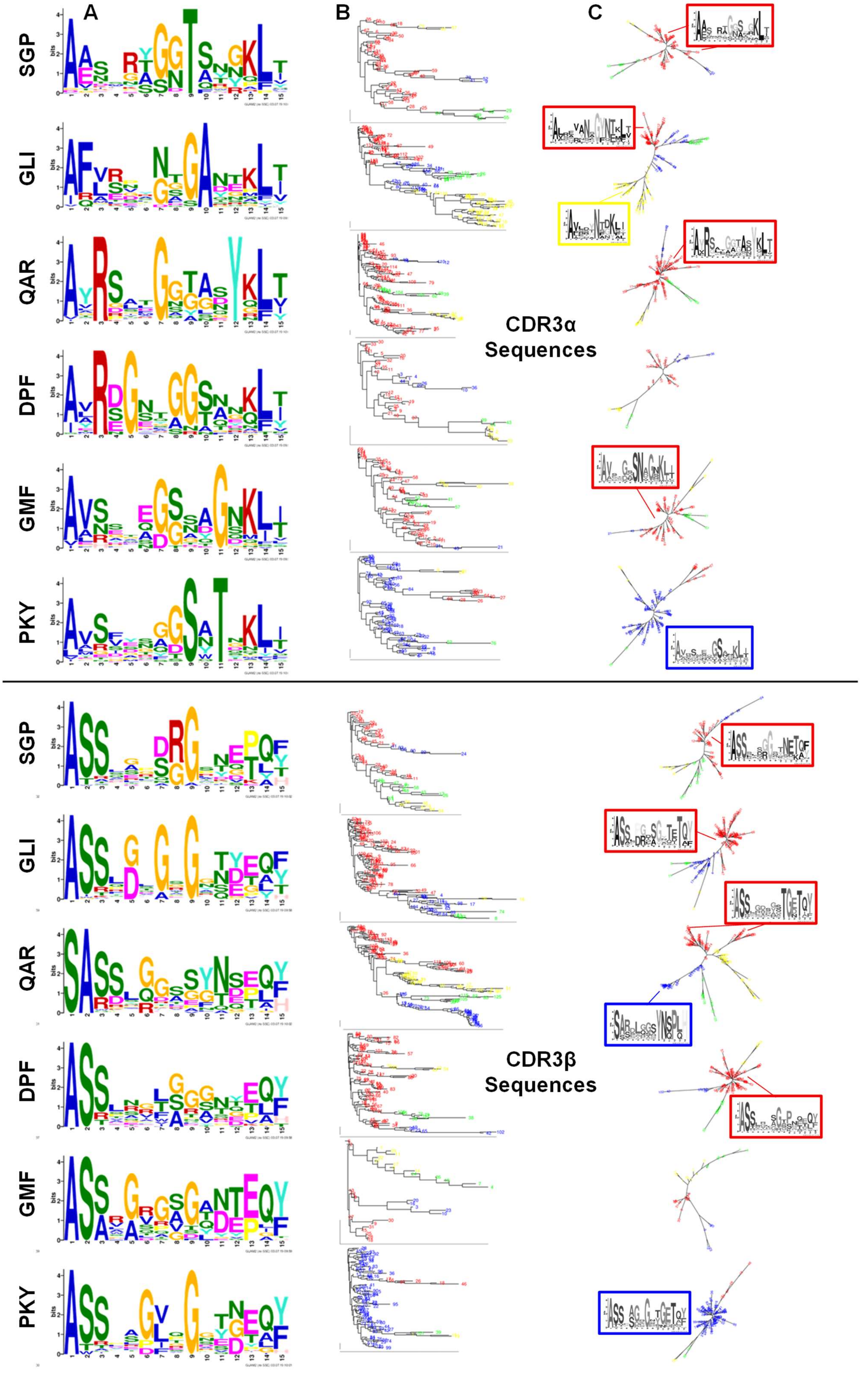
A
B
C
SGP
GLI
QAR
DPF
GMF
PKY
CDR3α Sequences
SGP
GLI
QAR
DPF
GMF
PKY
CDR3β Sequences

Figure S6. Analysis of CDR3 sequences to search for prominent motifs. Top pane shows results of CDR3α sequences, the bottom pane CDR3β sequences, Related to Figure 7. Column (**A**) GLAM2 (Gapped Alignment of Motifs) analysis performed on all CDR3 sequences in response to each epitope. The highest scoring motifs are shown for each epitope based on GLAM2 parameters detailed in methods (high iteration number and max motif length of 15 amino acids). Column (**B**) Phylogenetic analysis of CDR3 sequences in response to each epitope. Sequences were first aligned using MUSCLE and used to create a neighbour joining tree. The tree was then converted to a distance object which was cut into 4 subgroups and each tip coloured according to sub group membership (all code provided: https://github.com/ALGW71/ConservedEpitopesIAV). Column (C) Corresponding unrooted phylograms of CDR3 sequences shown in Supplementary Figure 13. GLAM2 analysis was performed on each subgroup (following methodology detailed in Chen et. al 2017) and the dominant high scoring motifs found by GLAM2 for large subgroups are displayed alongside. Colours correspond to subgroups.

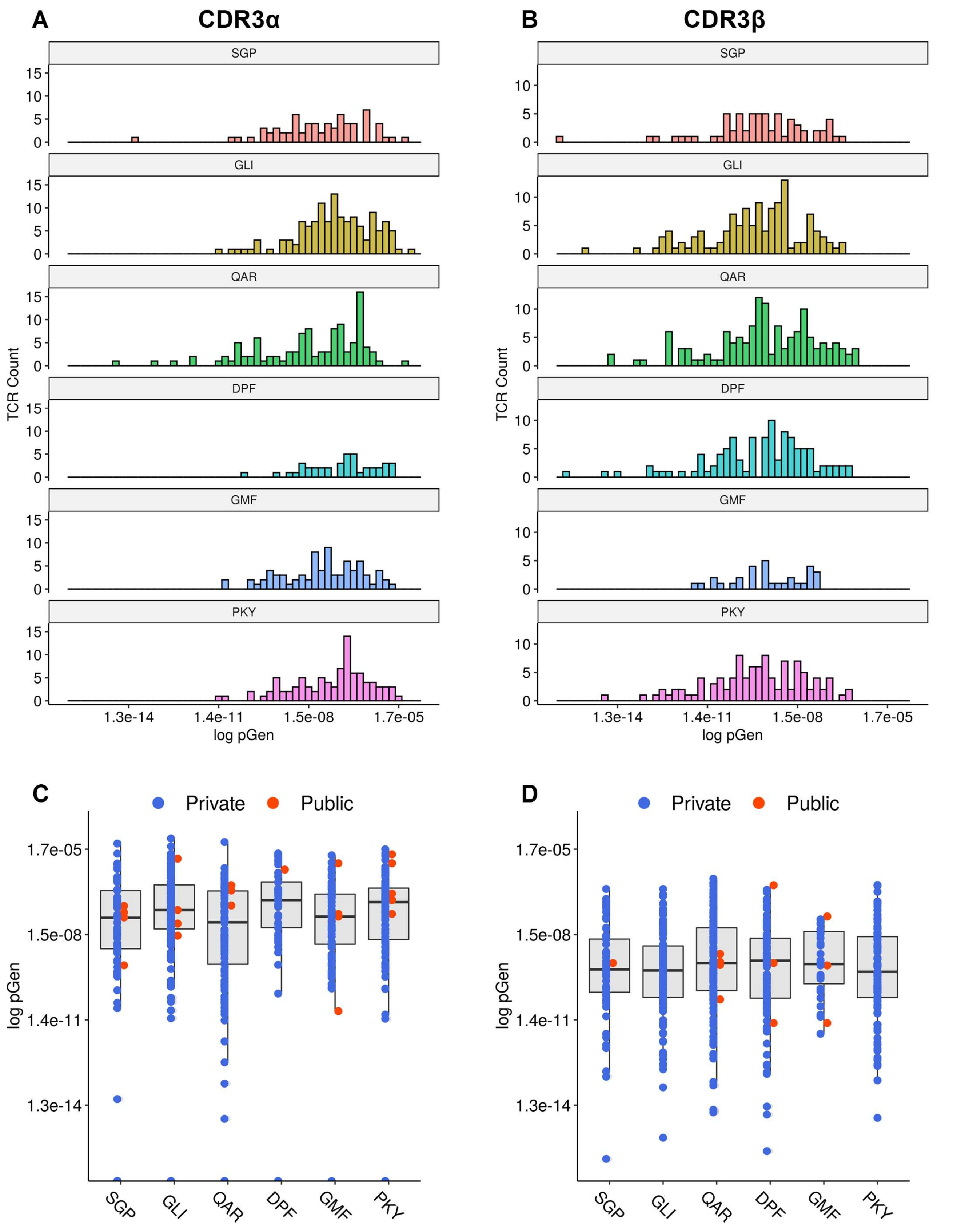

Figure S7. OLGA analysis of CDR3 Sequences, Related to Figure 7. Histograms showing CDR3α (**A**) and CDR3β (**B**) CDR3 generation probabilities (pGen) for each epitope. pGen values were calculated without VJ adjustment. Distributions distinguished by public (shared between more than one donor) or private sequences (detected in only one donor) are shown in for CDR3α (**C**) and CDR3β (**D**), with public sequences marked as orange dots.

DataS1. Detailed gene usage bar charts for each epitope and donor. Related to Figure 4. Bars are stacked by normalised percentage frequencies (percentage frequency in each donor, summed and normalised by the number of donors to allow for comparison). Colours correspond to each donor (see colour key).

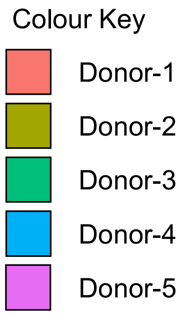

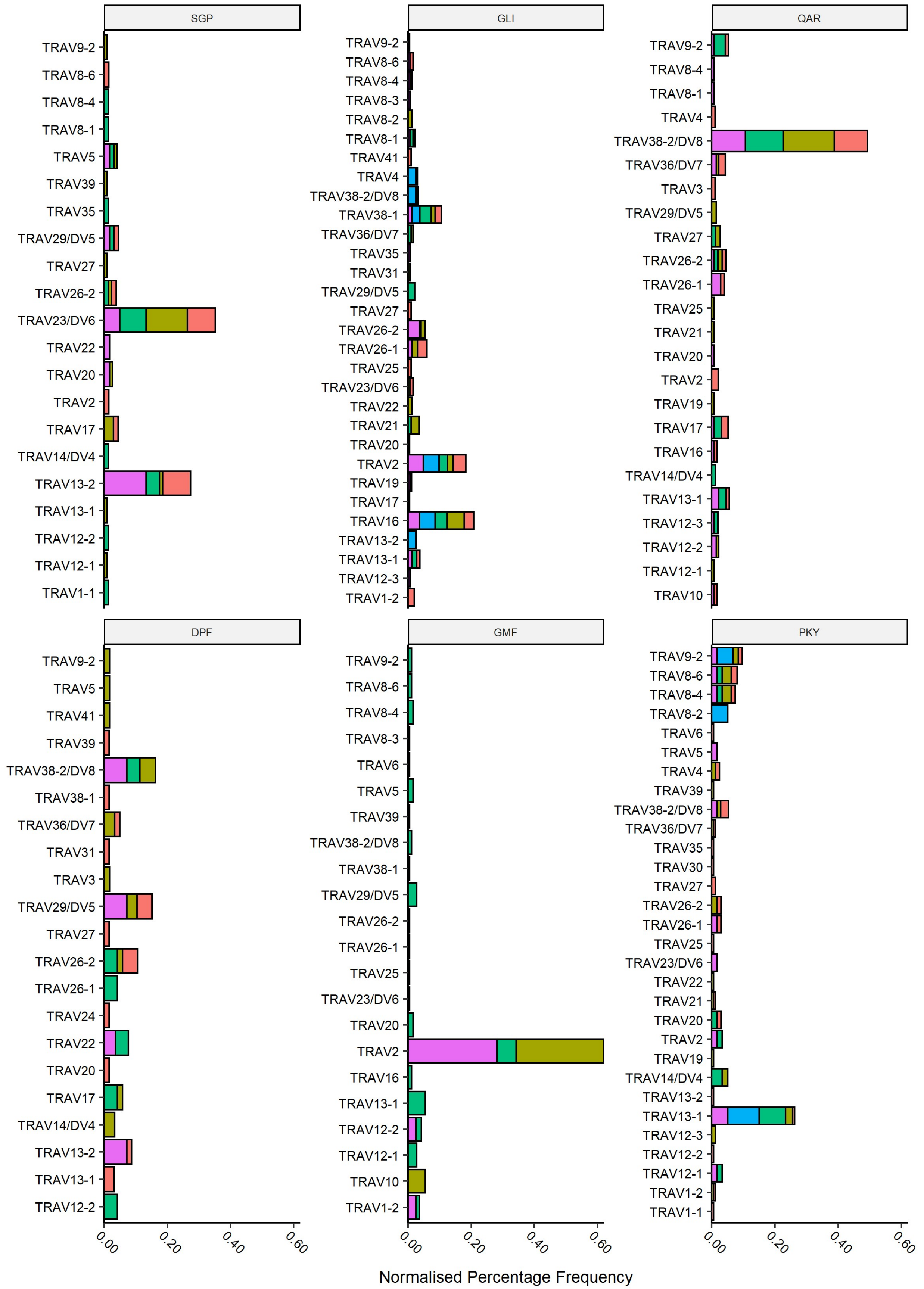

TRAV gene usage.

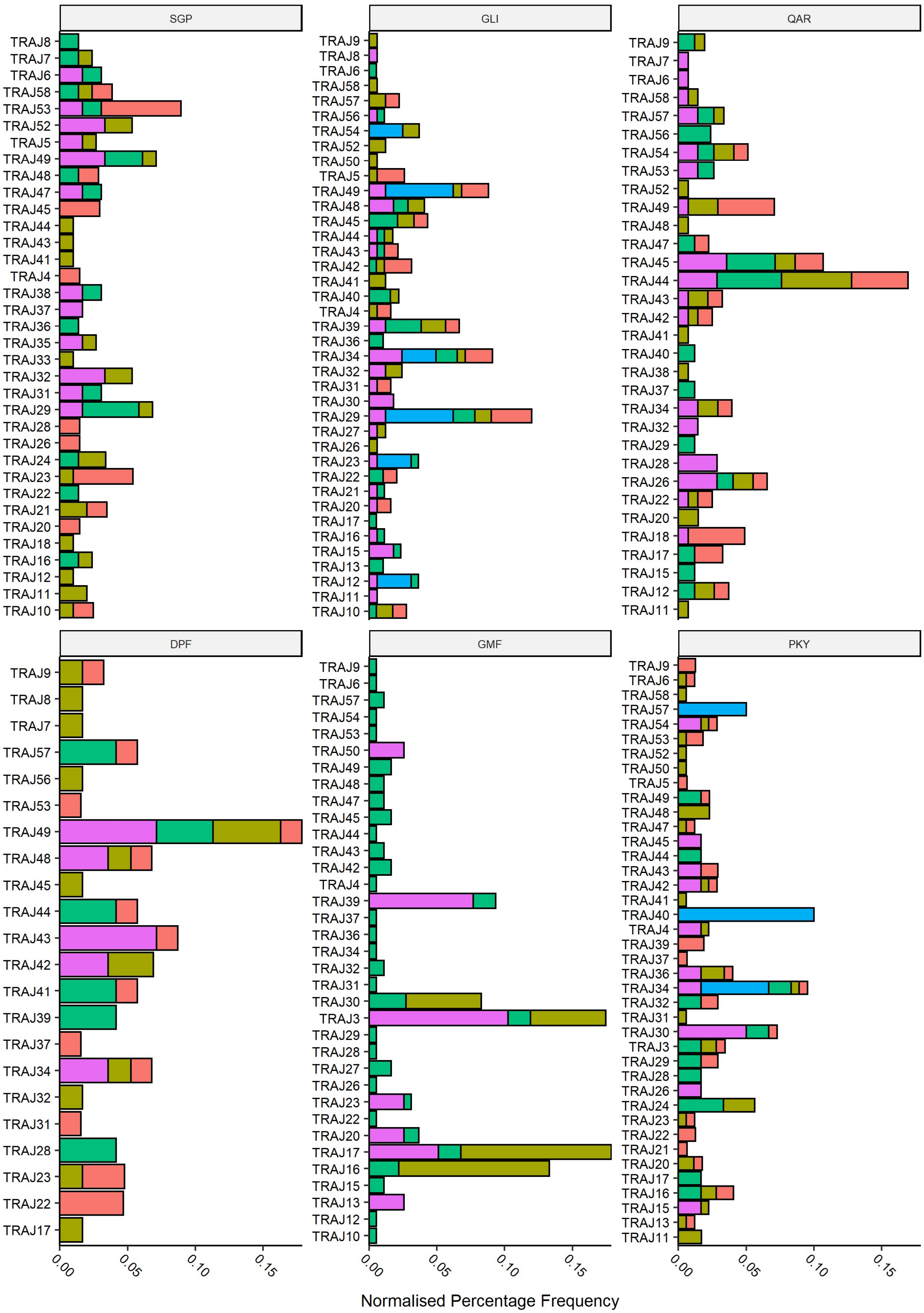

TRAJ gene usage.

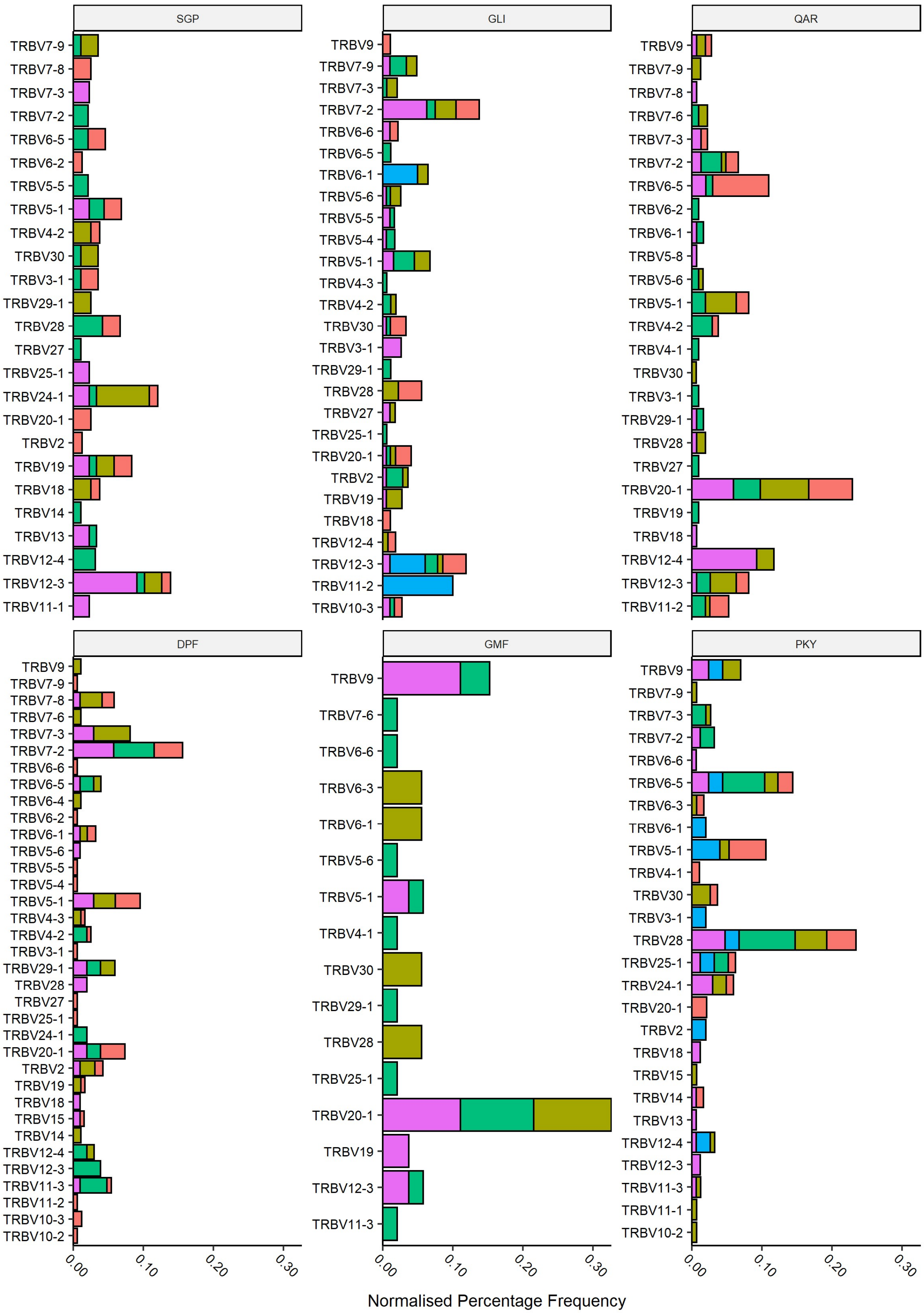

TRBV gene usage.

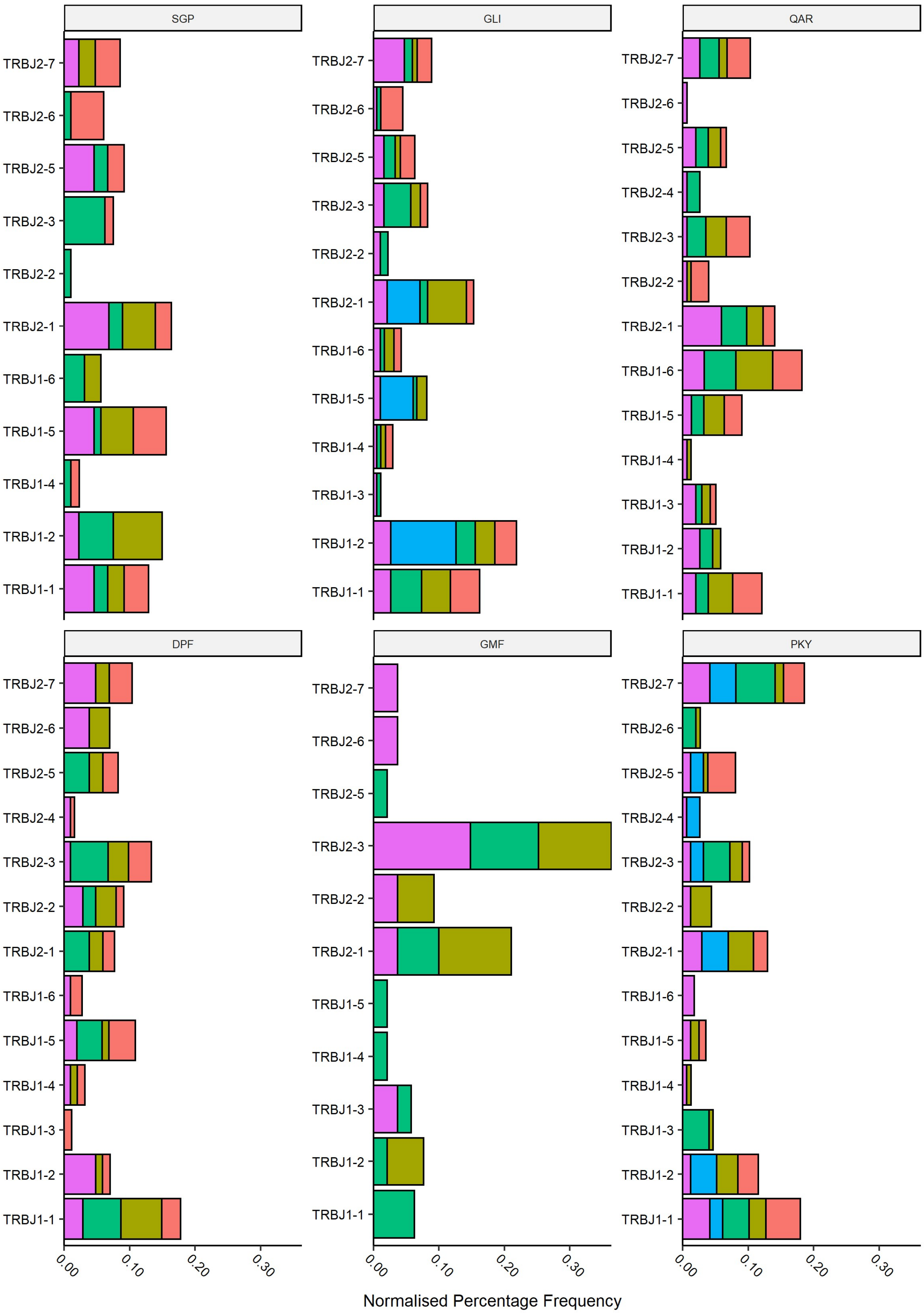

TRBJ gene usage.
