# Supplementary material for: CD4+ T Cells Recognize Conserved Influenza A Epitopes through Shared Patterns of V-Gene Usage and Complementary Biochemical Features
Source: Cell Rep. 2020 Jul 14;32(2):107885. doi: 10.1016/j.celrep.2020.107885 (PMC7370177; doi:10.1016/j.celrep.2020.107885)
Supplement: Document S2. Article plus Supplemental Information [file mmc8.pdf]

Cell Reports

Article

CD4$^+$ T Cells Recognize Conserved Influenza A Epitopes through Shared Patterns of V-Gene Usage and Complementary Biochemical Features

Graphical Abstract

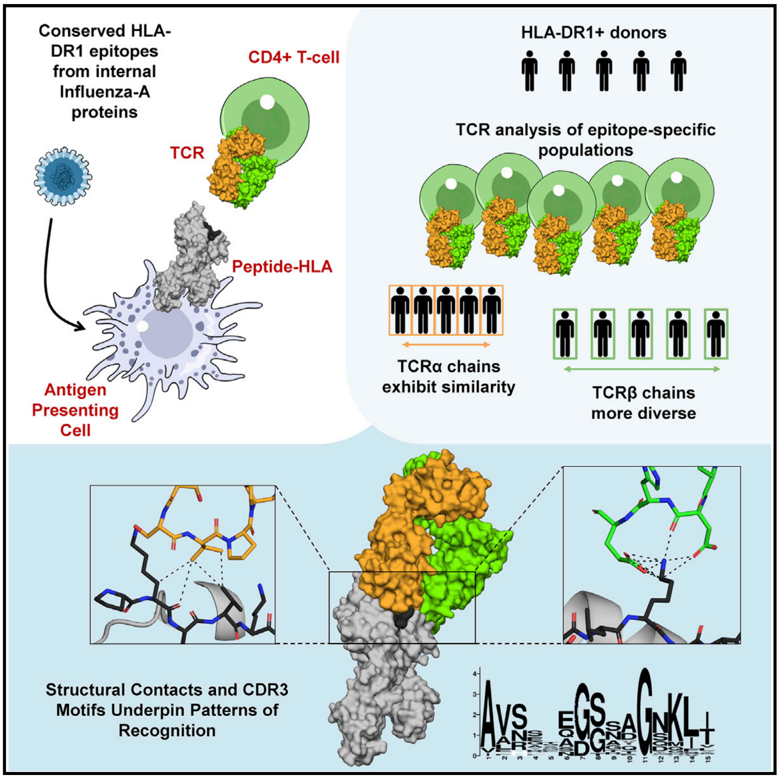

Highlights

- Conserved influenza epitopes are recognized in multiple HLA-DR1$^+$ donors
- Shared TRAV-gene usage underpins epitope-specific responses *in vitro*
- Structural analysis identifies biochemical features associated with V-gene selection
- CDR3 motifs and enrichments are evident in several shared "public" CDR3 sequences

Authors

Alexander Greenshields-Watson, Meriem Attaf, Bruce J. MacLachlan, ..., Andrew K. Sewell, Andrew J. Godkin, David K. Cole

Correspondence

godkinaj@cardiff.ac.uk (A.J.G.), coledk@cardiff.ac.uk (D.K.C.)

In Brief

CD4$^+$ T cells orchestrate protection from severe influenza. However, knowledge of epitopes and the molecular patterns associated with recognition across the population is lacking. Greenshields-Watson et al. identify several influenza epitopes from internal proteins and use them to explore the biochemical features that underpin CD4$^+$ T cell responses to influenza.

Greenshields-Watson et al., 2020, Cell Reports *32*, 107885
July 14, 2020 © 2020 The Author(s).
https://doi.org/10.1016/j.celrep.2020.107885

CellPress

Cell Reports

CellPress
OPEN ACCESS

Article

CD4$^+$ T Cells Recognize Conserved Influenza A Epitopes through Shared Patterns of V-Gene Usage and Complementary Biochemical Features

Alexander Greenshields-Watson,[1,5] Meriem Attaf,[1,5] Bruce J. MacLachlan,[1,4] Thomas Whalley,[1] Cristina Rius,[1] Aaron Wall,[1] Angharad Lloyd,[1] Hywel Hughes,[1] Kathryn E. Strange,[1,6,7] Georgina H. Mason,[1] Andrea J. Schauenburg,[1] Sarah L. Hulin-Curtis,[1] James Geary,[1] Yuan Chen,[1] Sarah N. Lauder,[1] Kathryn Smart,[1] Dhanasekaran Vijaykrishna,[4] Miguel L. Grau,[4] Mikhail Shugay,[2] Robert Andrews,[1] Garry Dolton,[1] Pierre J. Rizkallah,[1] Awen M. Gallimore,[1] Andrew K. Sewell,[1] Andrew J. Godkin,[1,3,5,*] and David K. Cole[1,5,8,9,*]

[1]Cardiff University, School of Medicine, Heath Park, Cardiff, UK
[2]Center of Life Sciences, Skolkovo Institute of Science and Technology, Moscow, Russia
[3]Department of Gastroenterology, Hepatology and Endoscopy, University Hospital of Wales, Cardiff, UK
[4]Monash Biomedicine Discovery Institute, 19 Innovation Walk, Clayton, Victoria 3800, Australia
[5]These authors contributed equally
[6]Present address: Anthony Nolan Research Institute, Royal Free Hospital, London, UK
[7]Present address: UCL Cancer Institute, Royal Free Campus, London, UK
[8]Present address: Immunocore, Milton Park, Abingdon, UK
[9]Lead Contact
*Correspondence: godkinaj@cardiff.ac.uk (A.J.G.), coledk@cardiff.ac.uk (D.K.C.)
https://doi.org/10.1016/j.celrep.2020.107885

SUMMARY

T cell recognition of peptides presented by human leukocyte antigens (HLAs) is mediated by the highly variable T cell receptor (TCR). Despite this built-in TCR variability, individuals can mount immune responses against viral epitopes by using identical or highly related TCRs expressed on CD8$^+$ T cells. Characterization of these TCRs has extended our understanding of the molecular mechanisms that govern the recognition of peptide-HLA. However, few examples exist for CD4$^+$ T cells. Here, we investigate CD4$^+$ T cell responses to the internal proteins of the influenza A virus that correlate with protective immunity. We identify five internal epitopes that are commonly recognized by CD4$^+$ T cells in five HLA-DR1$^+$ subjects and show conservation across viral strains and zoonotic reservoirs. TCR repertoire analysis demonstrates several shared gene usage biases underpinned by complementary biochemical features evident in a structural comparison. These epitopes are attractive targets for vaccination and other T cell therapies.

INTRODUCTION

T cells classically recognize short peptides presented by human leukocyte antigens (pHLAs) by the membrane-anchored heterodimeric $\alpha\beta$ T cell receptor (TCR). Each TCRα and TCRβ chain binds to the pHLA surface primarily using three-amino-acid loops termed complementarity determining regions (CDRs). The amino acid sequences of two of these loops, CDR1 and CDR2, are completely encoded within distinct variable (V) genes (TRAV and TRBV for α and β chains, respectively). The interactions they make with pHLA are termed germline contacts. CDR3 loops are the product of imprecise recombination between V, diversity (D; in β chain only), and junctional (J) genes. They are "hypervariable," with sequences that bear incomplete resemblance to parent V(D)J genes. It has been estimated that the theoretical TCR repertoire size is $>10^{15}$, with around 25 million unique TCRs thought to be expressed by an individual (Arstila et al., 1999; Sewell, 2012). Despite this built-in TCR variability, it has been established that individuals can mount immune responses against common epitopes by using identical or highly related TCRs expressed on CD8$^+$ T cells (Valkenburg et al., 2016; Venturi et al., 2008). Moreover, through inspection of TCR sequences selected against a given epitope, it is possible to cluster sequences that may bind their target by a similar molecular mechanism (Dash et al., 2017; Glanville et al., 2017) and, hence, explain strong biases in V-gene usage or CDR3 amino acid motifs. This information, in the context of multiple donors with a common HLA allele, enables the identification of TCR features that may underpin HLA-linked protective immunity in the population. These data aid the exploration of dominant TCR sequences that might be targeted by altered peptides (Cole et al., 2012) or may be more tolerant to point mutations arising from antigenic shift (Valkenburg et al., 2016). Although these shared "rules of engagement" have extended our understanding of the molecular mechanisms that govern TCR recognition of pHLA class I in the CD8$^+$ T cell system, for CD4$^+$ T cells, such information exists only for HIV infection (Benati et al.,

This is an open access article under the CC BY license (http://creativecommons.org/licenses/by/4.0/).

2016), celiac disease (Broughton et al., 2012; Petersen et al., 2016), and *Mycobacterium tuberculosis* infection (Glanville et al., 2017).

We investigated CD4$^+$ T cell responses to influenza A virus (IAV) as a model system with obvious relevance to human health. CD4$^+$ T cell responses to IAV, mediated through recognition of short peptides presented by HLA class II molecules on the surface of antigen-presenting cells, are essential to multiple antiviral processes that confer protection from severe symptomatic disease during IAV infection (Wilkinson et al., 2012). CD4$^+$ T cell responses can be directed toward any virion protein; yet, many studies into T and B cell immunity to IAV have focused on the external hemagglutinin (HA) and neuraminidase (NA) proteins. Indeed, existing molecular studies on CD4$^+$ T cells and IAV are limited to the "universal" HA epitope ($HA_{306-318}$, PKYVKQNTLKLAT), presented by HLA-DRA1*01:01/HLA-DRB1*04:01 (Glanville et al., 2017; Hennecke and Wiley, 2002) and HLA-DRA1*01:01/HLA-DRB1*01:01 (HLA-DR1) (Brawley and Concannon, 2002; Cameron et al., 2002; Cole et al., 2012; Hennecke et al., 2000). Although these external proteins are highly immunogenic, antigenic drift and shift limit their capacity to provide cross-protective immunity to novel viral strains. In contrast, the internal IAV proteins are more conserved (Heiny et al., 2007) and may better mediate cross-protective T cell responses (Chen et al., 2014; Sridhar et al., 2013; Wilkinson et al., 2012). Three of these proteins, matrix (M1), nucleoprotein (NP), and the catalytic subunit polymerase basic-1 (PB-1), exhibit consistent T cell immunogenicity (Hayward et al., 2015). Existing knowledge of cross-protective T cell responses to these proteins is heavily skewed toward CD8$^+$ T cells, specifically toward the M1-derived HLA-A2-presented $M1_{58-66}$-GIL epitope (Chen et al., 2017; Song et al., 2017; Valkenburg et al., 2016). To date, there are no structurally defined CD4$^+$ T cell epitopes from the internal proteins and no TCR repertoire data. Current knowledge of responses directed at internal IAV proteins has been derived from immunogenicity assays (DiPiazza et al., 2016; Wilkinson et al., 2012) or flow cytometry (Ge et al., 2010; Roti et al., 2008) involving long peptides or whole proteins, with a minority of work determining the minimal epitope (Chen et al., 2014).

We focused on the HLA class II molecule HLA-DR1 due to its high prevalence in the human population and the pre-existing molecular studies using this HLA type (Cole et al., 2012). Unbiased epitope mapping of the entire M1, NP, and PB-1 proteins revealed five IAV epitopes that elicited robust and reproducible responses across multiple HLA-DR1$^+$ subjects. We conducted a comprehensive analysis of the TCR repertoire by using next-generation sequencing (NGS) of HLA-DR1 multimer-isolated cells against all five epitopes. These analyses revealed biases in TRAV and, to a lesser extent, TRBV-gene usage shared across the multiple donors *in vitro*. Structural analysis demonstrated specific biochemical features and complementary electrostatics consistent with the highly focused gene usage patterns in response to certain epitopes. Thus, our findings exemplify how highly immunogenic CD4$^+$ T cell epitopes are underpinned by TCR recognition mechanisms shared across multiple HLA-DR1$^+$ individuals, particularly for TCRα chains.

RESULTS

Identification of Immunodominant Responses to Bona Fide HLA-DR1 Epitopes from the Internal IAV Proteins

To identify HLA-restricted epitopes within three internal IAV proteins (M1, NP, and PB-1; Table S1), we used an HLA-DR1-focused epitope mapping strategy in two HLA-DR1$^+$ donors by using overlapping peptide pools arranged into screening matrices (method described in Figure 1A; results in Figure S1). Fine mapping of the T cell responses using shorter 13/14-mer synthetic peptides based on *in silico* binding prediction (Table S2; Andreatta et al., 2015) was used to isolate five HLA-DR1-restricted epitopes for further analysis in four HLA-DR1$^+$ donors (Figure 1B): $M1_{17-30}$-SGP, $M1_{129-142}$-GLI, $M1_{208-222}$-QAR, $NP_{302-314}$-DPF, and $PB\text{-}1_{410-422}$-GMF. These five peptides elicited the most reproducible IFN-γ^+ CD4$^+$ T cell responses across all HLA-DR1$^+$ donors tested (Figures 1C and S2). An analysis of the literature showed that two of these epitopes had previously been identified in other studies: $M1_{129-142}$-GLI (Chen et al., 2014) and $M1_{17-30}$-SGP (Rothbard et al., 1988).

To further quantify and compare recognition of these epitopes, HLA multimers were used to stain 12- to 14-day peptide-expanded cultures in five HLA-DR1$^+$ donors (Figures 2A–2C; Figures S3 and S4). Staining was carried out alongside the universal epitope $HA_{306-318}$-PKY (Krieger et al., 1991), which served as a control for well-characterized and strong recognition. Side-by-side multimer staining and interferon γ (IFN-γ) ELISpot were also performed to confirm the functionality of responding populations (Figure S3). Robust epitope-specific responses were detected in all donors to the control epitope $HA_{306-318}$-PKY, with natural donor-specific variation in response magnitude (range, 6.9%–26.2% CD4$^+$). Of the five internal epitopes tested in five donors, 24 out of 25 possible responses were positive (defined as multimer staining/total CD4$^+$ T cells $\times$ 100 > 0.5%; donor-4 DPF was negative). The largest responses, whether measured in terms of size of CD4$^+$ T cell expansion, or of the median fluorescence intensity (MFI) of multimer positive cells, were consistently to $M1_{129-142}$-GLI followed closely by $HA_{306-318}$-PKY (Figures 2B and 2C).

In order to confirm that these epitopes were truly processed and presented in the context of viral infection, we infected DR1$^+$ (class 2 knockout [KO] C57BL/6) mice with X31, lab-adapted strain of IAV (H3N2) and measured *ex vivo* IFN-γ ELISpot responses (Figures 2D and 2E). Similar to our observed patterns in humans, responses to $M1_{17-30}$-SGP (mean, 22.0 SFC/1M splenocytes; positive response cutoff, 20 spot forming colonies (SFC)/1M; and double background) and $PB\text{-}1_{410-422}$-GMF (mean, 38.7 SFC) peptides were weakest, whereas $M1_{129-142}$-GLI (mean, 211.3 SFC) and $HA_{306-318}$-PKY peptides (mean, 298.7 SFC) had similar frequencies. However, the dominant responses in the HLA-DR1$^+$ mouse model were to the $NP_{302-314}$-DPF peptide, with mean SFC of 583.3. To control for HLA-DR1$^+$ T cell specificity, vaccination of HLA-DR1$^-$ mice (wild type [WT], C57BL/6 background; Figure 2E) was carried out alongside. The broad immunogenicity of these epitopes in multiple HLA-DR1$^+$ donors and transgenic HLA-DR1$^+$ mice identified them as interesting candidates for further analysis and investigation.

A

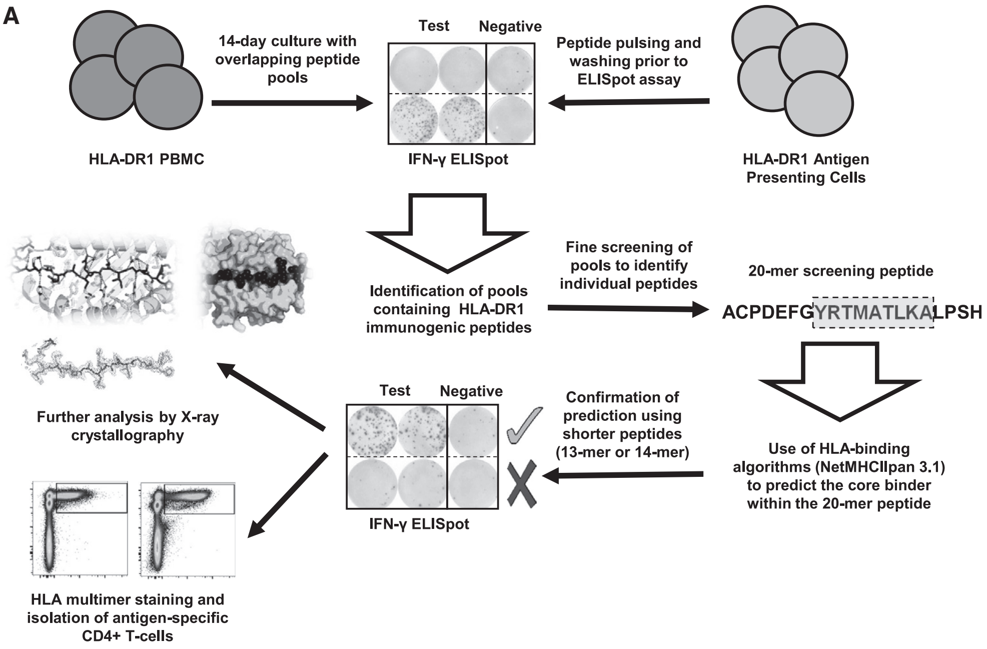

B

Protein	Peptide Name	Sequence	Peptide Sequence	NetMHCIIpan 3.1 Core DRB1*0101	P1	P4	P6	P9
M1	SGP	17-30	SGPLKAEIAQRLED	LKAEIAQRL	L	E	A	L
	GLI	129-142	GLIYNRMGAVTTEV	YNRMGAVTT	Y	M	A	T
	QAR	208-222	QARQMVQAMRTIGTHP	VQAMRTIGT	V	M	T	T
NP	DPF	302-314	DPFRLLQNSQVFS	FRLLQNSQV	F	L	N	V
PB-1	GMF	410-422	GMFNMLSTVLGVS	FNMLSTVLG	F	L	T	G

C

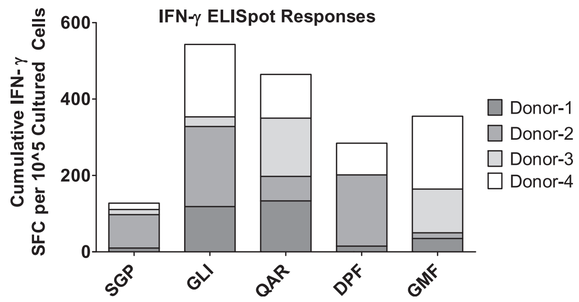

Figure 1. Identification of HLA-DR1 Epitopes from Three Internal Proteins of IAV

(A) Schematic representation of epitope mapping procedure. HLA-DR1^{+} donor peripheral blood mononuclear cells (PBMCs) cultured with influenza peptide pools were screened on IFN-γ ELISpot by using peptide-pulsed HLA-DR1^{+} antigen-presenting cells (APCs), followed by identification of immunogenic peptides and use of NetMHCIIpan to elucidate the 9-amino-acid core. Shorter peptides were tested on IFN-γ ELISpot, followed by further validation and analysis using HLA-multimer screens and X-ray crystallographic analysis of peptide-HLA structures.

(B) Table of identified HLA-DR1 epitopes and final peptide sequences used for further analysis. Anchor residues P1, P4, P6, and P9 are listed in far-right column, as indicated by NetMHCIIpan.

(C) Cumulative IFN-γ ELISpot responses to identified peptides in four HLA-DR1^{+} donors. Responses to each peptide per donor (mean of two replicates per donor) were stacked to give the cumulative response in terms of SFC per 10^5 cultured cells.

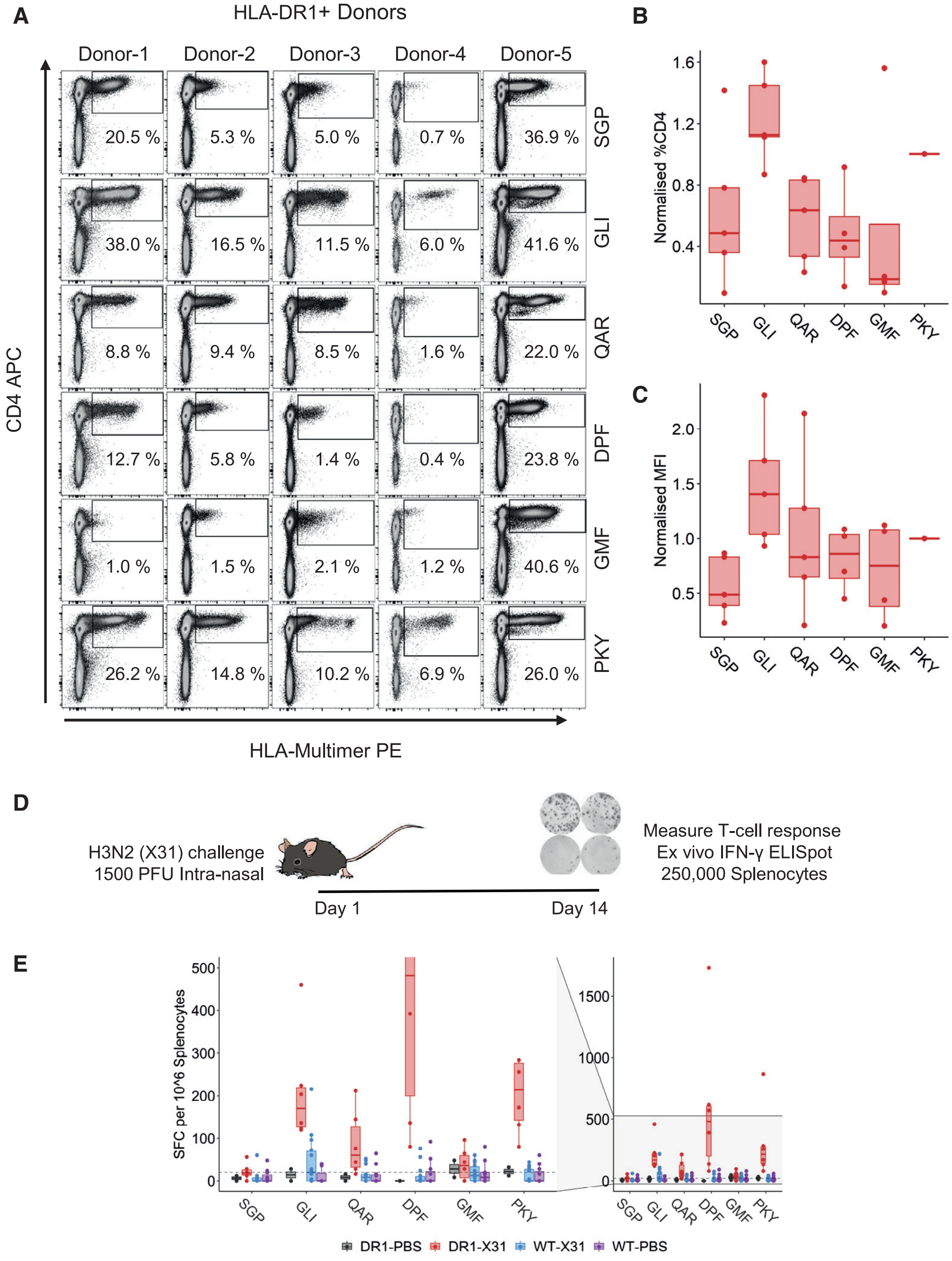

Figure 2. Quantification of Epitope-Specific CD4+ T Cell Populations in 5 HLA-DR1+ Donors *In Vitro* and an HLA-DR1+ *In Vivo* Mouse Model

(A) Epitope-specific HLA-multimer staining of PBMC lines cultured against HLA-DR1 epitopes. Columns correspond to each donor, and rows correspond to each epitope indicated on the right-hand side of each row. Populations are gated lymphocytes/live/CD3+. Percentages indicate HLA-multimer+ populations as a percentage of total lymphocytes/live/CD3+/CD4+ cells (gates were set based on fluorescence minus one [FMO] and irrelevant HLA-DR1 multimer controls).

(B) Boxplots of % CD4+ values all in donors. Values were normalized to corresponding % CD4+ values for the control HA epitope $HA_{306-318}$-PKY for each donor to account for culture variation.

(C) Corresponding median fluorescence intensity values of all donors normalized to $HA_{306-318}$-PKY MFI by donor. Boxplots show median and interquartile range; individual data points are shown as dots for each donor.

(D) Schematic detailing the experimental set up of an *in vivo* viral challenge model (DR1 X31 [n = 6], DR1 PBS [n = 2], WT X31 [n = 14], and WT PBS [n = 11]).

(E) *Ex vivo* IFN-γ ELISpot response data in response to peptides across four mouse groups. Full axis has been expanded on the left to show values close to the threshold for a positive response *ex vivo* (20 SFC) marked by the gray dashed line.

Internal Epitopes Exhibit Minor Differences in Absolute Conservation across Zoonotic Reservoirs

Despite the internal IAV proteins exhibiting higher levels of conservation relative to HA and NA, sequence variation is still present, particularly in the major zoonotic reservoirs of birds and swine that pose threats to the human population. We analyzed the sequence conservation of each internal epitope in over 17,000 avian sequences, 27,000 human sequences, and 8,000 swine sequences (Figure 3). The most conserved epitope was PB-$1_{410-422}$-GMF, with complete sequence conservation in 41,104 of 41,222 sequences (99.7%) from strains in humans, birds, and swine (Figures 3A–3F). $M1_{17-30}$-SGP showed the second highest conservation, at 97.9% in swine (Figure 3C) and 99.1% in human (Figure 3B), but 85.2% in avian strains (Figure 3A). Interestingly, both of these epitopes were found to be least immunogenic in both our mouse and human data (Figure 2). The more immunogenic epitopes *in vitro* and *in vivo*, namely, $M1_{129-142}$-GLI, $M1_{208-222}$-QAR, and $NP_{302-314}$-DPF, were less conserved, particularly in swine sequences (Figure 3C). $M1_{129-142}$-GLI, the most immunogenic epitope in humans, was conserved in 61.0% of human sequences (Figure 3B) but consistently contained at least one substitution in avian and swine strains (Figure 3H), resulting in conservation scores of less than 1% in each reservoir. Although $M1_{208-222}$-QAR and $NP_{302-314}$-DPF both had minimal numbers of identical sequences in human and swine (Figures 3B, 3C, 3E, and 3F), they were highly conserved in avian strains, at 86.7% and 98.1% sequence identity, respectively (Figures 3A and 3D). Due to the pandemic threat posed by H7N9, H9N2, and H5N1 we indicated these regions with red boxes on the heatmap in Figure 3D and demonstrated the conservation of $NP_{302-314}$-DPF in these highly relevant strains.

Despite the lack of absolute conservation (100% identity) in the most immunogenic epitopes, when we analyzed the mutational dissimilarity in terms of amino acid substitution (Figures 3G–3K), most mutations were one amino acid away (green dots). This was particularly striking for the highly immunogenic $M1_{129-142}$-GLI (Figure 3H) and $NP_{302-314}$-DPF (Figure 3J) sequences, in which most sequence variation in avian and human strains, respectively, can be attributed to a single mutation. Overall, this analysis confirmed that our panel of internal DR1 epitopes were highly conserved and relevant for further study, not only in humans but also in the major zoonotic reservoirs of bird and swine.

Epitope-Specific $CD4^+$ T Cell Populations Exhibit Skewed TRAV and Partial TRBV-Gene Usage Bias across HLA-$DR1^+$ Donors *In Vitro*

To gain insight into whether $CD4^+$ T cell responses were mediated by highly shared recognition mechanisms, comparable to those observed for immunodominant HLA class I epitopes (Chen et al., 2017; Song et al., 2017), we conducted TCR repertoire analysis using high-throughput sequencing in multiple HLA-$DR1^+$ donors. We isolated $multimer^+$ cells following *in vitro* peptide expansion (corresponding to plots shown in Figure 2) to obtain sufficient cell numbers for sequencing (Table S3). Inspection of the most frequently utilized genes, particularly TRAV and TRBV genes, may indicate if epitope recognition was dependent on highly specific germline-encoded contacts and specific binding mechanisms (Adams et al., 2016; Feng et al., 2007; Stewart-Jones et al., 2003), examples of which are limited in the context of HLA class II. Indeed, V-gene biases were seen in response to several epitopes shared across all donors (Figure 4; Data S1). This was most striking for PB-$1_{410-422}$-GMF for which more than 60% of TCRs utilized a single TRAV gene (Figure 4E; TRAV2, mean gene usage frequency = 62%; number of donors, n = 3) and predominantly recombined with one of four TRAJ genes (Figure 4E; chord diagram; TRAJ16, TRAJ17, TRAJ3, and TRAJ30). This was balanced by a smaller bias toward TRBV-gene usage (Figure 4K; TRBV20-1, mean = 32%, n = 3) paired largely with TRBJ2-3. The pattern of TRAV2 bias and its recombination with multiple TRAJ genes suggested a dominant TCR-α chain-mediated recognition mechanism that centered on TRAV-encoded germline residues, at either the CDR1, the CDR2, or the beginning of the CDR3.

The same trend of dominant TRAV gene bias, coupled with promiscuous TRAJ recombination, was observed in three other epitope-specific responses. $M1_{208-222}$-QAR specific repertoires were also directed to a single TRAV gene (Figure 4C; TRAV38-2/DVB8, mean = 50%, n = 4; mainly coupled with TRAJ44, TRAJ45, and TRAJ49) and to a much lesser extent a single TRBV gene (TRBV20-1, 23%, n = 4; Figure 4I). This was followed by $M1_{17-30}$-SGP that exhibited two TRAV gene biases (Figure 4A; TRAV13-2, mean = 27%, n = 4; TRAV23/DV6, mean = 35%, n = 4) but no obvious TRBV gene bias (Figure 4G). Responses to the control epitope $HA_{306-318}$-PKY showed a very similar TRAV and TRBV bias (Figure 4F; TRAV13-1, mean = 26%, n = 5; TRBV28, mean = 23%; Figure 4L). For $M1_{129-142}$-GLI, bias was distributed across three TRAV genes (TRAV2, mean = 18%; TRAV16, mean = 21%; and TRAV38-1, mean 11%, n = 5), compounded by weaker TRBV usage biases and more apparent donor diversity. The final epitope $NP_{302-314}$-DPF exhibited the strongest donor differences in V-gene bias, with broad usage of many TRAV and TRBV genes. Inspection of repertoires showed that two donors responded to $NP_{302-314}$-DPF with a single TCR sequence for both TCRα and TCRβ, and the remaining two donors had more diverse profiles (data not shown).

When looking at the overarching patterns in VJ-gene usage across all epitopes, the narrower usage of TRAV genes compared with TRBV genes in response to the same epitope was evident. This was tested through entropy (Figure 4M) and Kullback Leibler (KL) distance (Figure 4N; measured against the background repertoire). In response to all epitopes other than $HA_{306-318}$-PKY, TRAV usage bias was more focused than TRBV bias. Overall, our observations of V-gene bias were most likely to have roots in molecular features that involve germline contacts. We set out to investigate them through X-ray crystallography, exploring the contacts and biochemical features that were important for recognition.

Selection of Shared V Genes Is Governed by Germline-Mediated Peptide Interactions

To find structural mechanisms underpinning our observations of strong peptide-driven V-gene selection, we solved the structure of the F11 TCR in complex with HLA-DR1-PKY at a resolution of 1.91 Å (Table S5). The F11 TCR (Holland et al., 2018) has been

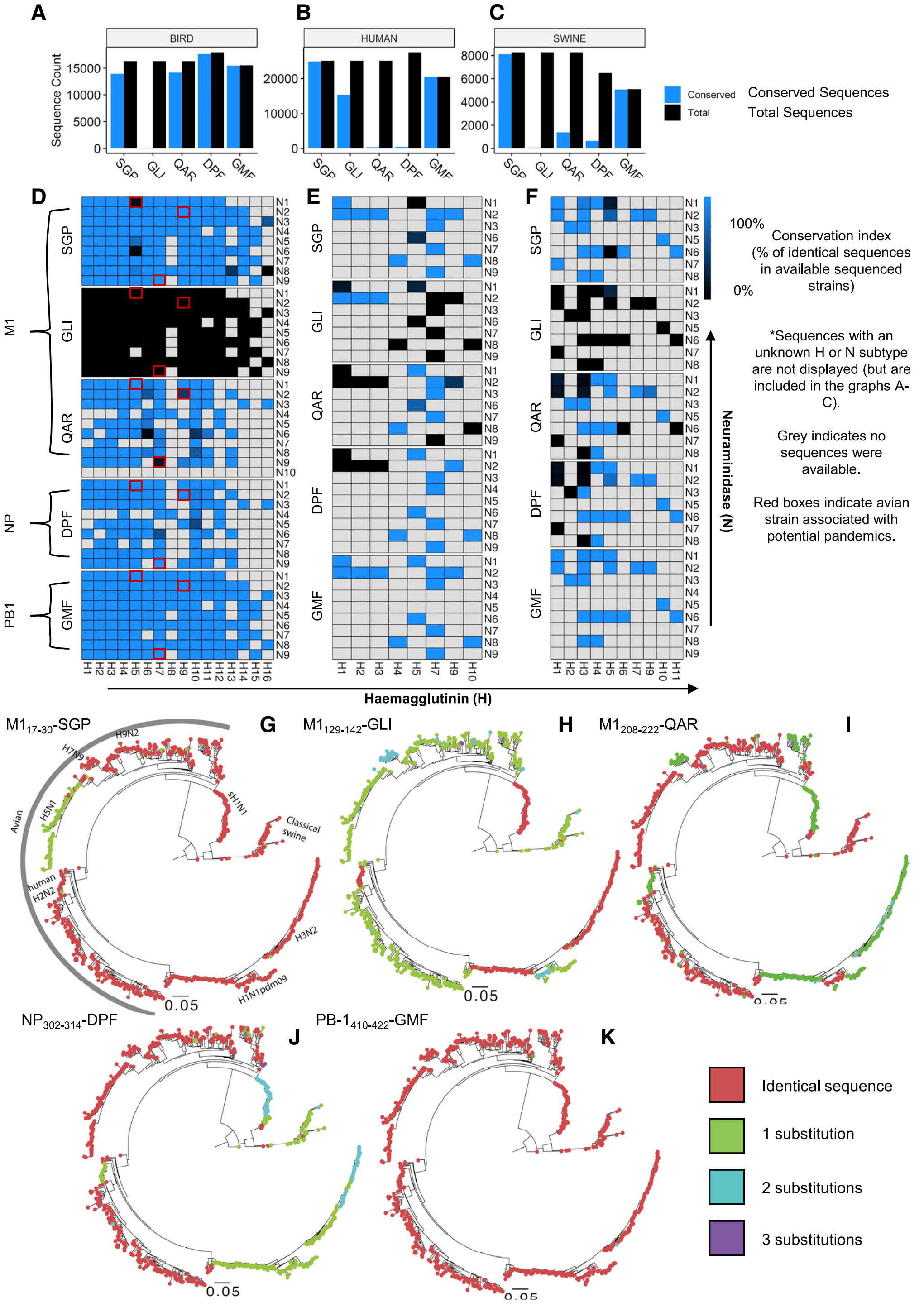

Figure 3. Analysis of HLA-DR1 Epitope Sequence Conservation in Human, Swine, and Avian Zoonotic Reservoirs

Sequence bar charts detailing the number of identical epitope sequences (blue) present in all sequenced IAV strains (black) in birds (A), humans (B), and swine (C). Corresponding breakdown of these sequences by hemagglutinin (H) and neuraminidase (N) subtypes shown as heatmaps for avian (D), human (E), and swine (F) sequences; the color scale indicates 100% conserved (blue) to not conserved (black). For each epitope, the details of substitutional divergence from the epitope sequences listed in Figure 1B are shown in the phylogenetic trees (G–K). Virus sequences with identical epitopes are marked in red, and the number of amino acid substitutions are color coded and indicated in the key. Major influenza virus lineages are shown in (G) and apply to the remaining phylogenies in (H–K).

CellPress
OPEN ACCESS

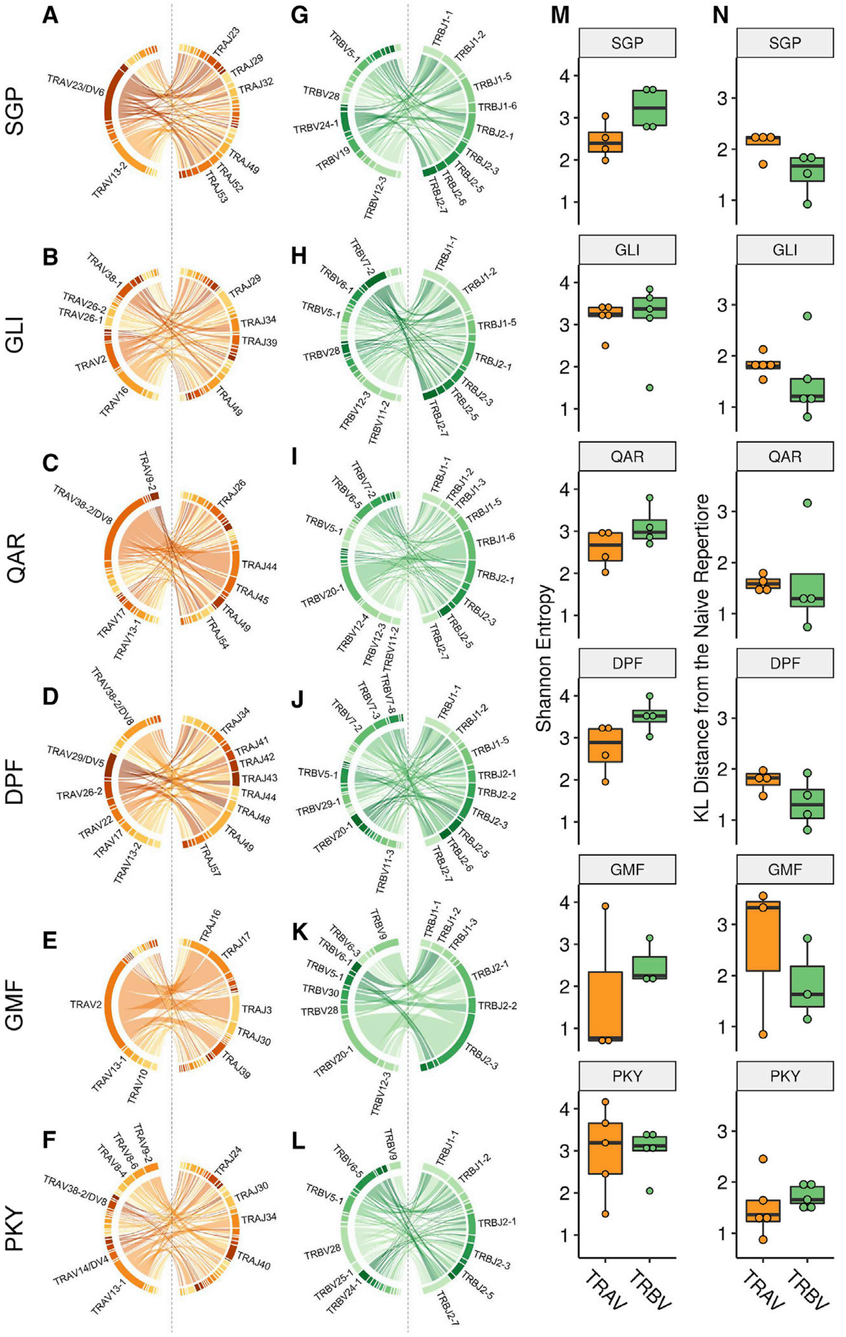

Figure 4. TCR VJ-Gene Usage Analysis of *In Vitro* CD4+ Responses to Conserved Epitopes

Percentage frequencies of V and J genes observed in response to a specific epitope, regardless of clonal expansion, were calculated for each donor. For each epitope, these values were summed and normalized to the number of donors (3–5 depending on epitope) to give the normalized percentage frequency (bar charts shown in Data S1).

(A–F) Circos plots showing TRAV- and TRAJ-gene usage cumulative percentage frequencies are shown. Chords that link between V and J genes, left and right of the dashed line, respectively, represent VJ pairing, with chord thickness proportional to the number of observed pairs. (A) TRAV usage for SGP, (B) TRAV usage for GLI, (C) TRAV usage for QAR, (D) TRAV usage for DFP, (E) TRAV usage for GMF, and (F) TRAV usage for PKY.

(G–L) Corresponding TRBV and TRBJ usage circos plots. Genes labeled on the outside of the circos were enriched above 5%; labels for those below 5% are not shown. (G) TRBV usage for SGP, (H) TRBV usage for GLI, (I) TRBV usage for QAR, (J) TRBV usage for DFP, (K) TRBV usage for GMF, and (L) TRBV usage for PKY.

(M) TRAV and TRBV Shannon entropy values for each epitope-specific response. Boxplots correspond to median entropy and interquartile range across all donors. Dots on top of each boxplot correspond to specific values for each donor. Higher entropy means the dataset is more diverse.

(N) TRAV and TRBV KL distance values from the naive repertoire (see STAR Methods for details on background V-gene usage). Greater distance values correspond to less diversity and narrower gene usage than would be expected from the normal repertoire.

shown to bind HLA-DR1-PKY with low μM affinity, comparable to the HA1.7 TCR (Cole et al., 2012). Both the F11 TCR and the HA1.7 TCR (Hennecke et al., 2000) share the use of the TRAV 8-4 gene (enriched in clonotyping; mean, 8%; n = 5), and yet, each has distinct CDR3 sequences, encoded by different TRAJ and TRBV genes (Table S6). Thus, we investigated whether the $HA_{306-318}$-PKY peptide interactions made by the TRAV8-4-encoded region of these two TCRs were conserved (Brawley and Concannon, 2002). Previous structural studies have described both situations in epitope recognition, notably the CDR3 editing hypothesis (Deng et al., 2012) and a study that argued for a reduced role of CDR1 and CDR2 in antigen recognition (Borg et al., 2005).

The F11 TCR bound to HLA-DR1-PKY with a canonical binding mode and exhibited similar crossing angle and overall binding mode to HA1.7 (Table S6). The total number of sub 4 Å contacts made by each TCR to HLA-DR1-PKY was similar (F11 = 103; HA1.7 = 104), as was the proportion of contacts contributed by each CDR loop to binding (Table S6). In each complex, neither the CDR2α nor CDR2β made any direct contacts with the peptide. Instead, CDR2β binding accounted for >30% of total TCR contacts and was likely a strong driver of HLA-DRα specificity (invariant compared with the polymorphic HLA-DRβ chain). In contrast, CDR1α contacted the peptide by the germline sequence SSVPPY encoded by TRAV8-4 in both TCRs, suggesting CDR1α may be a factor in driving the observed epitope specificity. CDR1α to peptide contacts were mediated by the Valα28 (SS**V**PPY) in the CDR1α loop for both complexes, each contacting the peptide at the P2Val side chain and the P-1Lys (Figures 5A and 5B). In each complex,

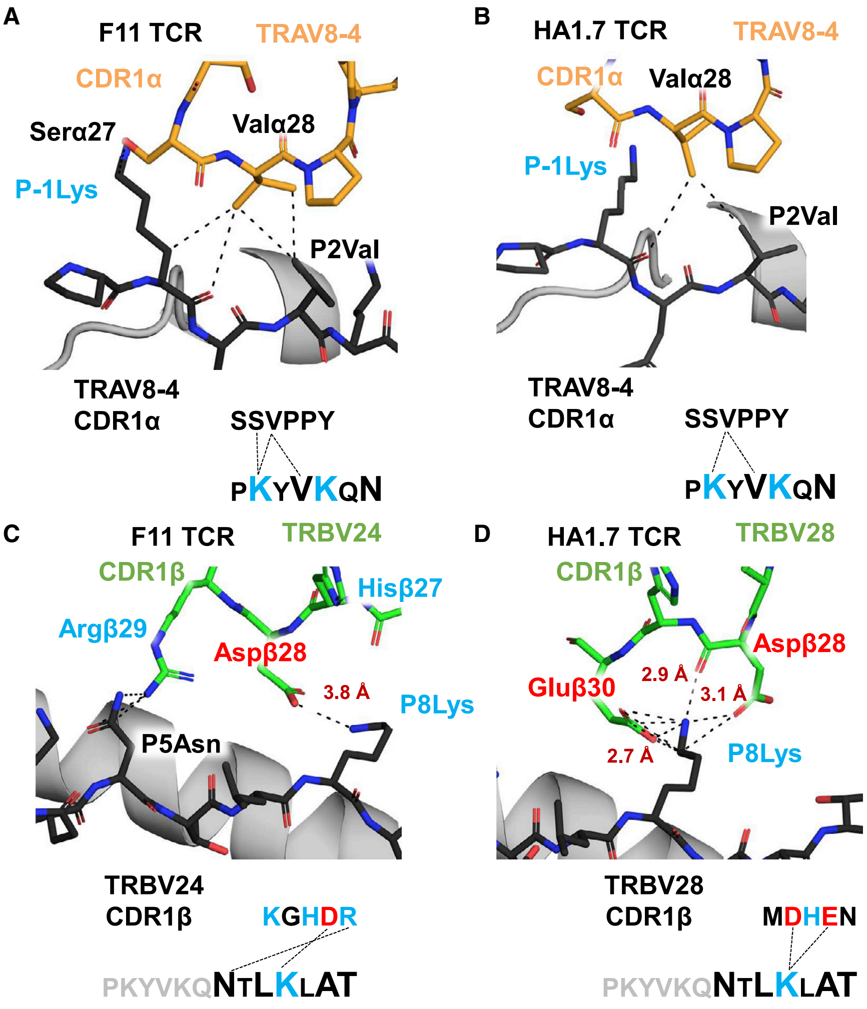

Figure 5. Structural Germline CDR1 Contacts to the Peptide May Drive V-Gene Usage Bias in TCRα and β Chains

CDR1α chain contacts made by F11 (A) and HA1.7 (B); any contacts within 4 Å are represented by dashed black lines. Bond distances of charged contacts and hydrogens bonds (identified by proteins, interfaces, structures, and assemblies [PISA]) are labeled in red text. Amino acid sequences of peptide and CDR1 are displayed below with upward-facing residues (not buried anchors) in larger font. CDR1β contacts for F11 (C) and HA1.7 (D) are represented in the same format.

the CDR1α loop was located directly above the N terminus of the PKY peptide and the same two peptide residues were in contact with the germline component of each TCR, regardless of differing CDR3α sequences, TRAJ genes, and TCRβ chains. Thus, both F11 and HA1.7, which share the enriched TRAV8-4, exhibit a highly similar overall binding to HLA-DR1-PKY and utilize the germline-encoded CDR1α for $HA_{306-318}$-PKY epitope recognition.

At the CDR1β, the less-enriched TRBV24-1 gene in F11 formed one salt bridge to P8Lys (Figure 5C) at 3.8 Å, in addition to three van der Waals interactions with the P5Asn. In contrast, the CDR1β loop of HA1.7 formed a strong triad of charge-based interactions with the P8Lys of the peptide (Figure 5D; Table S6). Three salt bridges (involving Gluβ30 and Aspβ28, 2.7–3.1 Å) and one hydrogen bond (backbone Aspβ28 carbonyl, 2.9 Å) were contributed by two CDR1β amino acids surrounding P8Lys, thus providing a strong peptide-specific interaction that is encoded in the germline sequence of the TRBV28 gene. TRBV28 was the most enriched gene in response to $HA_{306-318}$-PKY (mean = 23%, n = 5; Figure 4L), demonstrating how favorable CDR1 to peptide interactions might result in V-gene enrichments, as observed in our NGS data and previous findings by Hennecke et al. (2000) based on limited clonal sequencing data.

Germline TRAV-Encoded CDR3 Residues Do Not Contact the PKY Peptide

We next explored the CDR3α-to-peptide interactions and looked for germline-encoded residues that might explain the observed enrichment of the V-genes. The TRAV8-4 germline CDR3α component (*CAVS*…) did not form peptide contacts in either TCR complex (Figures 6A and 6B). Intriguingly, both TCRs utilized the same non-germline-encoded Gluα94 to make charge-charge peptide contacts to the flanking residue (P-1Lys), not the core of the epitope (Figures 6A and 6B). Analysis of our clonotyping data showed acidic residues to be exclusively selected for at this position (CDR3α residue 5; Figures 6A and 6B, sequence logo plots) across all TRAV8-4-encoded CDR3α sequences at the specific lengths used by F11 (4 sequences found in clonotyping data; Figure 6A) or HA1.7 (1 sequence found; Figure 6B). The fact that these residues were exclusively acidic (Asp or Glu) yet were not germline encoded (IMGT sequence database; Lefranc et al., 2015) suggest a charge-specific enrichment with a structurally defined role in recognition of the $HA_{306-318}$-PKY peptide.

The TRBV24 linked CDR3β of F11 positions two charged acidic residues near the P8Lys (Figure 6C), one germline encoded and the other the result of recombination. However, the germline-encoded (TRBV24, IMGT: CATS**D**L…) Aspβ93 was limited by orientation within the CDR3 loop, being positioned away from the P8Lys side chain and not forming any contact with the peptide. Instead, the non-germline-encoded Gluβ94 residue formed eight van der Waals contacts (3.2–4.0 Å) and positioned its carboxyl (-COO^-) side chain at 4.0 Å from the P8Lys amino group (-NH3 is colored red in Figure 6C). Overall, the CDR3β residues of F11 (both germline and hypervariable) formed more van der Waals contacts but an equivalent number of polar contacts to the peptide as the CDR1β (12 van der Waals

CellPress
OPEN ACCESS

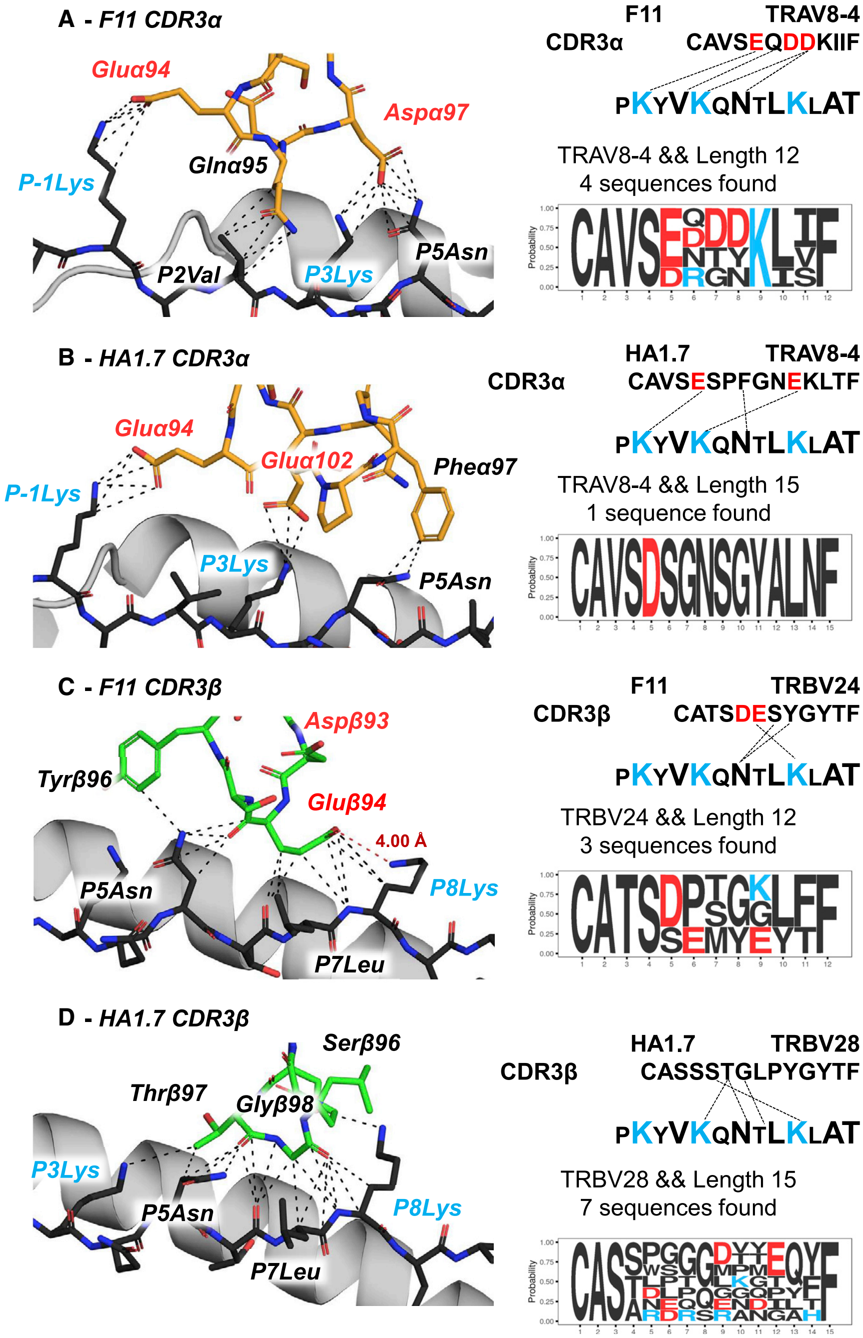

Figure 6. CDR3 Analysis Demonstrates that V-Gene Germline-Encoded CDR3 Residues Are Not in Contact with the Peptide

Combined structural and CDR-sequence analysis of CDR3α loop binding to the peptide by F11 (A) and HA1.7 (B), as well as CDR3β loop binding by F11 (C) and HA1.7 (D). In each panel, the left column depicts structural arrangement of each CDR loop interaction (CDR3α, orange; CDR3β, green) with the PKY peptide. All contacts within 4 Å are represented as dashed black lines. Residues are labeled according to side chain functional group charge (blue = basic, red = acidic, black = neutral). In each panel, the right column summarizes contacts made by each CDR loop (sequence-linker; top) and matching motif sequences encoded by the same V gene and of the same length (sequence-logo; bottom) within NGS data.

and 1 hydrogen bond, compared with 3 van der Waals and 1 salt bridge, respectively; Table S6), potentiating the argument that germline CDR1 contacts provide a substantial contribution to peptide specificity.

For the TCRβ chain of HA1.7, we observed that CDR1β binding to HLA-DR1-PKY by the highly enriched TRBV28 gene was mediated by a triad of charged or polar interactions (structural contacts in Figure 5D; gene usage in Figure 4L). This interaction may compensate for the absence of charged residues in CDR3β encoded by HA1.7. Furthermore, an analysis of all CDR3β sequences detected from clonotyping that were 15 residues in length and use TRBV28 were dominated by uncharged residues across the central sequence and a preference for acidic residues at position 6 and 9 in 2/7 and 3/7 sequences, respectively (sequence logo plot in Figure 6D). This finding would support the hypothesis that TRBV28 CDR1β interactions drive peptide specificity and allow for weaker interactions to dominate the CDR3β-peptide interface.

CDR3 Amino Acid Enrichments, Motifs, and Public Sequences Reflect Biochemical Complementarity between TCRs and the pHLA-II Surface

As part of our structural investigations, we solved pHLA crystal structures of three conserved IAV epitopes used for clonotypic analysis (Figure S5; HLA-DR1-SGP, HLA-DR1-QAR, and HLA-DR1-GMF; HLA-DR1-PKY from the F11 complex included for comparison). We generated peptide omit maps (Figure S5A), observed density maps (Figure S5B), and conducted atomic-B-factor analysis of core and flanking amino acids (Figure S5C) to confirm that observed core 9-mers matched those predicted by NetMHCIIPan 3.1 (Figure 1B).

Subsequently, inspection of the starkly contrasting electrostatic surfaces of these epitopes (Figure S5D) led us to look for sequence enrichments in the cognate CDR3 sequences that reflected simple biochemical complementarities (for example, opposite charge or shared hydrophobicity). At the simplest level, they included measurable enrichments in total CDR3 charge (Figure 7A) or CDR3 hydrophobicity (Figure 7B), which were clearly complementary to the surface electrostatics of pHLA

crystal structures (highly acidic surface of HLA-DR1-SGP and highly basic surface of HLA-DR1-PKY, Figure S5D), as well as the hydrophobic central surface of HLA-DR1-QAR. We then extended this analysis to look at CDR3 motifs, independent of sequence length, using GLAM2 (Figures 7C–7H; Figure S6). We performed GLAM2 analysis (Bailey et al., 2009) on all CDR3 sequences specific to each epitope and also split our sequences into closely related sub groups by using phylogenetic analysis (using MUSCLE; Edgar, 2004) to create neighbor-joining trees (Figure S6), following methods detailed in Chen et al. (2017). We found several of the highest scoring motifs (Figures 7C–7F) as well as positional enrichments (Figure 7G-H) obtained from GLAM2 and phylogenetic analyses of the entire pool of epitope-specific CDR3 sequences (33–132 sequences) to be present in the small number of public CDR3 sequences shared in multiple donors (28 sequences, tabulated in Figure 7 with J analysis detailed in Table S4). These protein motifs and positional enrichments were encoded both by germline nucleotides and by P- and N- nucleotide addition and deletion at the V(D)J junction. For $M1_{129-142}$-GLI, the NxGN motif (Figure 7C) originated from germline sequence of three of the four enriched TRAJ genes (TRAJ29, TRAJ39, and TRAJ49; Figure 4B) in response to this epitope. For $M1_{208-222}$-QAR (Figure 7E), the LxGxYN motif was partly hypervariable in origin (LxGx) and partly germline-encoded in the enriched TRBJ1-6 gene (YN, Figure 4I). The GxPxQ motif evident in CDR3β sequences in response to $NP_{302-314}$-DPF was exclusively hypervariable. Furthermore, this epitope elicited a public CDR3β sequence (CASSPGGSSYEQYF) in two donors with different TRBV genes, both interesting features of the response that showed the least V/J gene bias (Figures 4D and 4J). Although dominant motifs were not evident in public CDR3α sequences, specifically for $M1_{17-30}$-SGP (Figure 7G) and $HA_{306-318}$-PKY (Figure 7H), positional enrichments of single amino acids of like charge were apparent in multiple shared sequences. The enrichment of basic residues was exclusively germline in response to $M1_{17-30}$-SGP at the α chain (Figure 7G), whereas the enrichment for a central acidic residue in response to $HA_{306-318}$-PKY was present in both the hypervariable region and in germline TRAJ sequences.

Finally, we sought to determine whether the identified public TCRs were simply representative of highly favorable enrichments arising as a result of V(D)J recombination or were highly expanded in response to IAV infection from precursors that were less likely to arise in the natural repertoire. The latter scenario would suggest that public sequences represent important biochemical binding solutions (among a larger pool of possible solutions). In contrast, the former would indicate that such sequences are merely likely to be found in the naive repertoire and may not represent crucial biochemical binding solutions (which may otherwise come from CDR1 and CDR2 contacts or the partner chain). To do this, we used the optimized likelihood estimate of immunoglobulin amino-acid sequences (OLGA) tool (Sethna et al., 2019) to calculate generation probabilities (pGen) of each CDR3 (Figure S7). We calculated recombination probability distributions (Figures S7A and S7B) and then mapped onto these distributions the specific pGen values of "public" TCR sequences in our dataset (Figures S7C and S7D). Indeed, several of the public sequences displayed probabilities that fell toward the higher end of each distribution (particularly for the CDR3α, for which 75% of public sequences had a pGen value on or above the median and 35% in the upper quartile). This analysis indicated that most public CDR3α sequences we found were the result of highly probable recombination mechanisms, particularly for the TCRα chain. These observations were suggestive of highly biased TRAV gene selection linked to dominant CDR1α contacts with the peptide that may allow for instances of less stringent CDR3 selection at the TCRα chain (resulting in public TCRs with high pGen values). However, for the TCRβ chain, the origins of gene selection and CDR3 importance may be more complex (perhaps due to D segment insertion).

Ultimately, there exists a spectrum of interaction strength mediated by the combined effect of CDR1 and CDR3 contacts. Overall, we have deduced both sets of interactions from our data and demonstrated how such molecular relationships are the basis of shared $CD4^+$ T-cell-mediated immunity to conserved IAV epitopes.

DISCUSSION

TCR recognition of pathogen-derived peptides drives anti-viral $CD4^+$ T-cell-mediated immunity. For instance, in 2012, Wilkinson et al. (2012) found $CD4^+$ T cell responses specific to conserved influenza proteins correlated with heterosubtypic protection against pandemic IAV (Wilkinson et al., 2012). Yet, our knowledge of which peptides are most commonly recognized across the population and what facilitates this shared recognition is limited, especially in the context of HLA class II. This is particularly relevant to IAV, as nearly every adult is expected to have encountered the virus one or more times in their life, most likely starting in childhood (Munoz, 2002).

Here, we focused on $CD4^+$ T cell recognition of internal proteins from IAV in the context of HLA-DR1. We identified five HLA-DR1-restricted epitopes, derived from M1, NP, and PB-1, that elicited responses in multiple HLA-DR1$^+$ donors. The most immunogenic of these epitopes, $M1_{129-142}$-GLI, was able to stimulate cognate $CD4^+$ T cell populations in culture that were larger in magnitude and exhibited greater avidity for HLA multimers across all donors than the well-studied HLA class II influenza epitope $HA_{306-318}$-PKY. An analysis of cognate TCR repertoire populations *in vitro* exhibited biases in TRAV-gene usage and a comparatively reduced skewing in TRBV-gene usage and J genes. We searched for interactions that may help explain such biases by comparing the balance between germline CDR1 and hypervariable CDR3 contacts to $HA_{306-318}$-PKY in one novel and one published TCR-pHLA complex structure. This analysis highlighted CDR1-peptide interactions made by both the TCRα and TCRβ chains that were consistent with dominant gene usage biases we observed in TCR repertoire data from five HLA-DR1$^+$ donors. This was best exemplified by the observation that the enriched CDR1β sequence of TRBV28 was paired with a CDR3β that did not form any salt bridges, in addition to the consistent position and peptide contacts (P-1 and P2) made by the TRAV8-4-encoded CDR1α in both structures.

Several studies have already identified the importance of CDR1-peptide contacts in the recognition of HLA-class II

CellPress
OPEN ACCESS

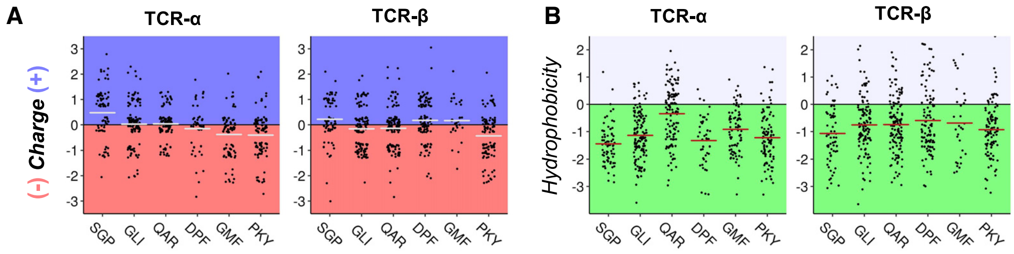

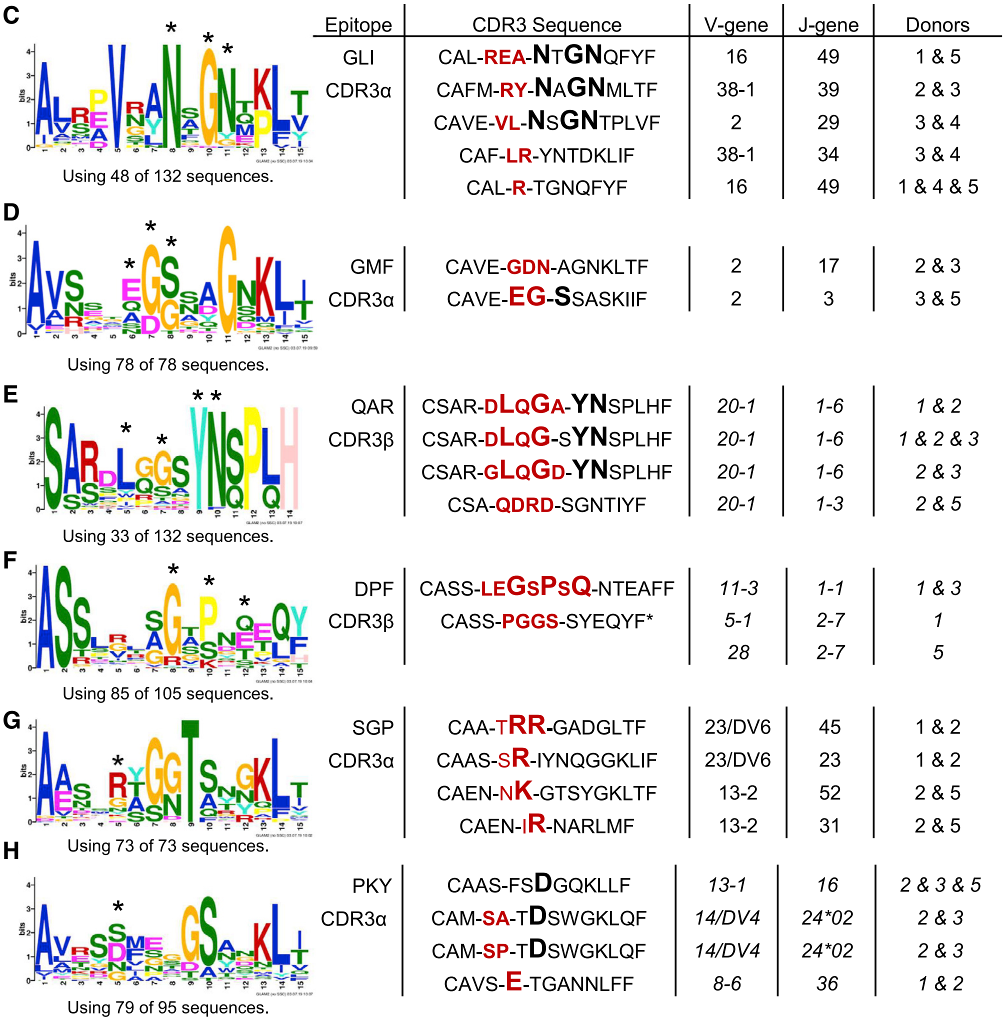

Epitope	CDR3 Sequence	V-gene	J-gene	Donors
GLI CDR3α	CAL-REA-NTGNQFYF	16	49	1 & 5
	CAFM-RY-NAGNMLTF	38-1	39	2 & 3
	CAVE-VL-NSGNTPLVF	2	29	3 & 4
	CAF-LR-YNTDKLIF	38-1	34	3 & 4
	CAL-R-TGNQFYF	16	49	1 & 4 & 5
GMF CDR3α	CAVE-GDN-AGNKLTF	2	17	2 & 3
	CAVE-EG-SSASKIIF	2	3	3 & 5
QAR CDR3β	CSAR-DLQGA-YNSPLHF	*20-1*	*1-6*	*1 & 2*
	CSAR-DLQG-SYNSPLHF	*20-1*	*1-6*	*1 & 2 & 3*
	CSAR-GLQGD-YNSPLHF	*20-1*	*1-6*	*2 & 3*
	CSA-QDRD-SGNTIYF	*20-1*	*1-3*	*2 & 5*
DPF CDR3β	CASS-LEGSPSQ-NTEAFF	*11-3*	*1-1*	*1 & 3*
	CASS-PGGS-SYEQYF*	*5-1*	*2-7*	*1*
		28	*2-7*	*5*
SGP CDR3α	CAA-TRR-GADGLTF	23/DV6	45	1 & 2
	CAAS-SR-IYNQGGKLIF	23/DV6	23	1 & 2
	CAEN-NK-GTSYGKLTF	13-2	52	2 & 5
	CAEN-IR-NARLMF	13-2	31	2 & 5
PKY CDR3α	CAAS-FSDGQKLLF	*13-1*	*16*	*2 & 3 & 5*
	CAM-SA-TDSWGKLQF	*14/DV4*	*24*02*	*2 & 3*
	CAM-SP-TDSWGKLQF	*14/DV4*	*24*02*	*2 & 3*
	CAVS-E-TGANNLFF	*8-6*	*36*	*1 & 2*

Figure 7. CDR3 Amino Acid Enrichments, Motifs, and Public Sequences Found in Sequences Responding to Conserved HLA-DR1 Epitopes
The central six amino acids of CDR3 sequences in response to each epitope were analyzed to quantify overall sequence charge (A) and hydrophobicity (B). Comparative CDR3 analysis between the output of GLAM2 conducted on either the whole set of CDR3 sequences specific to each epitope or a subgroup of sequences isolated from phylogenetic analysis detailed in Figure S6, respectively, with corresponding public CDR3 sequences (full details in Table S4). Shown are those epitopes for which high-scoring motifs (C–F) or positional enrichments (G and H) were observed. Below each motif are the number of sequences given to the GLAM2 algorithm resulting in the discovery of that motif. For (D) and (G), all CDR3α sequences specific to that epitope were given to the GLAM2 algorithm, whereas for (C), (E), (F), and (H), CDR3 sequences corresponding to a branch of the phylogenetic tree output from MUSCLE (Figure S7) were analyzed by GLAM2, resulting in discovery of the presented motif. In the public sequences, tabulated on the right, amino acids highlighted in bold indicate the motif or enrichment found in the corresponding output of GLAM2 are indicated on sequence logo plots with an asterisk. Amino acids encoded in either germline V or J genes are separated by a dash, and amino acids hypervariable in origin are colored red in bold typeset. Detailed V(D)J junctional analysis of all public CDR3 sequences are given in Table S4.

epitopes in the context of HIV (Galperin et al., 2018), celiac disease (Gunnarsen et al., 2017; Petersen et al., 2016), and $HA_{306-318}$-PKY (Brawley and Concannon, 2002) using crystallography, CDR or peptide mutagenesis, and kinetic analysis and/or CDR1α sequence randomization. Our work provides further evidence of this in the context of HLA class II and opens up new avenues of molecular investigation into CDR1 contacts made with both the peptide core (P1-P9) and flanking residues (P-1…P-n, P10…P+n). Future experiments involving mutagenesis of CDR1 residues and kinetic and crystallographic analyses will conclusively confirm which CDR1 residues are essential for epitope recognition and help quantify their effect on binding. There is still debate as to whether CDR1 and CDR2 contacts play a dominant role in peptide recognition (Borg et al., 2005; Deng et al., 2012). This undoubtably points to the fact that the TCR-pHLA interface is a dynamic interconnected network of interactions both with the peptide, HLA, and between the CDR loops themselves. As such, ascribing importance to the lesser studied CDR1 loops and quantifying their exact impact in the absence of knockon effects is complex but necessary to further our understanding, particularly in the context of HLA class II. Furthermore, single-cell cloning and expression of TCRs bearing dominant TRAV and TRBV genes in response to the epitopes characterized in this study will generate model systems to facilitate molecular investigations into the features of pHLA class II recognition.

Interestingly, the TCR gene biases we observed in our $CD4^+$ T cell responses to immunodominant epitopes may represent different mechanisms to those observed for $CD8^+$ T cell responses to immunodominant viral epitopes, including IAV (HLA-A*02:01-GILGFVFTL) (Chen et al., 2017; Song et al., 2017; Valkenburg et al., 2016) and EBV (HLA-A*02:01-GLCTLVAML) (Annels et al., 2000; Lim et al., 2000; Price et al., 2005), which are dominated by public TCR bias (memory T cells bearing near identical TCR sequences). These differences, which may demonstrate decreased reliance on publicity at the CDR3, could be related to the divergent nature of peptide presentation by HLA class I and HLA class II. For instance, the HLA class I binding groove is closed at each end, and presented peptides are generally forced to “kink” away from the HLA groove, forming a central bulge. This feature might act as a barrier for TCRs to make common HLA contacts and could limit the breadth of TCRs compatible with a unique peptide conformation. In contrast, the HLA class II binding groove is open at both ends and peptides are “pegged down” in four pockets along the bound nonamer (usually positions 1, 4, 6, and 9), leading to linear, “flatter” bound peptides. This flatter surface might enable a greater array of TCR binding modes and allow a larger degree of TCR-HLA interactions, which would be expected to reduce exclusivity in terms of TCRs with compatible antigen-binding sites for a given peptide. There is evidence suggesting that within the HLA class I system, the degree of TCR diversity can be altered depending on whether the peptide is relatively featureless or structurally unique (Cukalac et al., 2015). $CD8^+$ T cell responses are usually cytotoxic in nature, so the decision to activate may require a greater degree of accuracy to limit self-toxicity. In contrast, $CD4^+$ T cell responses usually provide help during an immune response, so there could be an advantage in activating a greater percentage of the $CD4^+$ T cell population, with less risk of self-reactivity leading to the direct destruction of healthy tissue. In summary, our findings exemplify how immunogenic $CD4^+$ T cell epitopes are underpinned by TCR recognition mechanisms shared across multiple HLA-$DR1^+$ individuals, extending our understanding of the mechanisms that control TCR selection against peptide-HLA class II.

STAR★METHODS

Detailed methods are provided in the online version of this paper and include the following:

- KEY RESOURCES TABLE
- RESOURCE AVAILABILITY
  - Lead Contact
  - Materials Availability
  - Data and Code Availability
- EXPERIMENTAL MODEL AND SUBJECT DETAILS
  - Primary Cell Culture
  - Cell Lines
  - Transgenic Mice
- METHOD DETAILS
  - Peptide libraries and pools
  - Sequence numbering of influenza proteins
  - IFN-γ ELISpot
  - Binding algorithm prediction
  - Production of soluble HLA-DR1 multimers
  - Preparation of HLA-Multimers
  - Human HLA-Multimer Staining
  - Conservation Analysis
  - TCR sequencing
  - Analysis and Visualization of TCR sequencing data in R
  - CDR3 Motif Analysis using GLAM2 and MUSCLE
  - OLGA
  - Crystallography
  - X-Ray Crystallographic Sample Preparation and Data Collection
- QUANTIFICATION AND STATISTICAL ANALYSIS
  - IFN-γ ELISpot assays
  - Flow Cytometry
  - TCR sequencing

SUPPLEMENTAL INFORMATION

Supplemental Information can be found online at https://doi.org/10.1016/j.celrep.2020.107885.

ACKNOWLEDGMENTS

The authors would like to thank Diamond Light Source for beam time (proposals MX10462-8, MX14843-6, and MX10462-9); the staff of beamlines I04 and I03 for assistance with crystal testing and data collection; as well as Catherine Reynolds, Rosemary Boyton, and Danny Altmann for supply of HLA-DR1 transgenics and help with initial studies. This work was funded by Wellcome and used equipment and reagents purchased by UK Biotechnology and Biological Sciences Research Council (grant BB/H001085/1). A.K.S. is a Wellcome Senior Investigator (WT100327MA). D.K.C. was a Wellcome Career Development Fellow (WT095767). A.G.-W. was a National Research Network

Wales-funded PhD student and is currently funded by Cancer Research Wales. B.J.M. was funded by a Cancer Research UK studentship and the Wales Cancer Research Centre. A.J.G., D.K.C., and A.M.G. are funded by a Wellcome Collaborator award. D.V. and M.L.G. are supported by contract HHSN272201400006C from the US National Institute of Allergy and Infectious Diseases (NIAID). A considerable quantity of this work was based on material previously published in a PhD thesis by A.G.-W. Additional crystallographic data, *in vivo* work, conservation analysis, bioinformatic analysis, and TCR sequencing data were included for this publication, as well as reanalysis and new visualization of all previous data

AUTHOR CONTRIBUTIONS

A.G.-W., M.A., and B.J.M. performed experimental work, data analysis, figure design, and preparation of the manuscript. A.W., H.H., C.R., A.L., K.E.S., G.H.M., A.J.S., and P.J.R. performed experimental work and/or data collection. P.J.R. and A.G.-W. analyzed and deposited structural data. A.G.-W., B.J.M., R.A., M.S., and T.W. wrote and checked code used for data analysis and figure design. S.L.H.-C., S.N.L., K.S., Y.C., and J.G. conducted *in vivo* experiments. D.V. and M.L.G. performed sequence conservation analysis. A.M.G., A.K.S., and G.D. provided material provision, experimental work (cell assays), figure design, and manuscript preparation. A.J.G. and D.K.C. funded and conceived the project and contributed to experimental design, figure design, and manuscript preparation.

DECLARATION OF INTERESTS

The authors declare no competing interests.

Received: March 17, 2019
Revised: January 20, 2020
Accepted: June 17, 2020
Published: July 14, 2020

SUPPORTING CITATIONS

The following references appear in the Supplemental Information: The following reference appears in the Supplemental Information: Giudicelli and Lefranc (2011).

REFERENCES

Adams, J.J., Narayanan, S., Birnbaum, M.E., Sidhu, S.S., Blevins, S.J., Gee, M.H., Sibener, L.V., Baker, B.M., Kranz, D.M., and Garcia, K.C. (2016). Structural interplay between germline interactions and adaptive recognition determines the bandwidth of TCR-peptide-MHC cross-reactivity. Nat. Immunol. *17*, 87–94.

Andreatta, M., Karosiene, E., Rasmussen, M., Stryhn, A., Buus, S., and Nielsen, M. (2015). Accurate pan-specific prediction of peptide-MHC class II binding affinity with improved binding core identification. Immunogenetics *67*, 641–650.

Annels, N.E., Callan, M.F.C., Tan, L., and Rickinson, A.B. (2000). Changing patterns of dominant TCR usage with maturation of an EBV-specific cytotoxic T cell response. J. Immunol. *165*, 4831–4841.

Arstila, T.P., Casrouge, A., Baron, V., Even, J., Kanellopoulos, J., and Kourilsky, P. (1999). A direct estimate of the human alphabeta T cell receptor diversity. Science *286*, 958–961.

Bagaev, D.V., Vroomans, R.M.A., Samir, J., Stervbo, U., Rius, C., Dolton, G., Greenshields-Watson, A., Attaf, M., Egorov, E.S., Zvyagin, I.V., et al. (2020). VDJdb in 2019: database extension, new analysis infrastructure and a T-cell receptor motif compendium. Nucleic Acids Res. *48*, D1057–D1062.

Bailey, T.L., Boden, M., Buske, F.A., Frith, M., Grant, C.E., Clementi, L., Ren, J., Li, W.W., and Noble, W.S. (2009). MEME SUITE: tools for motif discovery and searching. Nucleic Acids Res. *37*, W202–W208.

Baker, N.A., Sept, D., Joseph, S., Holst, M.J., and McCammon, J.A. (2001). Electrostatics of nanosystems: application to microtubules and the ribosome. Proc. Natl. Acad. Sci. USA *98*, 10037–10041.

Benati, D., Galperin, M., Lambotte, O., Gras, S., Lim, A., Mukhopadhyay, M., Nouël, A., Campbell, K.A., Lemercier, B., Claireaux, M., et al. (2016). Public T cell receptors confer high-avidity CD4 responses to HIV controllers. J. Clin. Invest. *126*, 2093–2108.

Bolotin, D.A., Poslavsky, S., Mitrophanov, I., Shugay, M., Mamedov, I.Z., Putintseva, E.V., and Chudakov, D.M. (2015). MiXCR: software for comprehensive adaptive immunity profiling. Nat. Methods *12*, 380–381.

Borg, N.A., Ely, L.K., Beddoe, T., Macdonald, W.A., Reid, H.H., Clements, C.S., Purcell, A.W., Kjer-Nielsen, L., Miles, J.J., Burrows, S.R., et al. (2005). The CDR3 regions of an immunodominant T cell receptor dictate the 'energetic landscape' of peptide-MHC recognition. Nat. Immunol. *6*, 171–180.

Brawley, J.V., and Concannon, P. (2002). Complementarity-determining region 1 sequence requirements drive limited V α usage in response to influenza hemagglutinin 307-319 peptide. J. Immunol. *168*, 3894–3901.

Britanova, O.V., Shugay, M., Merzlyak, E.M., Staroverov, D.B., Putintseva, E.V., Turchaninova, M.A., Mamedov, I.Z., Pogorelyy, M.V., Bolotin, D.A., Izraelson, M., et al. (2016). Dynamics of Individual T Cell Repertoires: From Cord Blood to Centenarians. J. Immunol. *196*, 5005–5013.

Broughton, S.E., Petersen, J., Theodossis, A., Scally, S.W., Loh, K.L., Thompson, A., van Bergen, J., Kooy-Winkelaar, Y., Henderson, K.N., Beddoe, T., et al. (2012). Biased T cell receptor usage directed against human leukocyte antigen DQ8-restricted gliadin peptides is associated with celiac disease. Immunity *37*, 611–621.

Bulek, A.M., Madura, F., Fuller, A., Holland, C.J., Schauenburg, A.J.A., Sewell, A.K., Rizkallah, P.J., and Cole, D.K. (2012). TCR/pMHC Optimized Protein crystallization Screen. J. Immunol. Methods *382*, 203–210.

Cameron, T.O., Cohen, G.B., Islam, S.A., and Stern, L.J. (2002). Examination of the highly diverse CD4(+) T-cell repertoire directed against an influenza peptide: a step towards TCR proteomics. Immunogenetics *54*, 611–620.

Chen, L., Zanker, D., Xiao, K., Wu, C., Zou, Q., and Chen, W. (2014). Immunodominant CD4+ T-cell responses to influenza A virus in healthy individuals focus on matrix 1 and nucleoprotein. J. Virol. *88*, 11760–11773.

Chen, G., Yang, X., Ko, A., Sun, X., Gao, M., Zhang, Y., Shi, A., Mariuzza, R.A., and Weng, N.-P. (2017). Sequence and Structural Analyses Reveal Distinct and Highly Diverse Human $CD8^+$ TCR Repertoires to Immunodominant Viral Antigens. Cell Rep. *19*, 569–583.

Cole, D.K., Gallagher, K., Lemercier, B., Holland, C.J., Junaid, S., Hindley, J.P., Wynn, K.K., Gostick, E., Sewell, A.K., Gallimore, A.M., et al. (2012). Modification of the carboxy-terminal flanking region of a universal influenza epitope alters $CD4^+$ T-cell repertoire selection. Nat. Commun. *3*, 665.

Collaborative Computational Project (1994). The CCP4 suite: programs for protein crystallography. Acta Crystallogr. D Biol. Crystallogr. *50*, 760–763.

Cukalac, T., Kan, W.T., Dash, P., Guan, J., Quinn, K.M., Gras, S., Thomas, P.G., and La Gruta, N.L. (2015). Paired TCR$\alpha\beta$ analysis of virus-specific CD8(+) T cells exposes diversity in a previously defined 'narrow' repertoire. Immunol. Cell Biol. *93*, 804–814.

Dash, P., Fiore-Gartland, A.J., Hertz, T., Wang, G.C., Sharma, S., Souquette, A., Crawford, J.C., Clemens, E.B., Nguyen, T.H.O., Kedzierska, K., et al. (2017). Quantifiable predictive features define epitope-specific T cell receptor repertoires. Nature *547*, 89–93.

Delano, W.L. (2002). The PyMOL Molecular Graphics System (Schrodinger).

Deng, L., Langley, R.J., Wang, Q., Topalian, S.L., and Mariuzza, R.A. (2012). Structural insights into the editing of germ-line-encoded interactions between T-cell receptor and MHC class II by Vα CDR3. Proc. Natl. Acad. Sci. USA *109*, 14960–14965.

DiPiazza, A., Richards, K.A., Knowlden, Z.A.G., Nayak, J.L., and Sant, A.J. (2016). The role of CD4 T cell memory in generating protective immunity to novel and potentially pandemic strains of influenza. Front. Immunol. *7*, 10.

Dolton, G., Tungatt, K., Lloyd, A., Bianchi, V., Theaker, S.M., Trimby, A., Holland, C.J., Donia, M., Godkin, A.J., Cole, D.K., et al. (2015). More tricks

with tetramers: a practical guide to staining T cells with peptide-MHC multimers. Immunology *146*, 11–22.

Edgar, R.C. (2004). MUSCLE: multiple sequence alignment with high accuracy and high throughput. Nucleic Acids Res. *32*, 1792–1797.

Emsley, P., and Cowtan, K. (2004). Coot: model-building tools for molecular graphics. Acta Crystallogr. D Biol. Crystallogr. *60*, 2126–2132.

Feng, D., Bond, C.J., Ely, L.K., Maynard, J., and Garcia, K.C. (2007). Structural evidence for a germline-encoded T cell receptor-major histocompatibility complex interaction "codon.". Nat. Immunol. *8*, 975–983.

Galperin, M., Farenc, C., Mukhopadhyay, M., Jayasinghe, D., Decroos, A., Benati, D., Tan, L.L., Ciacchi, L., Reid, H.H., Rossjohn, J., et al. (2018). $CD4^+$ T cell-mediated HLA class II cross-restriction in HIV controllers. Sci. Immunol. *3*, eaat0687.

Ge, X., Tan, V., Bollyky, P.L., Standifer, N.E., James, E.A., and Kwok, W.W. (2010). Assessment of seasonal influenza A virus-specific CD4 T-cell responses to 2009 pandemic H1N1 swine-origin influenza A virus. J. Virol. *84*, 3312–3319.

Giudicelli, V., and Lefranc, M.P. (2011). IMGT/junctionanalysis: IMGT Standardized Analysis of the V-J and V-D-J Junctions of the Rearranged Immunoglobulins (IG) and T Cell Receptors (TR). Cold Spring Harb. Protoc. *2011*, 716–725.

Glanville, J., Huang, H., Nau, A., Hatton, O., Wagar, L.E., Rubelt, F., Ji, X., Han, A., Krams, S.M., Pettus, C., et al. (2017). Identifying specificity groups in the T cell receptor repertoire. Nature *547*, 94–98.

Gu, Z., Gu, L., Eils, R., Schlesner, M., and Brors, B. (2014). circlize Implements and enhances circular visualization in R. Bioinformatics *30*, 2811–2812.

Gunnarsen, K.S., Høydahl, L.S., Risnes, L.F., Dahal-Koirala, S., Neumann, R.S., Bergseng, E., Frigstad, T., Frick, R., du Pré, M.F., Dalhus, B., et al. (2017). A TCRα framework-centered codon shapes a biased T cell repertoire through direct MHC and CDR3β interactions. JCI Insight *2*, e95193.

Gupta, N.T., Vander Heiden, J.A., Uduman, M., Gadala-Maria, D., Yaari, G., and Kleinstein, S.H. (2015). Change-O: a toolkit for analyzing large-scale B cell immunoglobulin repertoire sequencing data. Bioinformatics *31*, 3356–3358.

Hayward, A.C., Wang, L., Goonetilleke, N., Fragaszy, E.B., Bermingham, A., Copas, A., Dukes, O., Millett, E.R.C., Nazareth, I., Nguyen-Van-Tam, J.S., et al. (2015). Natural T cell-mediated protection against seasonal and pandemic influenza: Results of the flu watch cohort study. Am. J. Respir. Crit. Care Med. *191*, 1422–1431.

Heiny, A.T., Miotto, O., Srinivasan, K.N., Khan, A.M., Zhang, G.L., Brusic, V., Tan, T.W., and August, J.T. (2007). Evolutionarily conserved protein sequences of influenza a viruses, avian and human, as vaccine targets. PLoS One *2*, e1190.

Hennecke, J., and Wiley, D.C. (2002). Structure of a complex of the human alpha/beta T cell receptor (TCR) HA1.7, influenza hemagglutinin peptide, and major histocompatibility complex class II molecule, HLA-DR4 (DRA*0101 and DRB1*0401): insight into TCR cross-restriction and alloreactivity. J. Exp. Med. *195*, 571–581.

Hennecke, J., Carfi, A., and Wiley, D.C. (2000). Structure of a covalently stabilized complex of a human alphabeta T-cell receptor, influenza HA peptide and MHC class II molecule, HLA-DR1. EMBO J. *19*, 5611–5624.

Holland, C.J., MacLachlan, B.J., Bianchi, V., Hesketh, S.J., Morgan, R., Vickery, O., Bulek, A.M., Fuller, A., Godkin, A., Sewell, A.K., et al. (2018). In silico and structural analyses demonstrate that intrinsic protein motions guide T cell receptor complementarity determining region loop flexibility. Front. Immunol. *9*, 674.

Krieger, J.I., Karr, R.W., Grey, H.M., Yu, W.Y., O'Sullivan, D., Batovsky, L., Zheng, Z.L., Colón, S.M., Gaeta, F.C.A., Sidney, J., et al. (1991). Single amino acid changes in DR and antigen define residues critical for peptide-MHC binding and T cell recognition. J. Immunol. *146*, 2331–2340.

Lefranc, M.P., Giudicelli, V., Duroux, P., Jabado-Michaloud, J., Folch, G., Aouinti, S., Carillon, E., Duvergey, H., Houles, A., Paysan-Lafosse, T., et al. (2015). IMGT®, the international ImMunoGeneTics information system® 25 years on. Nucleic Acids Res. *43*, D413–D422.

Lim, A., Trautmann, L., Peyrat, M.A., Couedel, C., Davodeau, F., Romagné, F., Kourilsky, P., and Bonneville, M. (2000). Frequent contribution of T cell clonotypes with public TCR features to the chronic response against a dominant EBV-derived epitope: application to direct detection of their molecular imprint on the human peripheral T cell repertoire. J. Immunol. *165*, 2001–2011.

McCoy, A.J., Grosse-Kunstleve, R.W., Adams, P.D., Winn, M.D., Storoni, L.C., and Read, R.J. (2007). Phaser crystallographic software. J. Appl. Cryst. *40*, 658–674.

Munoz, F.M. (2002). The impact of influenza in children. Semin. Pediatr. Infect. Dis. *13*, 72–78.

Murshudov, G.N., Skubák, P., Lebedev, A.A., Pannu, N.S., Steiner, R.A., Nicholls, R.A., Winn, M.D., Long, F., and Vagin, A.A. (2011). REFMAC5 for the refinement of macromolecular crystal structures. Acta Crystallogr. D Biol. Crystallogr. *67*, 355–367.

Nazarov, V.I., Pogorelyy, M.V., Komech, E.A., Zvyagin, I.V., Bolotin, D.A., Shugay, M., Chudakov, D.M., Lebedev, Y.B., and Mamedov, I.Z. (2015). tcR: an R package for T cell receptor repertoire advanced data analysis. BMC Bioinformatics *16*, 175.

Paradis, E., and Schliep, K. (2019). ape 5.0: an environment for modern phylogenetics and evolutionary analyses in R. Bioinformatics *35*, 526–528.

Petersen, J., Kooy-Winkelaar, Y., Loh, K.L., Tran, M., van Bergen, J., Koning, F., Rossjohn, J., and Reid, H.H. (2016). Diverse T Cell Receptor Gene Usage in HLA-DQ8-Associated Celiac Disease Converges into a Consensus Binding Solution. Structure *24*, 1643–1657.

Price, D.A., Brenchley, J.M., Ruff, L.E., Betts, M.R., Hill, B.J., Roederer, M., Koup, R.A., Migueles, S.A., Gostick, E., Wooldridge, L., et al. (2005). Avidity for antigen shapes clonal dominance in CD8+ T cell populations specific for persistent DNA viruses. J. Exp. Med. *202*, 1349–1361.

Rius, C., Attaf, M., Tungatt, K., Bianchi, V., Legut, M., Bovay, A., Donia, M., Thor Straten, P., Peakman, M., Svane, I.M., et al. (2018). Peptide-MHC Class I Tetramers Can Fail To Detect Relevant Functional T Cell Clonotypes and Underestimate Antigen-Reactive T Cell Populations. J. Immunol. *200*, 2263–2279.

Rothbard, J.B., Lechler, R.I., Howland, K., Bal, V., Eckels, D.D., Sekaly, R., Long, E.O., Taylor, W.R., and Lamb, J.R. (1988). Structural model of HLA-DR1 restricted T cell antigen recognition. Cell *52*, 515–523.

Roti, M., Yang, J., Berger, D., Huston, L., James, E.A., and Kwok, W.W. (2008). Healthy human subjects have CD4+ T cells directed against H5N1 influenza virus. J. Immunol. *180*, 1758–1768.

Sethna, Z., Elhanati, Y., Callan, C.G., Walczak, A.M., and Mora, T. (2019). OLGA: fast computation of generation probabilities of B- and T-cell receptor amino acid sequences and motifs. Bioinformatics *35*, 2974–2981.

Sewell, A.K. (2012). Why must T cells be cross-reactive? Nat. Rev. Immunol. *12*, 669–677.

Shugay, M., Bagaev, D.V., Turchaninova, M.A., Bolotin, D.A., Britanova, O.V., Putintseva, E.V., Pogorelyy, M.V., Nazarov, V.I., Zvyagin, I.V., Kirgizova, V.I., et al. (2015). VDJtools: Unifying Post-analysis of T Cell Receptor Repertoires. PLoS Comput. Biol. *11*, e1004503.

Song, I., Gil, A., Mishra, R., Ghersi, D., Selin, L.K., and Stern, L.J. (2017). Broad TCR repertoire and diverse structural solutions for recognition of an immunodominant $CD8^+$ T cell epitope. Nat. Struct. Mol. Biol. *24*, 395–406.

Sridhar, S., Begom, S., Bermingham, A., Hoschler, K., Adamson, W., Carman, W., Bean, T., Barclay, W., Deeks, J.J., and Lalvani, A. (2013). Cellular immune correlates of protection against symptomatic pandemic influenza. Nat. Med. *19*, 1305–1312.

Stamatakis, A. (2014). RAxML version 8: a tool for phylogenetic analysis and post-analysis of large phylogenies. Bioinformatics *30*, 1312–1313.

Stewart-Jones, G.B.E., McMichael, A.J., Bell, J.I., Stuart, D.I., and Jones, E.Y. (2003). A structural basis for immunodominant human T cell receptor recognition. Nat. Immunol. *4*, 657–663.

Theaker, S.M., Rius, C., Greenshields-Watson, A., Lloyd, A., Trimby, A., Fuller, A., Miles, J.J., Cole, D.K., Peakman, M., Sewell, A.K., and Dolton, G. (2016). T-cell libraries allow simple parallel generation of multiple peptide-specific human T-cell clones. J. Immunol. Methods *430*, 43–50.

Tungatt, K., Bianchi, V., Crowther, M.D., Powell, W.E., Schauenburg, A.J., Trimby, A., Donia, M., Miles, J.J., Holland, C.J., Cole, D.K., et al. (2015). Antibody stabilization of peptide-MHC multimers reveals functional T cells bearing extremely low-affinity TCRs. J. Immunol. *194*, 463–474.

Valkenburg, S.A., Josephs, T.M., Clemens, E.B., Grant, E.J., Nguyen, T.H.O., Wang, G.C., Price, D.A., Miller, A., Tong, S.Y.C., Thomas, P.G., et al. (2016). Molecular basis for universal HLA-A*0201-restricted $CD8^+$ T-cell immunity against influenza viruses. Proc. Natl. Acad. Sci. USA *113*, 4440–4445.

Venturi, V., Chin, H.Y., Asher, T.E., Ladell, K., Scheinberg, P., Bornstein, E., van Bockel, D., Kelleher, A.D., Douek, D.C., Price, D.A., and Davenport, M.P. (2008). TCR beta-chain sharing in human CD8+ T cell responses to cytomegalovirus and EBV. J. Immunol. *181*, 7853–7862.

Wagih, O. (2017). ggseqlogo: a versatile R package for drawing sequence logos. Bioinformatics *33*, 3645–3647.

Wickham, H. (2014). Tidy Data. J. Stat. Softw. *59*, 1–23.

Wilkinson, T.M., Li, C.K.F., Chui, C.S.C., Huang, A.K.Y., Perkins, M., Liebner, J.C., Lambkin-Williams, R., Gilbert, A., Oxford, J., Nicholas, B., et al. (2012). Preexisting influenza-specific CD4+ T cells correlate with disease protection against influenza challenge in humans. Nat. Med. *18*, 274–280.

Winn, M.D., Ballard, C.C., Cowtan, K.D., Dodson, E.J., Emsley, P., Evans, P.R., Keegan, R.M., Krissinel, E.B., Leslie, A.G.W., McCoy, A., et al. (2011). Overview of the CCP4 suite and current developments. Acta Crystallogr. D Biol. Crystallogr. *67*, 235–242.

STAR★METHODS

KEY RESOURCES TABLE

REAGENT or RESOURCE	SOURCE	IDENTIFIER
Antibodies		
PE-Dextramer Backbone	Immundex	Cat#DX01-PE
Anti-human CD4 allophycocyanin (clone M-T466)	Miltenyi Biotec	Cat#130-113-250
Anti-human CD8 allophycocyanin-vio770 (clone BW135/80)	Miltenyi Biotec	Cat#170-081-073
Anti-human CD3 peridinin chlorophyll protein (clone BW264/56)	Miltenyi Biotec	Cat#130-113-131
Anti-human CD19 pacific blue (clone HIB19)	Biolegend	Cat#302224
Anti-human CD14 pacific blue (clone M5E2)	Biolegend	Cat#301815
LIVE/DEAD Fixable Dead Stain Vivid	Life Technologies	Cat#L34955
Anti-PE “Boost” (clone PE001)	Biolegend	Cat#408108
Anti-HLA-DR (clone L243)	Biolegend	Cat#307602
Bacterial and Virus Strains		
Rosetta (DE3) competent BL21 *E. coli* cells	Novogen	Cat#70954
X31 Influenza A virus	Laboratory of Ian Humphreys	A/HongKong/X31
Biological Samples		
Peripheral blood of local HLA-DR1+ individuals	Local Donors	N/A
Chemicals, Peptides, and Recombinant Proteins		
Peptide Pools Matrix 1	GL Biochem (Shanghai) Ltd	A/Wilson-Smith/1933(H1N1)
Peptide Pools Nucleoprotein	GL Biochem (Shanghai) Ltd	A/Ck/HK/96.1/02 (H5N1)
Peptide Pools Polymerase basic-1	GL Biochem (Shanghai) Ltd	A/Puerto Rico/8/1934(H1N1)
SGP-M1$_{17-30}$ peptide (High purity)	Peptide Protein Research Ltd	A/Wilson-Smith/1933(H1N1)
GLI-M1$_{129-142}$ peptide (High purity)	Peptide Protein Research Ltd	A/Wilson-Smith/1933(H1N1)
QAR-M1$_{208-222}$ peptide (High purity)	Peptide Protein Research Ltd	A/Wilson-Smith/1933(H1N1)
DPF-NP$_{273-285}$ peptide (High purity)	Peptide Protein Research Ltd	A/Ck/HK/96.1/02 (H5N1)
GMF-PB1$_{410-422}$ peptide (High purity)	Peptide Protein Research Ltd	A/Puerto Rico/8/1934(H1N1)
PKY-HA$_{306-318}$ peptide (High purity)	Peptide Protein Research Ltd	A/Texas/1/1977 (H3N2)
Phytohaemagglutinin-L (PHA)	Sigma	Cat#11249738001
Dastatinib (protein kinase inhibitor)	Axon Medchem	Cat#BMS354825
Cellkines (rhIL-2)	Helvetica Healthcare	Cat#0802001
Human AB Serum	Welsh BloodTransfusion Services	N/A
TOPS Crystallography buffer screen	Jena Bioscience; Bulek et al., 2012	Custom request.
Critical Commercial Assays		
IFN-γ ELISpot (human)	Mabtech	Cat#3420-2A
IFN-γ ELISpot (mouse)	Mabtech	Cat#3321-2A
BirA biotin-protein ligase kit	Avidity	Cat#BirA500
Pierce Protein A IgG Plus Orientation Kit	Thermo Fisher	Cat#44893
RNAeasy Plus Micro Kit	QIAGEN	Cat#74034
SMARTer RACE 5′/3′ Kit	Takara Bio	Cat#634858
NEBNext Ultra DNA Library Prep Kit for Illumina	New England Biolabs	Cat#E7370
MiSeq v2 Reagent Kit	Illumina	Cat#MS-102-2001
Deposited Data		
TCR sequencing data	This paper	https://vdjdb.cdr3.net/
F11-DR1-PKY Complex	This paper	PDB: 6R0E
DR1-SGP	This paper	PDB: 6QZC

(*Continued on next page*)

Continued

REAGENT or RESOURCE	SOURCE	IDENTIFIER
DR1-QAR	This paper	PDB: 6QZD
DR1-GMF	This paper	PDB: 6QZA
R and Bash Code: Analysis and Figures	This paper	https://github.com/ALGW71/ConservedEpitopesIAV
Experimental Models: Cell Lines		
721.174.DR1 Antigen Presenting Cells	Laboratory of David Cole; Theaker et al., 2016	N/A
Experimental Models: Organisms/Strains		
HLA-DR1+ mice. Strain: Tg(HLA-DRA*0101, HLA-DRB1*0101)1Dma	Laboratory of Daniel Altman	MGI:5312109
FoxP3-DTR mice. Strain: B6.129(Cg)-Foxp3tm3 (DTR/GFP)Ayr/J	The Jackson Laboratory (Stock No: 016958)	MGI:3698131.
Oligonucleotides		
TCR-Cβ-R1 (reverse): GAGACCCTCAGGCGGCTGCTC	Rius et al., 2018	N/A
Universal Primer A (forward): TAATACGACTCACTATAGGGCAA GCAGTGGTATCAACGCAGAGT	SMARTer RACE 5′/3′ Kit (Takara Bio)	Cat#634858
TCR-Cβ-R2 (reverse, nested): TGTGTGGCCAGGCACACCAGTGTG	Rius et al., 2018	N/A
Universal Primer Short: CTAATACGACTCACTATAGGGC	SMARTer RACE 5′/3′ Kit (Takara Bio)	Cat#634858
TCR-Cα-R1 (reverse): CCATAGACCTCATGTCTAGCACAG	Rius et al., 2018	N/A
TCR-Cα-R2 (reverse, nested): GGTGAATAGGCAGACAGACTTGTC	Rius et al., 2018	N/A
Recombinant DNA		
pGMT7 expression vector	Laboratory of Andrew Sewell/ David Cole	N/A
HLA-DR1α with biotin AviTag sequence	Laboratory of David Cole/Andrew Godkin; Cole et al., 2012	Uniprot: P01903
HLA-DR1α	Laboratory of David Cole/Andrew Godkin; Cole et al., 2012	Uniprot: P01903
HLA-DR1β	Laboratory of David Cole/Andrew Godkin; Cole et al., 2012	Uniprot: P01911
F11 TCRα	Laboratory of David Cole/Andrew Godkin; Holland et al., 2018	N/A
F11 TCRβ	Laboratory of David Cole/Andrew Godkin; Holland et al., 2018	N/A
Software and Algorithms		
NetMHCIIpan 3.1	Andreatta et al., 2015	http://www.cbs.dtu.dk/services/NetMHCIIpan-3.1/
RAxML v8	Stamatakis, 2014	https://github.com/stamatak/standard-RAxML
Figtree	Laboratory of Andrew Rambaut	http://tree.bio.ed.ac.uk/software/figtree/
PyMol 2.0	Schrödinger LLC	https://pymol.org/2/
CCP4i2	Winn et al., 2011	http://www.ccp4.ac.uk/download/
REFMAC	Murshudov et al., 2011	Module of CCP4i2
Coot	Emsley and Cowtan, 2004	https://www2.mrc-lmb.cam.ac.uk/personal/pemsley/coot/
STACEI	Laboratory of Andrew Sewell	https://github.com/WhalleyT/STACEI
MiXCR	Bolotin et al., 2015	https://github.com/milaboratory/mixcr

(*Continued on next page*)

Continued

REAGENT or RESOURCE	SOURCE	IDENTIFIER
FlowJo	BD	https://www.flowjo.com/
R 3.6	The R Foundation	https://cran.r-project.org/
OLGA	Sethna et al., 2019	https://github.com/statbiophys/OLGA
GLAM2	Bailey et al., 2009	http://meme-suite.org/tools/glam2
IMGT/JunctionAnalysis	Giudicelli and Lefranc, 2011	http://www.imgt.org/IMGT_jcta/analysis
Other		
CTL Immunospot S6 Ultra	CTL Europe	Cat#S6ULTRA-V
ELISpot IP Filter Plate, 0.45 μm	Merck Millipore	Cat#MSIPS4510
HiTrap Q HP Anion Exchange Column	GE Healthcare Life Sciences	Cat#17115401
Superdex 200 Increase 10/300 GL	GE Healthcare Life Sciences	Cat#28990944
Art-Robbins Gryphon Robot	Art Robbins Instruments, LLC	Cat#620-1000-10
ARI INTELLI-PLATE 96-2 Low Volume Reservoir Plate	Art Robbins Instruments, LLC	Cat#102-0001-01
Formulatrix Rock Imager 2	Formulatrix, Inc	Cat# ROCK IMAGER 2

RESOURCE AVAILABILITY

Lead Contact

Further information and requests for resources and reagents should be directed to and will be fulfilled by the Lead Contact, Dr David Cole (ColeDK@cardiff.ac.uk).

Materials Availability

This study did not generate any unique reagents.

Data and Code Availability

All code used for TCR sequence analysis and generation of figures from crystallographic and repertoire data is available from: https://github.com/ALGW71/ConservedEpitopesIAV. TCR sequencing data is available from: https://vdjdb.cdr3.net/. All crystal datasets have been deposited in the Protein Database: https://www.rcsb.org/ under accession numbers: 6R0E, 6QZC, 6QZD, 6QZA. Raw FCS files are available through the lead contact.

EXPERIMENTAL MODEL AND SUBJECT DETAILS

Primary Cell Culture

Fresh blood was obtained from five local HLA-DR1^{+} donors (age range: 20 – 60, gender: three females and two males). Donors gave written consent (approved by Medical School Research Ethics Committee, Cardiff University). All material was handled, stored and documented in line with human tissue act regulations. PBMCs were isolated from fresh blood over ficoll gradient (Lymphoprep, Axis-Shield). Cultures were set up on day-0. Cells were resuspended at 2 M/mL in "A5" medium [RPMI-1640 (GIBCO) supplemented with 5% human AB serum (heat inactivated, Welsh Blood Transfusion Services), 2 mM l-glutamine, 100 U/mL penicillin, 100 μg/mL streptomycin (all Life Technologies)] and 100 μL (200,000 PBMC) cultured at in U-bottom 96 well plate (37°C, 5% CO2, sterile water placed in the outer wells) with peptide or peptide pool at 10 μg/mL (1 μg / 100 μL). Cell-kine (Helvetica Healthcare) was added at 10 μL per well at day-3. Media supplemented with 40 IU/mL IL-2 (Proleukin®, University Hospital of Wales pharmacy) was added at day-6 (100 μL) and replaced at day-9 (100 μL remove, then added with care not to disturb the cell pellet). Cells were used for immunoassays from day-12 up to day-21 (ELISpot only). Prior to assay, cells cultured under the same condition (peptide or peptide pool) were combined, washed 3 times in PBS before resuspension and distribution in A5 medium. For IAV peptide screens 600,000 PBMC were cultured per condition (three wells, 200,000 per well). For HLA-multimer staining 1 million PBMC were cultured per condition (five wells, 200,000 cells per well). Staining was carried out between day-12 to day-14.

Cell Lines

174.DR1 APCs from the laboratory of David Cole (Theaker et al., 2016) were cultured (37°C, 5% CO2) in RPMI-1640 (GIBCO) supplemented with 10% fetal calf serum (heat inactivated, GIBCO), 2 mM l-glutamine, 100 U/mL penicillin, 100 μg/mL streptomycin (all Life Technologies). Cells were contained in standard culture flasks and passaged (removal of between half and two thirds of the resuspended volume) every two to three days. HLA-DR expression was checked by flow cytometry with using an anti-DR antibody (clone: L243, BioLegend).

Transgenic Mice

FoxP3-DTR mice (Strain: B6.129(Cg)-*Foxp3*$^{tm3(DTR/GFP)Ayr}$/J, labeled in Figure 2 as wild-type, WT) and HLA-DR1+ mice [strain: Tg(HLA-DRA*0101,HLA-DRB1*0101)1Dma, a gift from Professor Danny Altmann, Imperial College London] were housed in scantainers on a 12 hour light/dark cycle, ventilated with HEPA filtered air and allowed access to standard mouse chow and water *ad libitum*. Each strain of mice was maintained as a homozygous colony. All mice were drug and test naive at the start of the study and all mice appeared healthy with no signs of disease. Mice had not undergone any previous procedures. Each strain was backcrossed to a C57/BL6 background for over 10 generations. Mice were kept in specific pathogen-free conditions in accordance with the United Kingdom's Home Office guidelines. All work was approved by the Animal Welfare and Ethical Review Board (AWERB) at Cardiff University. Studies followed the ARRIVE guidelines. Mice were allocated to experimental groups by age and sex-match. For infections, mice were anesthetised using isoflurane and infected intranasally. At 7 – 10 weeks of age, mice were infected intra-nasally with 1500 pfu of A/Hong Kong/X31 or 50ul of PBS as a control under light anesthesia. Body weight was recorded daily until the mice were sacrificed at day 14 post infection and spleens isolated for analysis by IFN-γ ELISpot (Mabtech). *In vivo* challenge data was collected over three repeats of the same experiment. Details of mice weight, ages and gender are given for each experiment. Exp 1: 4 DTR mice (X31), 2 DTR mice (PBS), 2 DR1 mice (X31). All female, 10 weeks old. Starting body weights 20 - 24.6 g. Co-housed between 2 and 4 mice per cage. Exp 2: 3 DR1 mice (X31), 4 DTR mice (X31), 4 DTR mice (PBS). All female, 10 weeks old. Starting body weights 20.5 – 24.6 g. Co-housed between 2 and 4 mice per cage. Exp 3: 2 DR1 (X31), 2 DR1 (PBS), 6 DTR (X31), 5 DTR (PBS). 3 male DR1 mice, 1 female DR1 mouse, 6 female DTR mice, 5 male DTR mice. 4 DR1 mice aged 9 weeks, 6 DTR mice aged 9 weeks, 5 DTR mice aged 7 weeks. Starting body weights 16.2 – 28 g. Co-housed n = 3 mice per cage.

METHOD DETAILS

Peptide libraries and pools

Peptide libraries were obtained from GL Biochem (Shanghai) Ltd as 20-mers in the crude form (50% purity). Peptide sequences overlapped by 10 amino acids (Figures S1–S3). Original sources of the sequences are as follows: Matrix Influenza A virus (A/Wilson-Smith/1933(H1N1) 252 amino acids), 24 overlapping peptides. Nucleoprotein Influenza A virus (A/Ck/HK/96.1/02 (H5N1) 401 amino acids), 39 overlapping peptides. PB1 Influenza A virus (A/Puerto Rico/8/1934(H1N1) 757 amino acids), 74 overlapping peptides. See Table S1.

Sequence numbering of influenza proteins

For the internal proteins, sequence numbering was assigned from the starting methionine referred to a reside-1 and so on. For HA the structural numbering system was used for the universal epitope $HA_{306-318}$-PKY, consistent with previous publications.

IFN-γ ELISpot

174.DR1 APCs were pulsed in a 96 well plate at a concentration of 200,000 cells per 100 μL with peptide or peptide pool at 10 μg/mL (1 μg / 100 μL) for 2 hours (37°C, 5% CO2) in RPMI 1640 medium (plus L-glutamine and antibiotics). Following pulsing, cells were washed in PBS (150 μL) three times to remove unbound peptides before resuspension in assay medium. APCs that were not pulsed with peptide (negative control for ELISpot) were incubated and washed alongside pulsed cells. 75,000 PBMC were cultured on anti-IFN-γ coated ELISPOT plate (MSIPS4510) coated with anti-IFN-γ capture antibody (1-D1K, Mabtech) with relevant 50,000 peptide pulsed APC in a total volume of 150 μL for 16 hours (37°C, 5% CO2). Plates were developed following manufacturer's protocol (Detect: 7-B6-1-Biotin, Streptavidin-ALP). Positive controls were phytohaemagglutinin-L (PHA, Sigma) and PKY-$HA_{306-318}$ (Krieger et al., 1991). Tests were run in duplicate wells, with a single negative control (PBMC and 174.DR1 APCs in the absence of peptide or PHA stimulation). Developed plates were imaged and counted using a CTL Immunospot analyzer. CTL Single Color software was used for spot counting and QC. Settings were kept constant for each reading. Assays were normalized for cumulative analysis (bar graphs displayed in Figures S1–S3) by division of individual well spots by total number of spots across all wells (minus background).

Binding algorithm prediction

NetMHCIIpan (version 3.1; http://www.cbs.dtu.dk/services/NetMHCIIpan/) was used to predict the epitope based on the strongest binding core. Sequences 20-30 amino acids in length were input in FASTA format and HLA-DRB1*0101 was the allele selected, threshold of strong and weak binders was left at default parameters, with 'print only strongest binding core' and 'sort output by affinity' checked. Output lengths of 13-17 amino acids were ranked according to predicted binding affinity and used to design shorter peptides of 13-14 amino acids in length (one peptide, QAR, was designed at 17 amino acids, 5 residues at the N-terminal flank, 2 residues at the C-terminal, in order to explore order in the N-terminal flank through X-ray crystallography, the same peptide was used in all cell assays). Shorter peptides were ordered at greater 80% purity (Peptide Protein Research Ltd.).

Production of soluble HLA-DR1 multimers

Soluble peptide-HLA-DR1 was refolded using recombinantly expressed DR1α and DR1β chains with peptide, as described in previous publications (Cole et al., 2012). Briefly, relevant HLA-DR chains, DR1α: HLA-DRA*01 (Uniprot: P01903, residues [26-207]) or

DR1α with a C-terminal biotinylation sequence (AviTag™: GLNDIFEAQKIEWHE) joined via a flexible linker (GSGG) and DR1β: HLA-DRB1*0101 (Uniprot: P01911, residues [30-219]) were cloned into the pGMT7 expression vector and expressed in Rosetta (DE3) competent BL21 *E. coli* cells (Novagen). Proteins were isolated from inclusion bodies solubilised in 8M urea buffer (20 mM Tris, pH 8.1, 0.5 mM EDTA, pH 8.1) and purified on AKTA Pure FPLC (GE Healthcare Life Sciences) using a HiTrap Q HP anion exchange column (GE Healthcare Life Sciences) over a 1M NaCl gradient. Purified DR1α and DR1β chains (5 mg/L) were refolded with peptide (0.5 mg/L) refolded in a 25% glycerol buffer solution (20 mM Tris, 1 mM EDTA, 20 mM NaCl, 1.48 g/L cysteamine hydrochloride and 0.83 g/L cystamine hydrochloride, stirred for 1 hr, followed by incubation 72 – 120 hr, 4°C). Soluble refolded monomer was concentrated, and buffer exchanged into PBS using by filtration (10 kDa MWCO concentration cassette, Sartorius AG) followed by concentration in centrifugal filter units (Merck Millipore). Conformationally intact monomer was isolated by immunoaffinity column chromatography (PBS buffer, L243 α-HLA-DR antibody immobilised on Pierce Protein A IgG Plus Orientation kit, ThermoFisher Scientific). If intended for use in HLA-multimer staining, monomers were biotinylated using a BirA biotinlyation kit (Avidity) in 10 mM TRIS, 10 mM NaCL buffer pH 7.4. Efficiency of biotinlyation was checked with a biotin shift assay, using monomer incubated with equimolar amounts of free streptavidin (20 min, RT) before analysis on SDS-PAGE (4%–12% Bis-Tris, Bolt™ Invitrogen) using loading buffer (Bolt™ LDS, Invitrogen) in the absence of a reducing agent. Biotinylated monomer was purified by size exclusion column chromatography (Superdex 200 increase 10/300 GL, GE Healthcare Life Sciences) into PBS for HLA-multimers or 10 mM TRIS, 10 mM NaCl buffer pH 7.4 for use in crystallography.

Preparation of HLA-Multimers

All pHLA monomers used in human experiments were multimerised on a dextramer backbone (‘Klickmer’, Immudex) following published methodology (Dolton et al., 2015; Tungatt et al., 2015). Per individual stain, 0.5 μg of refolded and biotinylated pHLA monomer was incubated with 2 μL of phycoerythrin (PE)-conjugated dextramer backbone solution (30 min, room temperature) and diluted with PBS to give 0.1 μg/μL of monomer, with addition of a protease inhibitor cocktail (1:100, set 1, Merck). The volume of dextramer backbone added per μg of pHLA monomer is batch dependent and the manufacturer’s guidance should be followed. The pHLA dextramers were centrifuged (> 10000 rpm, 30 s) to pellet aggregated material immediately before use. Multimers could be made on the day of staining, or up to five days before, stored at 4°C.

Human HLA-Multimer Staining

Five PBMC lines of 200,000 cells, cultured as described above, were combined (estimated as 1 million total cells) then split into three flow cytometry tubes (for test, irrelevant HLA class-II multimer and fluorescence minus one (FMO) control) and washed (800 g, 3 min) in 3 mL of FACS buffer (PBS supplemented with 10% heat inactivated FCS). Prior to HLA-multimer staining the cells were incubated with the protein kinase inhibitor Dasatanib (50 nM, 30 min, 37°C; Axon Medchem) to maximize productive staining with HLA-multimer. Dasatinib was stored at −80°C as one-use aliquots at 10 mM in DMSO. HLA-multimers (0.5 μg with respect to pHLA component) were added in a volume of 5 μL directly to PBMC lines in Dasatanib, without washing, and incubated for 30 min at 4°C. Lines were washed as above in FACS buffer and incubated with anti-PE ‘boost’ antibody (0.5 μg per stain, 10 μg/mL, 20 min, 4°C; clone PE001, BioLegend) (Tungatt et al., 2015). The ‘boost’ antibody stabilizes the pHLA multimer at the cell surface leading to enhanced staining of the cells. Cells were washed twice in PBS, then stained with violet LIVE/DEAD Fixable Dead Cell Stain, Vivid (Life Technologies) (1:40 pre-dilution in PBS, 2 μL per stain, 5 min, RT). The antibody cocktail of remaining stains was added for incubation (20 min, 4°C): anti–CD8-allophycocyanin-vio770 (1:50, clone BW135/80; Miltenyi Biotec), anti-CD4 allophycocyanin (1:50, clone M-T466; Miltenyi Biotec), anti-CD3-peridinin chlorophyll protein (PerCP) (1:50, clone BW264/56; Miltenyi Biotec); anti-CD19-Pacific blue (1:25, clone HIB19; BioLegend); and anti-CD14-Pacific blue (1:25, clone M5E2; Bio- Legend). Following antibody cocktail incubation, cells were washed twice in FACS buffer before analysis by flow cytometry. Cells were sorted on a BD FACS ARIA (BD Biosciences) with the help of central biology services (CBS) at Cardiff University. Cells were sorted directly into RLT lysis buffer (QIAGEN) supplemented with 0.5 M DTT, and frozen at −80°C for RNA extraction (RNAeasy Plus Micro Kit, QIAGEN) and cDNA isolation (SMARTer® RACE 5′/3′ Kit, Takara Bio).

Conservation Analysis

To assess conservation of the five HLA-DR1 restricted epitopes (SGP-M$1_{17\text{-}30}$; GLI-M$1_{129\text{-}142}$; QAR-M$1_{208\text{-}222}$; DPF-$NP_{273\text{-}285}$; GMF-$PB1_{410\text{-}422}$) among animal and human influenza viruses, we estimated the exact peptide match among globally circulating human, swine and avian influenza A viruses using unique amino acid sequences of MP (n = 49,755), NP (n = 51,921) and PB1 (n = 41,222) proteins of all influenza A subtypes available in NCBI GenBank. To visualize amino acid changes in the five immunogenic epitopes we reconstructed the phylogenetic relationships of full-length MP, NP and PB1 genes of influenza A viruses using the general time reversible nucleotide substitution model with gamma rate heterogeneity (GTR+G) using RAxML v8 (Stamatakis, 2014) and visualized using Figtree (http://tree.bio.ed.ac.uk/software/figtree/).

TCR sequencing

TCR sequencing was performed as previously described (Rius et al., 2018). RNA was extracted from each sample using an RNeasy Plus Micro Kit (QIAGEN) and used to make cDNA (5′/3′ SMARTer RACE kit, Takara Bio). The SMARTer approach, utilizing a Murine Moloney Leukaemia Virus (MMLV) reverse transcriptase, a 3′ oligo-dT primer and a 5′ oligonucleotide, generated cDNA templates

flanked with a known, universal anchor sequence that was targeted in subsequent PCR steps. A reverse primer specific for the TCR-α or the TCR-β constant region (CαR1 5′ CCATAGACCTCATGTCTAGCACAG-3′ or CβR1 5′-GAGACCCTCAGGCGGCTGCTC-3′, Eurofins Genomics, Germany) was then used with an anchor-specific forward primer (Takara Bio, France) in for the first PCR reaction: 2.5 μL template cDNA, 0.25 μL High Fidelity Phusion Taq polymerase, 10 μL 5X Phusion buffer, 0.5 μL DMSO (all from Thermo Fisher Scientific, UK), 1 μL dNTP (50 mM each, Life Technologies, UK), 1 μL of each primer (10 μM), and nuclease-free water to make up a total reaction volume of 50 μL. Subsequently, 2.5 μL of the first PCR products were used to set up a second PCR (reagent cocktail as above), using a nested set of primers flanked with Illumina index sequences (CαR2 5′-GGTGAATAGGCAGACAGACTTGTC-3′ or CβR2 5′-TGTGTGGCCAGGCACACCAGTGTG-3′, immediately followed by the Illumina index sequence, Eurofins Genomics, Germany). For both PCR reactions, cycling conditions were: 5 min at 94°C, 30 cycles of 30 s at 94°C, 30 s at 63°C, 90 s at 72°C, and a final 10 min extension at 72°C. Final PCR products were loaded on a 1% agarose gel and purified using the QIAEX II gel extraction kit (QIAGEN, Germany). Purified products were pooled and libraries were processed with the NEBNext Ultra Library preparation kit (New England Biolabs) and run on an Illumina MiSeq instrument using a MiSeq v2 reagent kit (Illumina). TCR gene usage was determined based on reference sequences from the Immunogenetics (IMGT) database (http://imgt.org) and all TCR gene segments were designated according to the IMGT nomenclature using MiXCR software (Bolotin et al., 2015). Only TCRs with ten reads per clonal sequence were taken forward for analysis to ensure low frequency or ambiguous sequence data was not included. Clonal expansions were not utilized to calculate gene usage frequencies or in motif analysis to ensure that these results were not impacted by any potential PCR bias during cDNA preparation. TCR sequencing data has been deposited online at https://vdjdb.cdr3.net/ (Bagaev et al., 2020).

Analysis and Visualization of TCR sequencing data in R

Following processing of raw sequencing data, information was processed and presented using R. General packages used: 'tcR' (Nazarov et al., 2015), 'ggseqlogo' (Wagih, 2017), 'gridextra', 'ggpubr', 'ggforce' and 'tidyverse' (Wickham, 2014). VJ chord diagrams: plots were created using the 'circlize' package (Gu et al., 2014) and modification of code from vdjtools (Shugay et al., 2015) to fit specific color schemes. TCR entropy: the Shannon entropy function was used from the 'tcR' package (Nazarov et al., 2015). KL distance: Background V-gene usage was kindly provided by Genomics of Adaptive Immunity laboratory (Prof Chudakov DM) and is based on data from Britanova et al. for TRB (Britanova et al., 2016) and unpublished data for TRA. The 'alazakam' package (Gupta et al., 2015) was used to quantify charge and hydrophobicity of the middle 6 amino acids across each CDR3 length.

CDR3 Motif Analysis using GLAM2 and MUSCLE

CDR3 sequences were converted to FASTA format, with the starting cysteine and terminal phenylalanine of each sequence removed for later analysis. Four sequences were less than 8 amino acids in length and were removed from the analysis (these could not be processed by GLAM2). GLAM2 was run for batches of protein sequences (p) from the command line using the following parameters: p -a 6 -b 15 -z 10 -r 4 -n 150000. A high iteration rate (-n) of 150000 over 4 runs (-r) was used (increasing -n, as recommended in the tutorial) with a minimum number of aligned columns (-a) of 6 and a maximum (b) of 15. A minimum of 10 sequences (-z) was required for any motif. Following bulk assessment of all sequences specific to each epitope using GLAM2, sequences were subdivided in subgroups using MUSCLE and GLAM2 rerun on these subgroups in order to improve the resolution of discovered motifs and find any motifs that may have been missed by a bulk analysis. As before, the starting cysteine and terminal phenylalanine were removed, and sequences placed in the AAStringSet format using the 'Biostrings' R package. The R package 'muscle' (Edgar, 2004) was run on each set of CDR3 sequences with cluster set to "neighborjoining" to produce a neighbor joining tree (from muscle iteration 2) for later analysis. Trees were read and converted to a distance object (cophenetic.phylo) using the 'ape' package (Paradis and Schliep, 2019). This object was clustered (method = "complete") and cut (h = 4) using base R to 4 subgroups. Each subgroup was processed in GLAM2 using the same settings, with rooted and unrooted phylogenetic trees visualized using the 'ape' package.

OLGA

The OLGA (Sethna et al., 2019) software was downloaded (https://github.com/statbiophys/OLGA) and run from the command line on a tab separated file of FASTA formatted sequences of TCRα and TCRβ chains using humanTRA and humanTRB models, respectively. For all data shown in Figure S7, probability of generation values were calculated from sequence alone, without V- or J-gene information being provided to the computation (this data was inspected and can be provided by the authors). This pGen computation (without V- or J- gene information) was considered, and decided upon as the authors wanted to investigate how likely each CDR3 was to arise within the repertoire, regardless of its parent V- or J-genes, thus eliminating the effect of having higher probabilities of generation which simply occur in the context of a given V- or J-gene. This was important as several repertoires exhibited strong VJ usage bias (Figure 4) which could exert this effect and prevent pGen distributions of each repertoire from being comparable.

Crystallography

Purified pHLA proteins (refolded and produced as described above, using an untagged DRA*01 chain with no biotinylation reaction) were concentrated in crystal buffer, 10 mM TRIS, 10 mM NaCl buffer pH 7.4 ($M1_{17\text{-}30}$-SGP: 8.23 mg/mL, $M1_{208\text{-}222}$-QAR: 8.00 mg/mL). Purified HLA-DR1-PKY and F11 TCR (expressed and refolded as detailed in Holland et al., 2018) were mixed at an equimolar ratio to give a total protein concentration of 6 mg/ mL in crystal buffer. The short sequence of $PB\text{-}1_{410\text{-}422}$-GMF (Figure 1B) could not

be crystallized so a longer sequence was crystallized at a concentration of 4.50 mg/mL (PGMMMGMFNMLSTVLGVSIL) using a seeding technique with crystal seeds derived from HLA-DR1-QAR (plates were set manually using a hanging drop method). Crystal trays were set up using the TOPS screen (Bulek et al., 2012) with sitting drop vapor diffusion plates. Crystallization conditions are detailed in Table S5. Each TOPS screen buffer condition was dispensed into corresponding wells of an ARI INTELLI-PLATE 96-2 low volume reservoir plate (Art Robbins Instruments, LLC) using an Art-Robbins Gryphon robot (Art Robbins Instruments, LLC.). From the screen, 60 μL was dispensed into a mother liquor well, and two dispenses of 200 nL into separate sitting drop wells. 200 nL of protein sample was dispensed into the top well containing 200 nL of a TOPS screen buffer. Plates were immediately imaged using a Formulatrix Rock Imager 2 (Formulatrix, Inc.) and incubated at 18°C, with further images taken at daily intervals to monitor crystal growth.

X-Ray Crystallographic Sample Preparation and Data Collection

Crystals were collected using 20 μm or 40 μm mounted loops (Molecular Dimensions), immediately snap frozen in liquid nitrogen. Crystals were subject to X-ray diffraction and data collection at Diamond light source (Dicot, England) (1000 diffraction images taken at 200° rotation and 0.2 s exposure time). Datasets were processed by the DLS auto-processing servers in implementing either *xia2* (3dii or 3d operations) or DIALS, full details Table S5). Processed datasets were analyzed using the program suite CCP4i2 (Collaborative Computational Project, 1994). 'Matthews' was used to obtain the number of molecules in the asymmetric unit and structures were solved with 'Phaser' using an HLA-DR1 model (1DLH) or ternary complex (1FYT) for molecular replacement (McCoy et al., 2007). Coordinates and density were refined using an iterative cycle of visualization and modeling using Coot software (Emsley and Cowtan, 2004) and refinement using REFMAC5 (Murshudov et al., 2011) until convergence of refinement statistics. The GMF structure file required a change of index to the P 3_2 2 1 space group following unsuccessful cycles of refinement in the DIALS assigned P 3_1 2 1 space group. Final coordinates were visualized using PyMOL graphics software (Delano, 2002) and contact tables generated using STACEI (https://github.com/WhalleyT/STACEI) to define the interaction distances, types and partners. Electrostatic analysis was carried out using the PyMol 2.0 plug-in APBS (Baker et al., 2001).

QUANTIFICATION AND STATISTICAL ANALYSIS

IFN-γ ELISpot assays

All tests were carried out in duplicate with a control unstimulated well. The criteria for a positive response was at least double background (value of SFC in the control well) and mean of two duplicates greater than 20 SFC. For pool screens n = 2 HLA-DR1+ donors for initial epitope mapping. Assessment of individual peptides n = 4 HLA-DR1+ donors.

Flow Cytometry

HLA-DR1+ donor (n = 5) CD3+/Live/CD4+/Dextramer+ T cell populations were quantified by gating was using two controls, an irrelevant HLA-Class-II multimer and the fluorescence minus one stain in which the PE fluorochrome was not present. Gates for the test population were set based on background staining from the irrelevant HLA-multimer for each donor. All boxplots show median and IQR, standard output from ggplot2.

TCR sequencing

Calculation of percentage frequencies of V- and J- genes for each epitope specific sample was calculated (clonal frequencies were not used, just the count for each gene within detected unique clonotypes). To calculate normalized percentage frequency across all donors, values were summed and normalized to the number of donors (3-5 depending on epitope). Enriched V or J genes were labeled on the circos plots if the mean frequency exceeded 5%. Shannon entropy of each sample was calculated using the function from the 'tcR' R package (genes numbers were tabulated for each sample and passed to this function). Kullback–Leibler (KL) distance (divergence) was calculated against background frequencies of the naive repertoire [provided by the Genomics of Adaptive Immunity laboratory (Prof Chudakov DM) and is based on data from Britanova et al. (2016) for TRB and unpublished data for TRA data, code is given at https://github.com/ALGW71/ConservedEpitopesIAV].

Cell Reports, Volume *32*

Supplemental Information

CD4$^+$ T Cells Recognize Conserved Influenza A Epitopes through Shared Patterns of V-Gene Usage and Complementary Biochemical Features

Alexander Greenshields-Watson, Meriem Attaf, Bruce J. MacLachlan, Thomas Whalley, Cristina Rius, Aaron Wall, Angharad Lloyd, Hywel Hughes, Kathryn E. Strange, Georgina H. Mason, Andrea J. Schauenburg, Sarah L. Hulin-Curtis, James Geary, Yuan Chen, Sarah N. Lauder, Kathryn Smart, Dhanasekaran Vijaykrishna, Miguel L. Grau, Mikhail Shugay, Robert Andrews, Garry Dolton, Pierre J. Rizkallah, Awen M. Gallimore, Andrew K. Sewell, Andrew J. Godkin, and David K. Cole

A

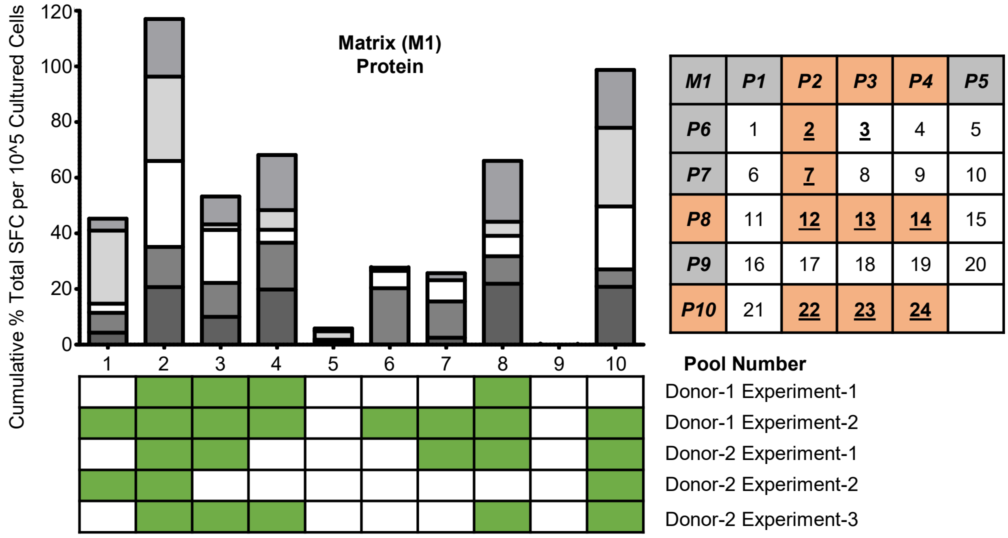

M1	*P1*	*P2*	*P3*	*P4*	*P5*
P6	1	**2**	**3**	4	5
P7	6	**7**	8	9	10
P8	11	**12**	**13**	**14**	15
P9	16	17	18	19	20
P10	21	**22**	**23**	**24**	

Identification of putatively immunogenic peptides within peptide pools. Peptide pools which elicited positive responses were cross referenced to reveal the common peptide.

B

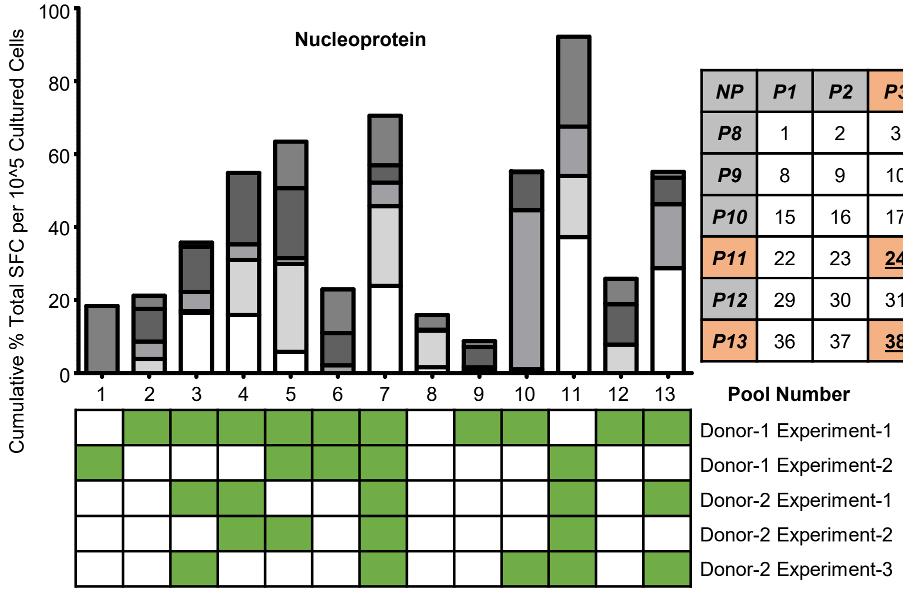

NP	*P1*	*P2*	*P3*	*P4*	*P5*	*P6*	*P7*
P8	1	2	3	4	5	6	7
P9	8	9	10	11	12	13	14
P10	15	16	17	18	19	20	21
P11	22	23	**24**	**25**	**26**	27	**28**
P12	29	30	31	32	33	34	35
P13	36	37	**38**	**39**			

C

PB1	P1	P2	P3	P4	P5	P6	P7	P8	P9
P10	1	2	3	4	5	6	7	8	9
P11	10	11	12	13	14	15	16	17	18
P12	19	20	21	22	23	24	25	26	27
P13	28	**29**	30	31	**32**	33	**34**	35	36
P14	37	**38**	39	40	**41**	42	**43**	44	45
P15	46	47	48	49	50	51	52	53	54
P16	55	56	57	58	59	60	61	62	63
P17	64	65	66	67	68	69	70	71	72
P18	73	74							

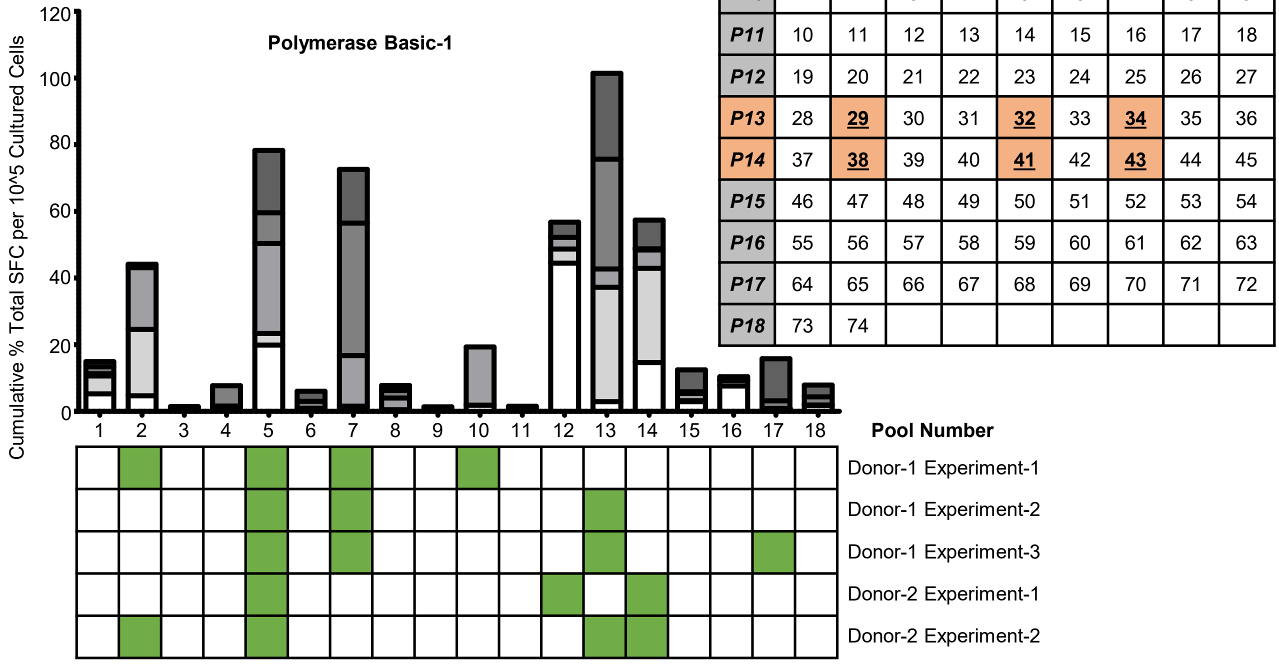

Figure S1. Summary of epitope mapping assays using peptide pools, Related to Figure 1. Results are shown by pane for each internal protein: M1-matrix (**A**), NP-nucleoprotein (**B**) and PB1-polymerase basic-1 protein (**C**). Peptide 20-mers (numbered 1, 2, 3… in the array/matrix format) overlapping by 10 amino acids were arranged into pools, and designed such that each peptide was only found in a unique combination of two pools (arrays for each protein are detailed with pool number (P1, P2…) on the top row and down the left column in gray). Each pool specific SFC result was normalised by dividing by the total number of SFC across all peptide pools in that assay to give a percentage value. Percentage values are stacked for each pool to give a cumulative representation of the responses across multiple experiments (assay repeats on blood taken at different timepoints) in two donors. The results of each experiment are summarised in a grid below the bar chart, with the response to a pool filled in green if the SFC number was defined as a positive result (greater than 20 SFC per 100,000 PBMC). The pools which were defined as immunogenic (based on shared immunogenicity in each donor), are indicated by the colour orange on the array to the right-hand side of each bar chart. Cross-referencing the array of positive pools indicated the peptides to be investigated individually (numbers underlined in orange boxes). Additional peptides were also tested that were not explicitly highlighted in two cross referenced pools but were part of a highly immunogenic pool that warranted further investigation (also underlined in orange lined boxes).

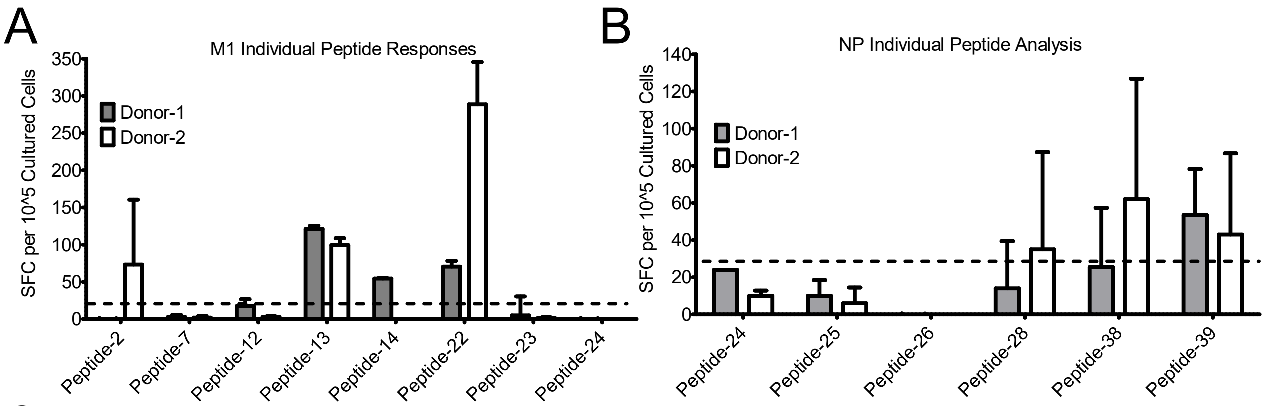

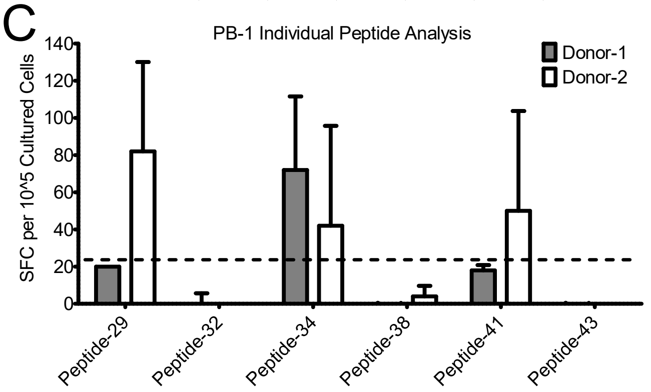

D

	Short Peptide Sequences
**M1$_{17-30}$**	SGPLKAEIAQRLED
**M1$_{129-142}$**	GLIYNRMGAVTTEV
**M1$_{208-222}$**	QARQMVQAMRTIGTHP
**NP$_{302-314}$**	DPFRLLQNSQVFS
**NP$_{417-430}$**	LPFERATIMAAFT
**NP$_{409-421}$**	PTFSVQRNLPFER
**PB-1$_{284-297}$**	NVVRKMMTNSQDT
**PB-1$_{342-354}$**	IMFSNKMARLGKG
**PB-1$_{410-422}$**	GMFNMLSTVLGVS

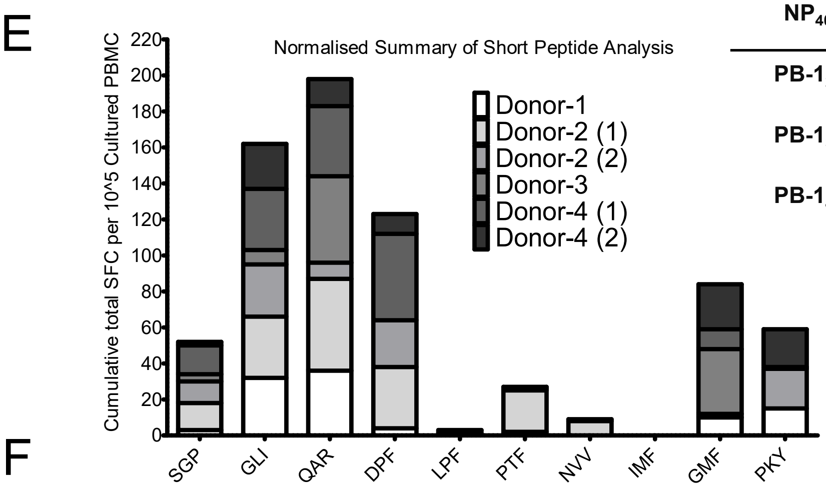

F

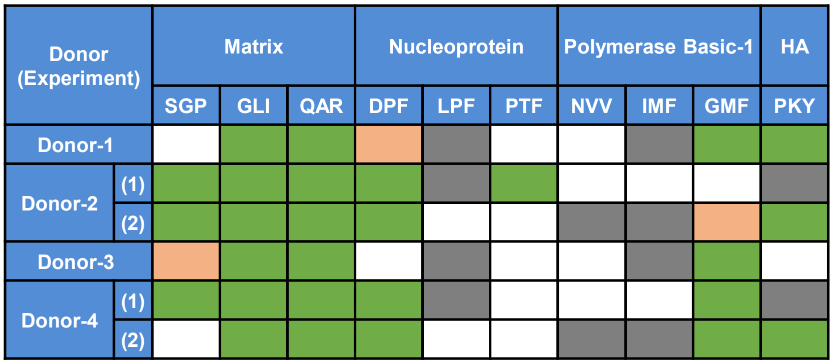

Figure S2. Individual peptide analyses of regions identified from pool assays on HLA-DR1 IFN-γ ELISpot, Related to Figure 1. (**A**) Matrix-1, (**B**) Nucleoprotein, (**C**) Polymerase Basic-1. For each assay, a line cultured against a parent pool, and shown to be reactive to that pool on IFN-γ ELISpot, was then retested with specific individual peptides from that pool. Due to limited numbers of PBMC and a broad range of testing that occurred, not all peptides were tested equal numbers of times in each donor (mean with SD error bars, donor 1: n = 2, donor 2: n = 3). Based on these results and predictions from NetMHCIIpan, short peptide sequences were designed (**D**), and used for further testing in the rest of the study. Lines were cultured with these short

peptides across 4 HLA-DR1+ donors and cumulative analysis on normalised SFC is shown (**E**), where each response was dividing by the total number of SFC across all peptide tested in that particular assay to give a percentage value. Percentage vales are stacked to give a cumulative representation of the responses across multiple assays in four donors. (**F**) Representation of each assay, with the response to a specific peptide filled in green if the SFC number was a positive result (greater than 20 SFC per 100,000 PBMC). Boxes are orange if a response was borderline (of two replicates one was just above the significance level and one was just below, but the mean was below 20 SFC). White indicates no response. Dark grey indicates not tested.

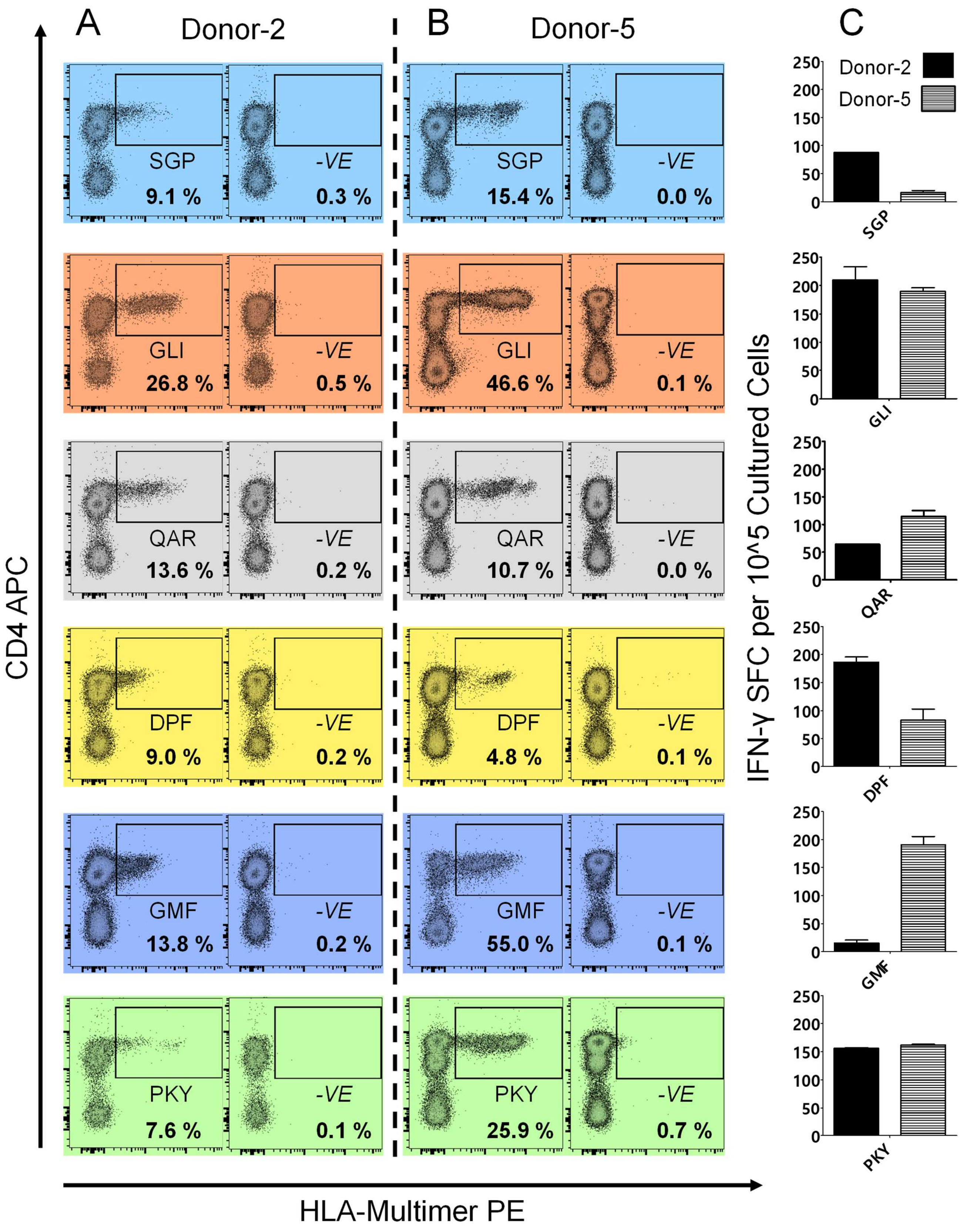

Figure S3. Comparison of HLA-Multimer staining and IFN-γ ELISpot responses in two HLA-DR1[+] donors, Related to Figure 2. Donor numbers correspond to those shown in Fig. 7 of the manuscript. HLA-multimer stains are shown alongside irrelevant HLA Class-II multimer negative (-VE) controls for donor-2 (**A**) and donor-5 (**B**) with % of CD4+ T-cells shown for each gate. Data for each epitope is shown as a colour-coded row. (**C**) IFN-γ ELISpot data for each donor and epitope is displayed

as SFC per 100,000 PBMC with background (negative control) subtracted, donor-2 in black, donor-5 in hatched bars (mean with SD error bars, n = 2).

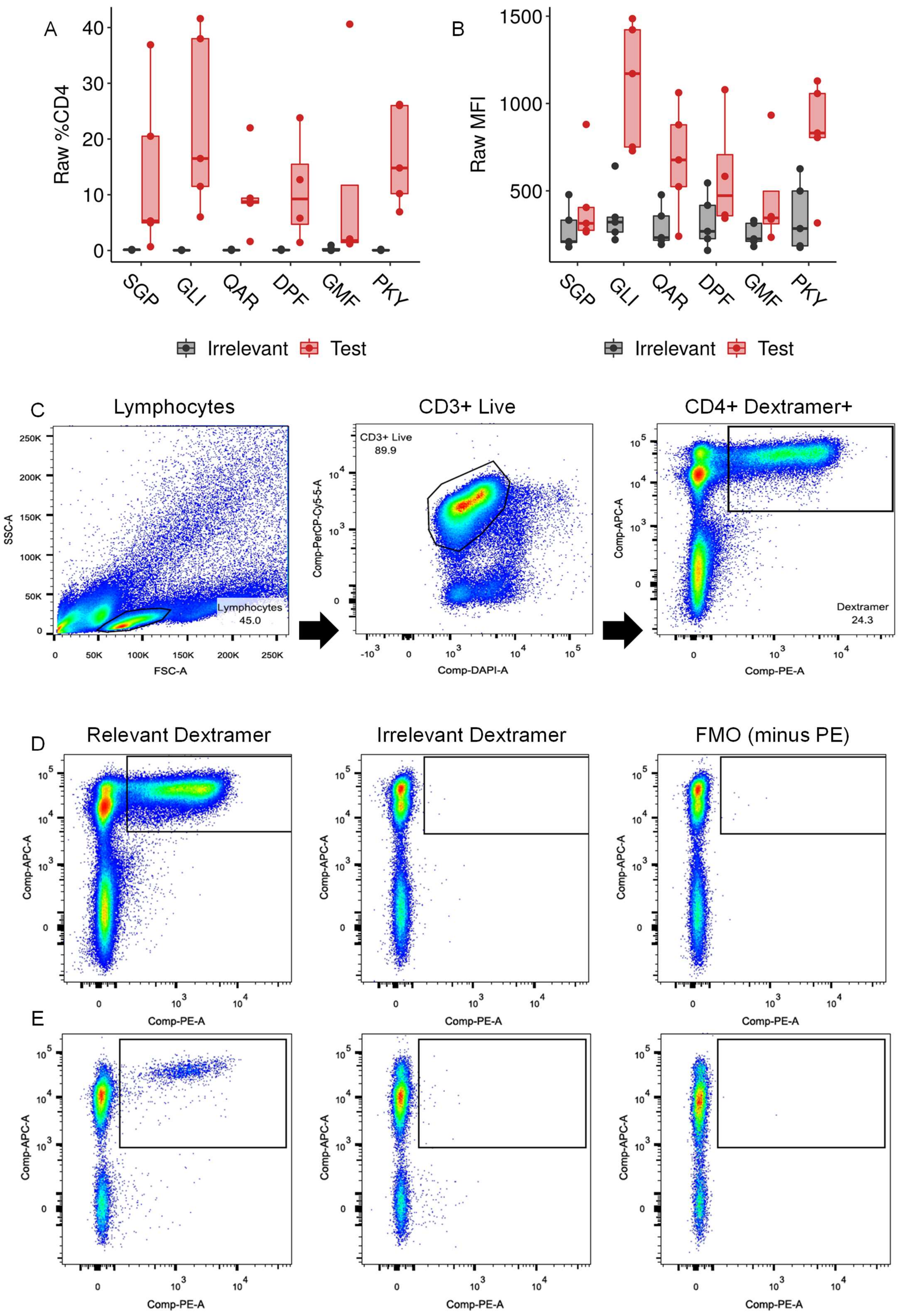

A
Raw %CD4
0
10
20
30
40
SGP
GLI
QAR
DPF
GMF
PKY
Irrelevant
Test
B
Raw MFI
500
1000
1500
SGP
GLI
QAR
DPF
GMF
PKY
Irrelevant
Test
C
Lymphocytes
SSC-A
FSC-A
Lymphocytes
45.0
CD3+ Live
CD3+ Live
89.9
Comp-PerCP-Cy5-5-A
Comp-DAPI-A
CD4+ Dextramer+
Comp-APC-A
Comp-PE-A
Dextramer
24.3
D
Relevant Dextramer
Irrelevant Dextramer
FMO (minus PE)
Comp-APC-A
Comp-PE-A
E
Comp-APC-A
Comp-PE-A

Figure S4. Additional flow cytometry data: human in vitro raw values, gating strategy and examples of irrelevant control and fluorescence minus one (FMO) stains, Related to Figure 2. Data corresponding to human in vitro staining shown in Fig. 2A-C. (**A**) Epitope-specific cells as a %CD4^{+} T cells, box plots show median, and interquartile range. (**B**) Corresponding MFI (median fluorescence intensity), values for epitope-specific populations. (**C**) Gating strategy to identify antigen specific CD4+ T-cells. (**D-E**) Example staining showing two examples of the same population stained with relevant dextramer, control irrelevant class-II dextramer (used to define the dextramer gate) and the fluorescence minus one control.

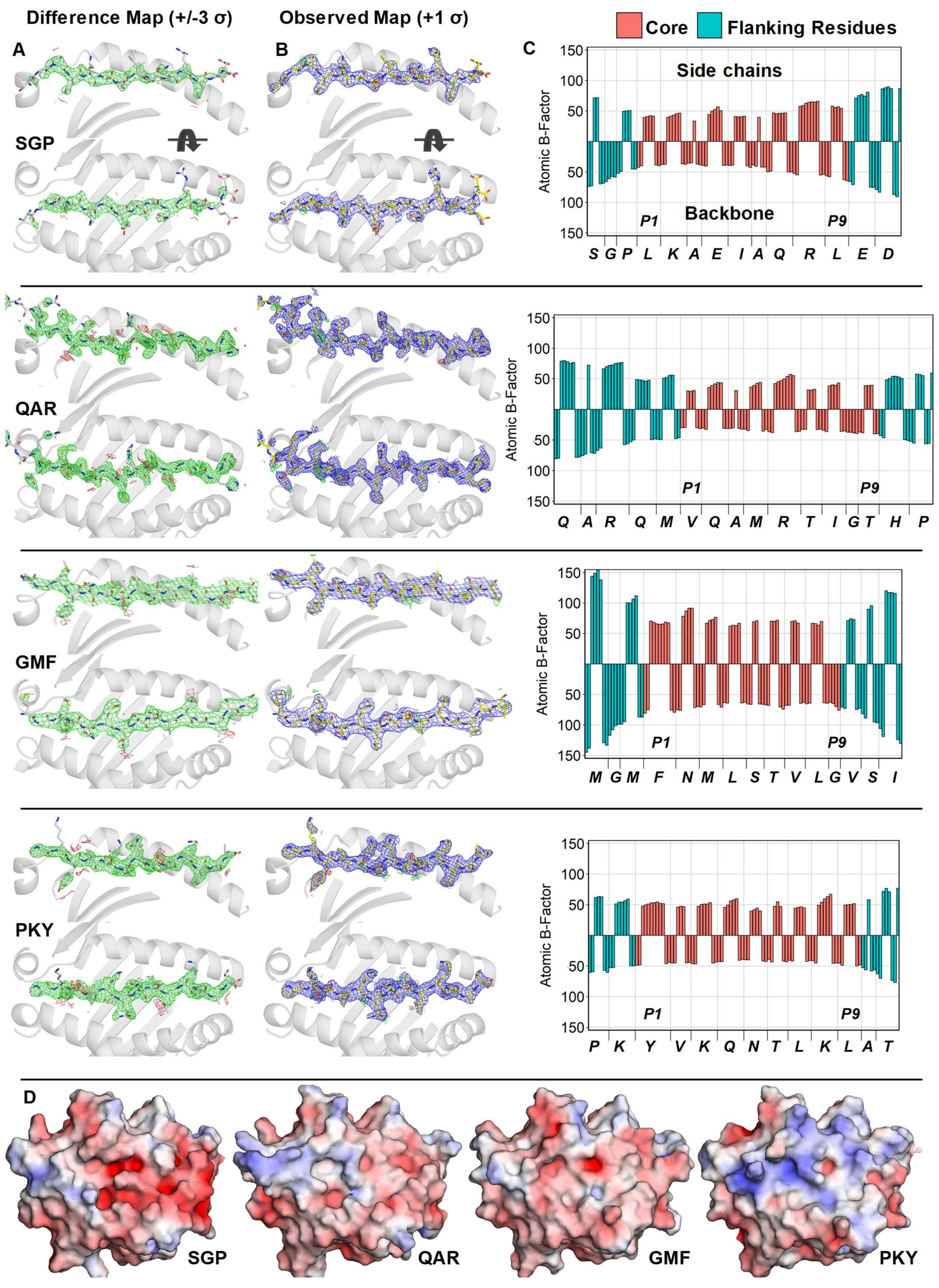

Figure S5. Electron Density of Conserved HLA-DR1 Flu Epitopes, Related to Figure 1. Column **(A)** shows the omit maps (FoFc) around each peptide in two orientations. Column **(B)** shows the final electron density (2FoFc) at the end of refinement. Observed density is displayed at 1 σ contour level, in blue. positive difference density at +3 σ is shown in green and negative difference density is shown in red at -3 σ. Column **(C)** shows a bar chart representing individual B-factors per non-H atom of the

peptide. Main chain atoms (N, Cα, C, O) bars are pointing downwards; side chains bars are pointing upwards. Panel **(D)** shows the electrostatic surface potential, calculated with PyMOL 2.0 plug-in APBS (Baker et al., 2001). Red areas are overall negatively charged, blue areas are positively charged, and grey/white areas are neutral.

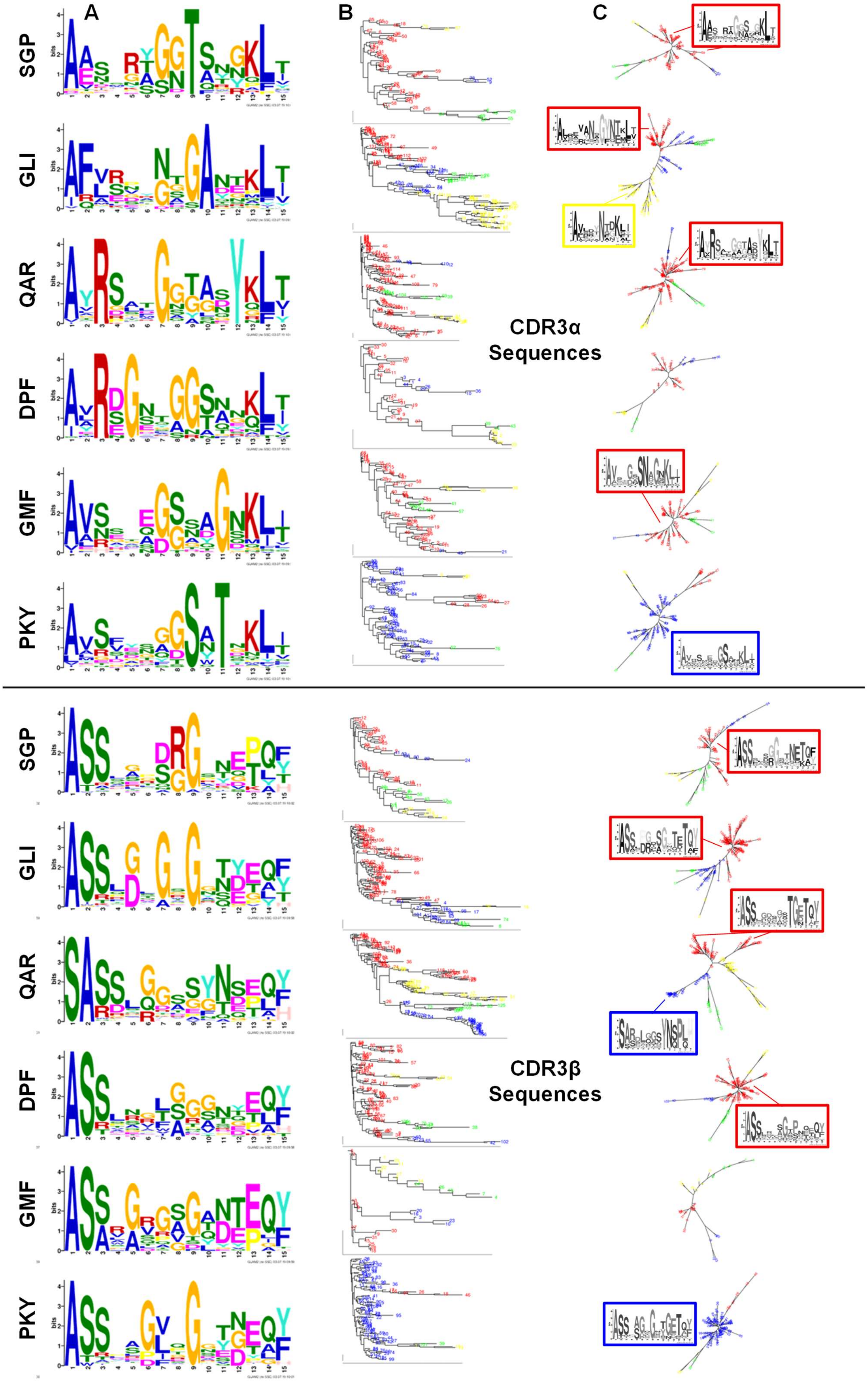

A
B
C
SGP
GLI
QAR
DPF
GMF
PKY
CDR3α Sequences
CDR3β Sequences

Figure S6. Analysis of CDR3 sequences to search for prominent motifs. Top pane shows results of CDR3α sequences, the bottom pane CDR3β sequences, Related to Figure 7. Column (**A**) GLAM2 (Gapped Alignment of Motifs) analysis performed on all CDR3 sequences in response to each epitope. The highest scoring motifs are shown for each epitope based on GLAM2 parameters detailed in methods (high iteration number and max motif length of 15 amino acids). Column (**B**) Phylogenetic analysis of CDR3 sequences in response to each epitope. Sequences were first aligned using MUSCLE and used to create a neighbour joining tree. The tree was then converted to a distance object which was cut into 4 subgroups and each tip coloured according to sub group membership (all code provided: https://github.com/ALGW71/ConservedEpitopesIAV). Column (C) Corresponding unrooted phylograms of CDR3 sequences shown in Supplementary Figure 13. GLAM2 analysis was performed on each subgroup (following methodology detailed in Chen et. al 2017) and the dominant high scoring motifs found by GLAM2 for large subgroups are displayed alongside. Colours correspond to subgroups.

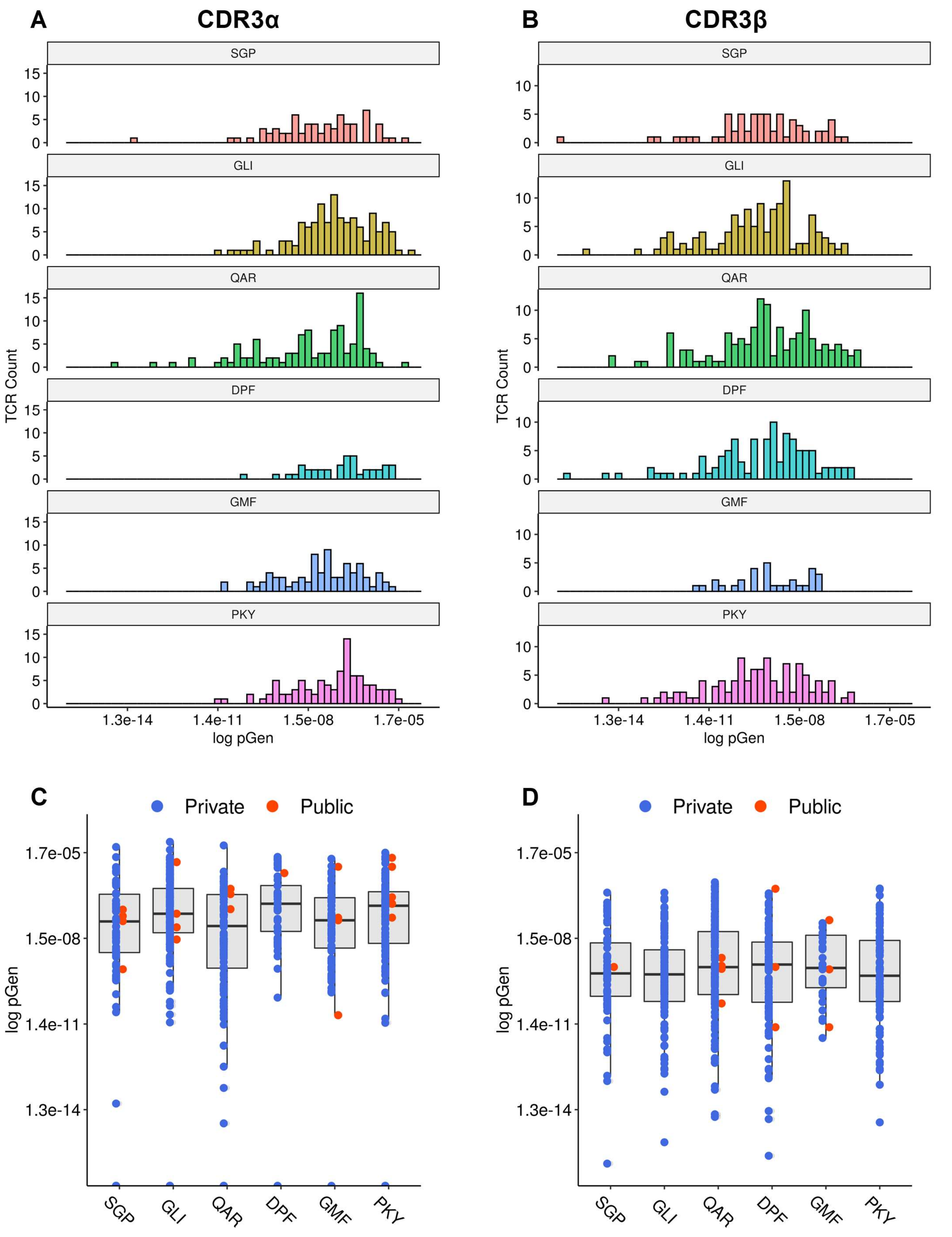

Figure S7. OLGA analysis of CDR3 Sequences, Related to Figure 7. Histograms showing CDR3α (**A**) and CDR3β (**B**) CDR3 generation probabilities (pGen) for each epitope. pGen values were calculated without VJ adjustment. Distributions distinguished by public (shared between more than one donor) or private sequences (detected in only one donor) are shown in for CDR3α (**C**) and CDR3β (**D**), with public sequences marked as orange dots.

DataS1. Detailed gene usage bar charts for each epitope and donor. Related to Figure 4. Bars are stacked by normalised percentage frequencies (percentage frequency in each donor, summed and normalised by the number of donors to allow for comparison). Colours correspond to each donor (see colour key).

Colour Key

- Donor-1
- Donor-2
- Donor-3
- Donor-4
- Donor-5

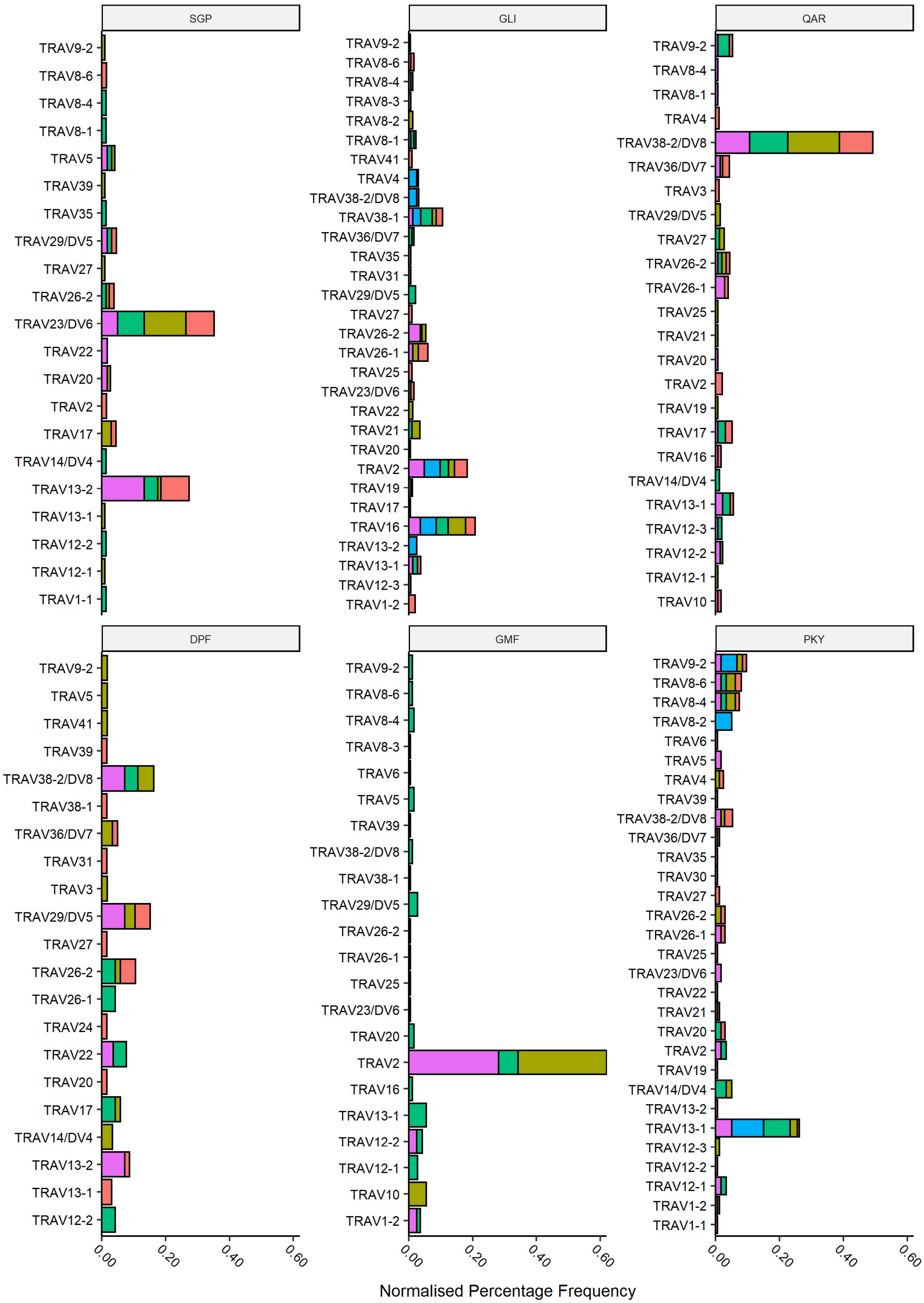

TRAV gene usage.

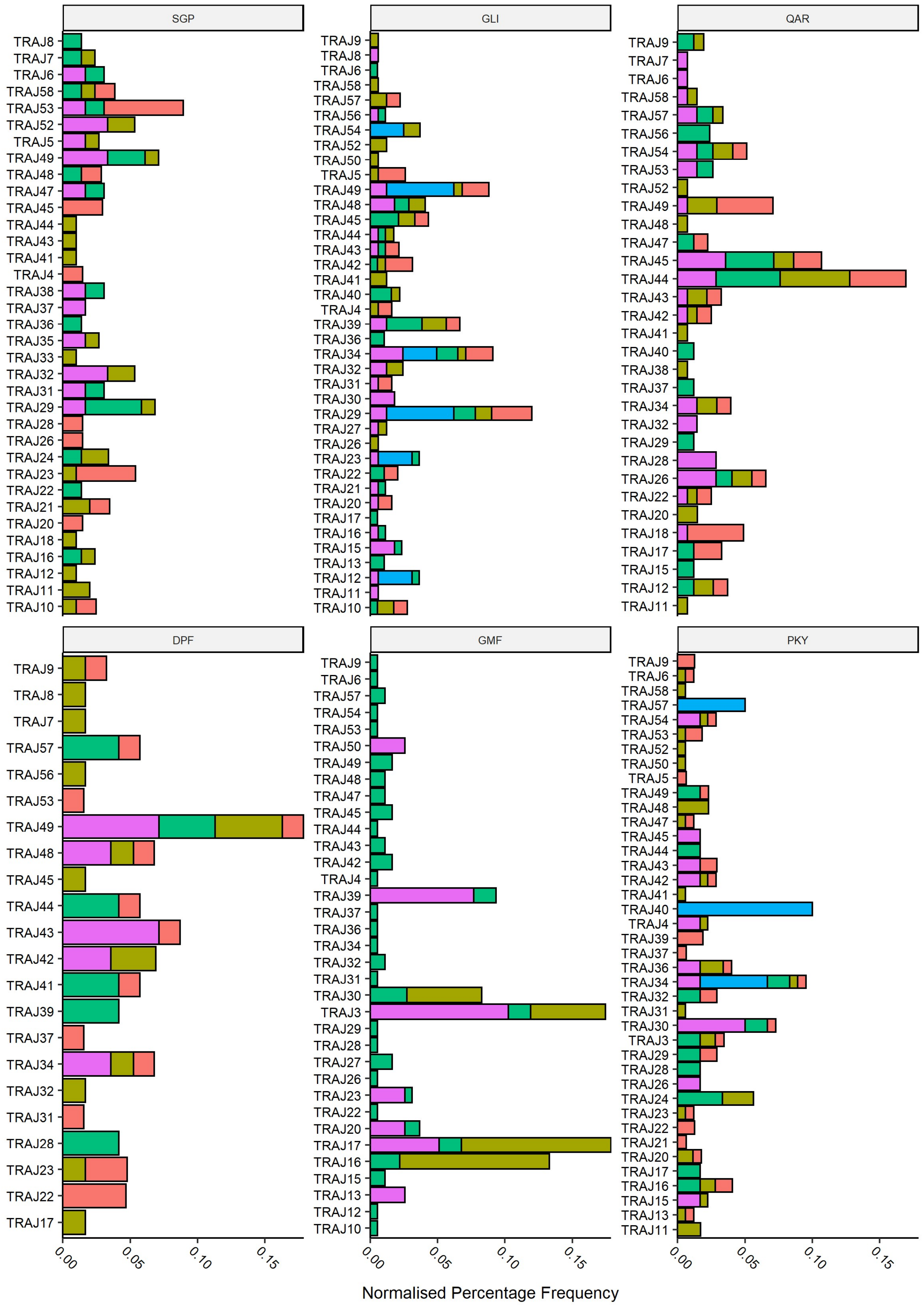

TRAJ gene usage.

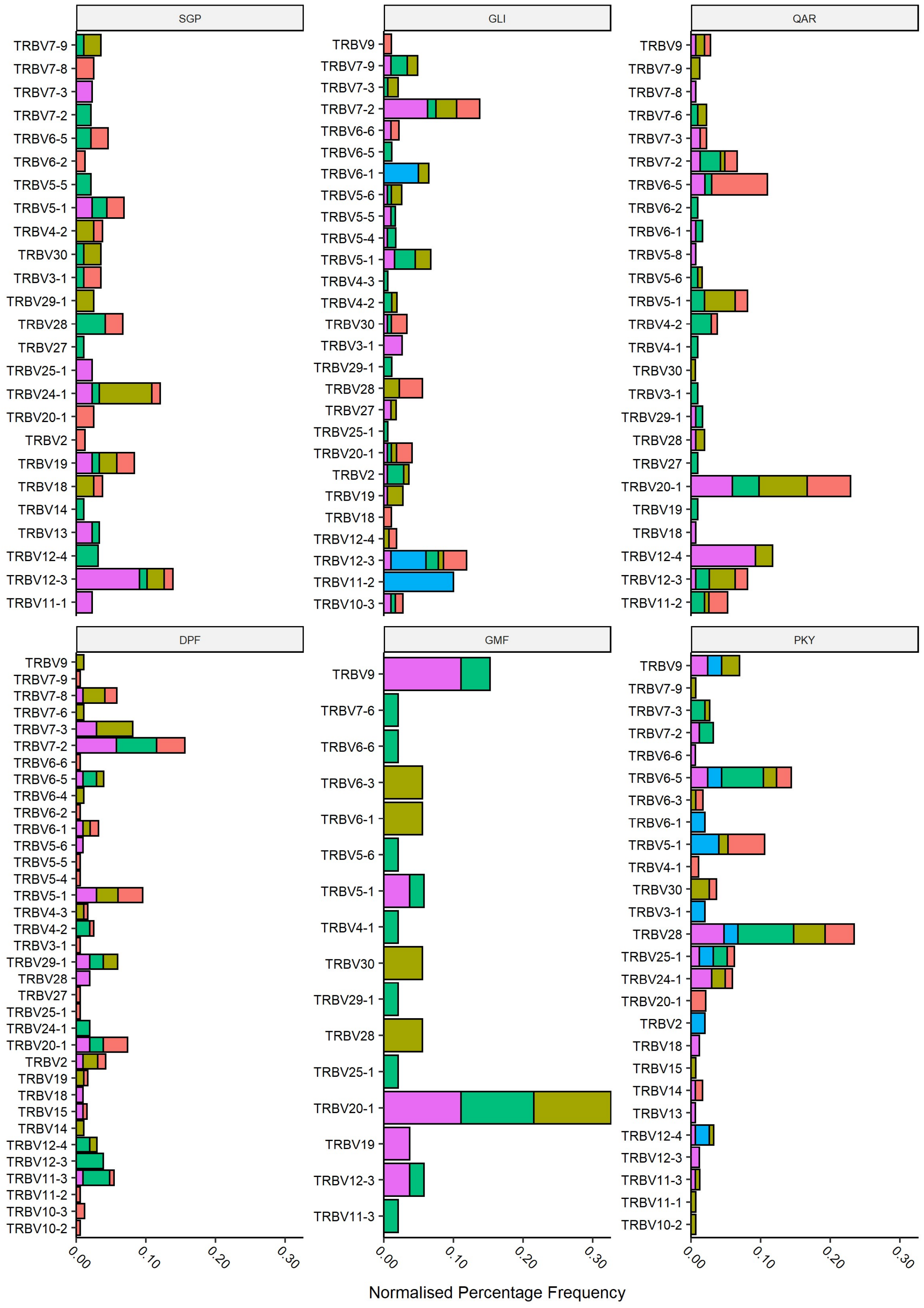

TRBV gene usage.

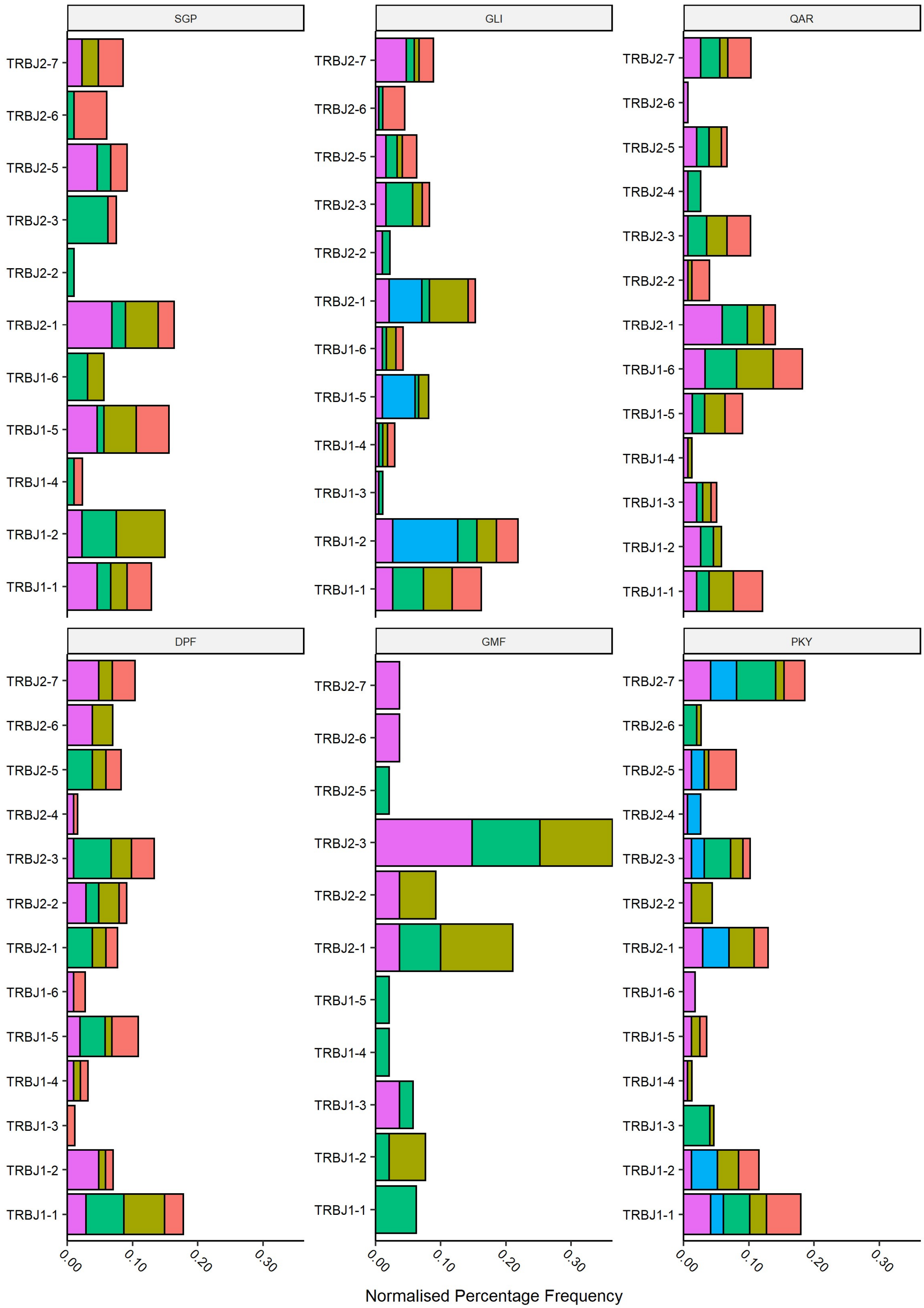

TRBJ gene usage.
